# Directional thermal emission enables efficient energy savings

**DOI:** 10.1038/s41467-026-76043-z

**Published:** 2026-07-30

**Authors:** Hao Pan, Naiqin Yi, Xuechao Li, Yuelun Leng, Yang An, Weifeng Meng, Xuezhi Zhang, Fan Jiang, Zifu Xu, Fei Xie, Weiqiang Chen, Tianji Liu, Longnan Li, Donglin Xue, Wei Li

**Affiliations:** 1 GPL Photonics Laboratory, State Key Laboratory of Luminescence Science and Technology, Changchun Institute of Optics, Fine Mechanics and Physics, Chinese Academy of Sciences, Jilin, PR China; 2 University of Chinese Academy of Sciences, Beijing, PR China; 3 Changchun Institute of Optics, Fine Mechanics and Physics, Chinese Academy of Sciences, Jilin, PR China; 4 School of Energy Science and Engineering, Harbin Institute of Technology, 92, West Dazhi Street, Harbin, PR China; 5 State Key Laboratory of Advanced Manufacturing for Optical Systems, Changchun Institute of Optics, Fine Mechanics and Physics, Chinese Academy of Sciences, Jilin, PR China

**Keywords:** Optical materials and structures, Optical physics

## Abstract

Thermal radiation, a fundamental heat transfer mechanism, is crucial for energy exchange across various applications. However, conventional radiative heat transfer systems rely on omnidirectional thermal radiation, causing energy dissipation in undesired directions and limiting radiative heat transfer efficiency. Here, we show that perfectly matched directional thermal emission between an emitter and absorber can reach a theoretical efficiency up to 100%. In a parallel-plate configuration, replacing a conventional system with a customized directional system raises efficiency from 29% to 100%. We further experimentally demonstrate directional radiative heat transfer realizes substantial energy savings of 67.8% in vacuum and 28.7% in non-vacuum conditions. Moreover, directional radiative heat transfer enables efficient heating in diverse scenarios, including vehicle heating, car paint drying, human body heating, and rail car thawing, achieving 17–46% energy savings. Our work highlights the broad applicability and energy-saving potential of directional radiative heat transfer, opening new avenues for efficient energy utilization.

## Abstract (web summary)

Conventional radiative heat transfer relies on omnidirectional thermal emission, causing energy loss in undesired directions. Here, the authors show that matched directional thermal emission enables efficient heat transfer and substantial energy savings.

## Introduction

Thermal radiation, one of the primary heat transfer mechanisms^1^, is essential for energy exchange in a wide range of applications, including solar energy utilization^2–5^, radiative cooling^6–10^, industrial heat processes^11,12^, spacecraft thermal control^13–15^, building energy savings^16–20^, and human thermal comfort^21–24^. Radiative heat transfer represents over 50% of heat loss from human body^25^ and more than 60% of heat exchange in industrial and building heating systems^26,27^. Beyond these applications, global Earth’s outgoing radiation reaches approximately 122,000 TW in Earth’s energy balance^28^, further highlighting the fundamental role of thermal radiation. Considering that global heat demand accounts for about 50% of final energy consumption along with nearly 37% of energy-related CO_2_ emissions^29^, improving the efficiency of radiative heat transfer is crucial for achieving carbon neutrality and promoting sustainability.

However, one fundamental limitation in radiative heat transfer is the inherent omnidirectional feature of thermal radiation, which leads to excessive emission or absorption in undesired directions. Although directional control of thermal radiation has been extensively investigated using various strategies, including phonon polariton gratings^30–32^, photonic crystals^33–37^, antennas^38–42^, metasurfaces^43–45^, gradient epsilon-near-zero (ENZ) materials^46–51^, and microstructures^52,53^, previous studies have primarily focused on structural design of emitters to control specific directional emission features, while the impact of these features on radiative heat transfer efficiency remains elusive. Moreover, the directional features present challenges for radiative heat transfer analysis, as conventional view factor methods inherently assume omnidirectional thermal radiation.

In this work, we extend radiative transfer theory to directional radiative transfer systems by establishing the relationship between directional emission features and radiative heat transfer efficiency, demonstrating that an ideal system with perfectly matched directional thermal emission between an emitter and absorber can reach a theoretical efficiency up to 100%. Taking a parallel plate configuration (each plate measuring 100 mm by 100 mm with a 50 mm gap) as an example, we show that replacing a conventional system with a customized directional radiative heat transfer system raises efficiency from 29 to 100%. We further experimentally demonstrate that directional radiative heat transfer leads to substantial energy savings, reducing power consumption by 67.8% in vacuum and 28.7% in non-vacuum conditions. Leveraging this directional radiative heat transfer capability, we realize efficient heating in vehicle applications, achieving 22.7% energy savings compared with a blackbody emitter. Moreover, we showcase the broad applicability and energy-saving potential of directional radiative heat transfer across various practical scenarios, including innovative applications such as car paint drying, human body heating and rail car thawing. Our work not only explores the potential of utilizing directional thermal emission to enhance radiative heat transfer efficiency, but also provides design guidelines for desired directional emission features enabled by photonic and metamaterial structures, contributing to advanced thermal management and efficient energy utilization.

## Results

### Theoretical framework and analysis of directional radiative heat transfer

**Fig. 1:**
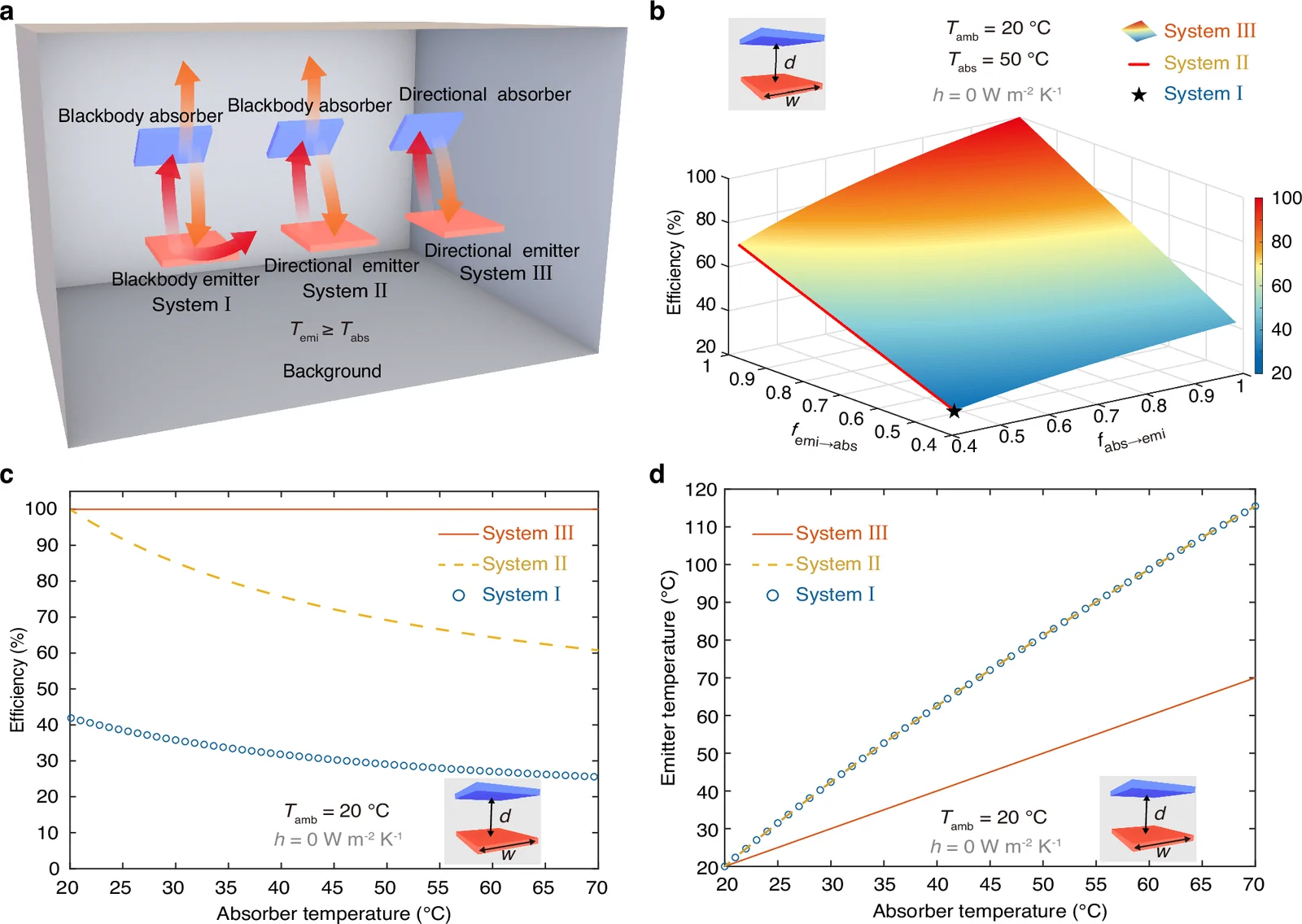
Theoretical analysis of efficient directional radiative heat transfer. **a** Radiative heat transfer in three types of systems: System Ⅰ (blackbody emitter and blackbody absorber), System Ⅱ (directional emitter and blackbody absorber), and System Ⅲ (directional emitter and directional absorber). Directional emitters and absorbers effectively suppress radiative loss to the background. **b** Radiative heat transfer efficiency η for the three systems as a function of transfer factors ${f}_{{\rm{emi}}\to {\rm{abs}}}$ and ${f}_{{\rm{abs}}\to {\rm{emi}}}$. System Ⅲ (colored surface) achieves 100% theoretical efficiency by tailoring both transfer factors ${f}_{{\rm{emi}}\to {\rm{abs}}}$ and ${f}_{{\rm{abs}}\to {\rm{emi}}}$, outperforming system Ⅰ (star-shaped marker) and system Ⅱ (red line). The ambient temperature, absorber temperature, and parasitic heat transfer coefficient are set to ${T}_{{\rm{amb}}}$ =  20 °C, ${T}_{{\rm{abs}}}$ = 50 °C, and h = 0 W m^−2^ K^−1^, respectively. Both the emitter and absorber have a width of w = 100 mm and are placed face-to-face with a separation distance of *d* = 50 mm. Radiative heat transfer efficiency η (**c**) and emitter temperature ${T}_{{\rm{emi}}}$ (**d**) as functions of absorber temperature ${T}_{{\rm{abs}}}$ for three systems. At the same absorber temperature, System Ⅲ (red solid line) not only achieves higher efficiency but also exhibits a lower emitter temperature than System Ⅰ (blue circles) and System Ⅱ (yellow dashed line). In **c**, **d**, ${f}_{{\rm{emi}}\to {\rm{abs}}}$ for Systems Ⅰ, Ⅱ, and III are 0.42, 1, and 1, respectively. The corresponding transfer factor ${f}_{{\rm{abs}}\to {\rm{emi}}}$ values are 0.42, 0.42, and 1.

Consider three types of radiative heat transfer systems involving emitters and absorbers with different directional emission features: System Ⅰ (blackbody emitter and blackbody absorber), System Ⅱ (directional emitter and blackbody absorber), and System Ⅲ (directional emitter and directional absorber) (Fig. 1a). In System Ⅰ, the fraction of radiation emitted by blackbody emitter ${A}_{{\rm{emi}}}$ that is absorbed by blackbody absorber ${A}_{{\rm{abs}}}$ is given by a geometric function known as the view factor^1^:

1 $${F}_{{\rm{emi}}\to {\rm{abs}}}=\frac{1}{{A}_{{\rm{emi}}}}{\int }_{{A}_{{\rm{emi}}}}{\int }_{{A}_{{\rm{abs}}}}\frac{\cos {\theta }_{{\rm{emi}}}\cos {\theta }_{{\rm{abs}}}}{\pi {L}^{2}}d{A}_{{\rm{abs}}}d{A}_{{\rm{emi}}}$$

where ${\theta }_{{\rm{emi}}}$ and ${\theta }_{{\rm{abs}}}$ are the angles between their surface normal and the line connecting $d{A}_{{\rm{emi}}}$ and $d{A}_{{\rm{abs}}}$ with length of L (Supplementary Fig. 1).

However, this widely used view factor approach is no longer applicable in Systems Ⅱ and Ⅲ, where at least one of the emitter and absorber exhibits directional emission features. To analyze the efficiency of directional radiative heat transfer systems, we introduce a transfer factor ${f}_{{\rm{emi}}\to {\rm{abs}}}$, representing the fraction of radiation emitted by the emitter ${A}_{{\rm{emi}}}$ that is absorbed by the absorber ${A}_{{\rm{abs}}}$

2 $${f}_{{\rm{emi}}\to {\rm{abs}}}=\frac{{\int }_{0}^{\infty }d\lambda {\int }_{{A}_{{\rm{emi}}}}{\int }_{{A}_{{\rm{abs}}}}{I}_{{\rm{BB}}}\left({T}_{{\rm{emi}}},\lambda \right){\varepsilon }_{{\rm{emi}}}\left({\theta }_{{\rm{emi}}},{\varphi }_{{\rm{emi}}},{r}_{{\rm{emi}}},\lambda \right){\alpha }_{{\rm{abs}}}\left({\theta }_{{\rm{abs}}},{\varphi }_{{\rm{abs}}},{r}_{{\rm{abs}}},\lambda \right)\frac{\cos {\theta }_{{\rm{emi}}}\cos {\theta }_{{\rm{abs}}}}{{L}^{2}}d{A}_{{\rm{abs}}}d{A}_{{\rm{emi}}}}{{\int }_{{A}_{{\rm{emi}}}}d{A}_{{\rm{emi}}}{\int }_{0}^{\infty }{I}_{{\rm{BB}}}\left({T}_{{\rm{emi}}},\lambda \right)d\lambda {\int }_{\Omega }{\varepsilon }_{{\rm{emi}}}\left({\theta }_{{\rm{emi}}},{\varphi }_{{\rm{emi}}},{r}_{{\rm{emi}}},\lambda \right)\cos {\theta }_{{\rm{emi}}}d\Omega }$$

${r}_{{\rm{emi}}}$ and ${r}_{{\rm{abs}}}$ are position vectors on the emitter and absorber, respectively. ${\varepsilon }_{{\rm{emi}}}\left({\theta }_{{\rm{emi}}},{\varphi }_{{\rm{emi}}},{r}_{{\rm{emi}}},\lambda \right)$ is the angular and spectral emissivity of the emitter at position ${r}_{{\rm{emi}}}$, ${\alpha }_{{\rm{abs}}}\left({\theta }_{{\rm{abs}}},{\varphi }_{{\rm{abs}}},{r}_{{\rm{abs}}},\lambda \right)$ is the angular and spectral absorptivity of the absorber at position ${r}_{{\rm{abs}}}$. Unlike the conventional view factor, which depends solely on geometry under the assumption of blackbody systems, the transfer factor additionally accounts for directional emission and absorption features. Therefore, it quantifies the effective fraction of radiative power emitted by the emitter that is ultimately intercepted and absorbed by the target absorber, incorporating both geometric alignment and directional radiative properties. Similarly, ${f}_{{\rm{abs}}\to {\rm{emi}}}$ represents the fraction of radiation emitted by the absorber that is absorbed by the emitter (see Supplementary Note 1 for more details). In fact, when both the emitter and absorber exhibit omnidirectional thermal emission, Eq. (2) is consistent with Eq. (1).

The transfer factor f not only characterizes the matching degree between the directional features of the emitter and absorber but also directly determines the radiative heat transfer efficiency. The efficiency can be defined as the ratio of the power ${P}_{{\rm{in}},{\rm{emi}}}$ received by the emitter from the absorber to the total power ${P}_{{\rm{out}},{\rm{emi}}}$ leaving the emitter:

3 $$\eta=\frac{{P}_{{\rm{in}},{\rm{emi}}}}{{P}_{{\rm{out}},{\rm{emi}}}}=\frac{{{P}_{{\rm{rad}},{\rm{abs}}}f}_{{\rm{abs}}\to {\rm{emi}}}}{{{P}_{{\rm{in}},{\rm{abs}}}/f}_{{\rm{emi}}\to {\rm{abs}}}+{P}_{{\rm{parasitic}}1}}$$

where ${P}_{{\rm{rad}},{\rm{abs}}}$ is the total radiation emitted by the absorber, ${P}_{{\rm{in}},{\rm{abs}}}$ is the radiation emitted by the emitter and received by the absorber, and ${P}_{{\rm{parasitic}}1}$ is the parasitic heat transfer losses from the emitter (see Supplementary Note 2 and Supplementary Fig. 2 for detailed analysis).

For illustration, we consider an emitter and an absorber, each with dimensions of 100 mm by 100 mm, placed face-to-face with a 50 mm gap in all three systems. In System Ⅰ, both the emitter and absorber exhibit omnidirectional thermal emission, which fixes the transfer factors ${f}_{{\rm{emi}}\to {\rm{abs}}}$ and ${f}_{{\rm{abs}}\to {\rm{emi}}}$ at a constant value of 0.42. These limited transfer factors cause radiative loss to the background, thus resulting in a low efficiency of ${\eta }_{{\rm{I}}}$ = 29% when the absorber temperature is heated to 50 °C (Fig. 1b). In System Ⅱ, the blackbody emitter is replaced by a directional emitter. By tailoring its directional features, ${f}_{{\rm{emi}}\to {\rm{abs}}}$ can ideally reach unity, thereby raising the efficiency ${\eta }_{{\rm{II}}}$ to 69%, even though ${f}_{{\rm{abs}}\to {\rm{emi}}}$ remains limited to 0.42 due to the absorber’s omnidirectional thermal emission. System III achieves further improvement by also replacing the blackbody absorber with a directional absorber. When both the emitter and absorber exhibit perfectly matched directional features, ${f}_{{\rm{emi}}\to {\rm{abs}}}$ and ${f}_{{\rm{abs}}\to {\rm{emi}}}$ can be optimized to unity, enabling thermal radiation to be directed precisely toward the target region while minimizing radiative losses to undesired directions. Consequently, System III reaches the theoretical upper efficiency limit of ${\eta }_{{\rm{III}}}$ = 100%. In this case, the efficiency of System III becomes independent of the absorber temperature (Fig. 1c), whereas the efficiencies of Systems Ⅰ and Ⅱ decrease significantly at large temperature differences between the absorber (or emitter) and the background. Furthermore, reducing radiative loss from the absorber not only enables System III to achieve higher efficiency but also allows the emitter to operate at a lower temperature, especially in high-temperature heating scenarios (Fig. 1d). Lower emitter temperatures are essential for safe and stable operation in energy applications. For example, in district heating networks, lowering supply and return temperatures reduces heat losses and helps maintain the network’s heat retention and hydraulic capacity^54^. Accordingly, the ability to enhance efficiency while maintaining a lower emitter temperature highlights the advantages and application potential of introducing directional thermal emission into radiative heat transfer systems. Notably, in practical radiative heat transfer scenarios, despite constraints such as parasitic heat transfer losses (Supplementary Fig. 3) and geometric configurations (Supplementary Note 3 and Supplementary Fig. 4), System III consistently achieves the highest efficiency.

### Demonstration of efficient directional radiative heat transfer

**Fig. 2:**
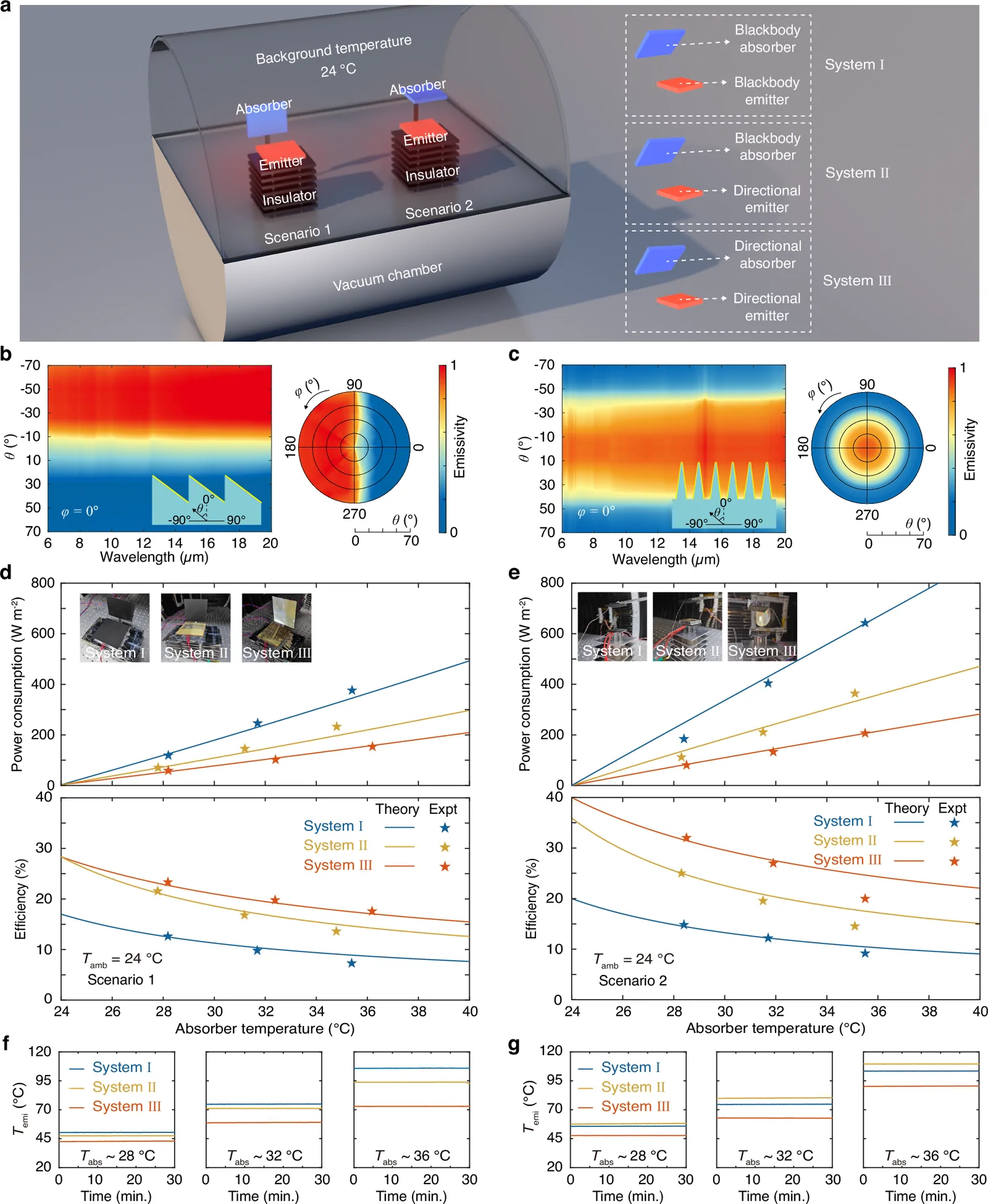
Demonstration of efficient radiative heat transfer in a vacuum environment. **a** Schematic of a customized directional radiative heating setup in a vacuum chamber with an inner surface temperature of 24 °C. Spectral angular emissivity ${\rm{\varepsilon }}\left(\lambda,\theta \right)$ (left) and angular emissivity ${\varepsilon }_{10{\rm{\mu }}{\rm{m}}}\left(\theta,\varphi \right)$ (right) at a wavelength of 10 μm varying with zenith (*θ*) and azimuth (*φ*) angles for the directional emitter in scenario 1 (**b**) and scenario 2 (**c**). Theoretical (lines) and experimental (star-shaped markers) results of power consumption ${P}_{{\rm{consum}}}$ and efficiency η for three systems at different absorber temperatures in scenario 1 (**d**) and scenario 2 (**e**). In both scenarios, System Ⅲ (red) achieves the highest efficiency and lowest power consumption compared with System Ⅱ (yellow) and System Ⅰ (blue). Measured steady-state emitter temperature ${T}_{{\rm{emi}}}$ for the three systems in scenario 1 (**f**) and scenario 2 (**g**) at different absorber temperatures ${T}_{{\rm{abs}}}$.

To demonstrate the potential of directional thermal emission for enhancing efficiency, we conducted directional radiative heat transfer experiments in both vacuum and non-vacuum conditions. The first experiment is implemented in a vacuum chamber to minimize heat convection and conduction, highlighting the effect of directional radiative heat transfer (Fig. 2a and Supplementary Note 4). Previous advances in directional thermal emission control have enabled the realization of diverse directional emission features, providing the basis for selecting and implementing the specific emission features considered in this work. Accordingly, we consider two representative radiative heat transfer scenarios and utilize two previously reported directional emitters^52,53^ with specific directional emission features to demonstrate the efficiency enhancement (Fig. 2b, c and Supplementary Figs. 5–6). In the first scenario, an emitter (100 mm by 100 mm) is placed horizontally, while an absorber of the same size is positioned vertically 10 mm from its edge. An angularly asymmetric emitter (Fig. 2b) is used as the emitter in System Ⅱ and as both the emitter and absorber in System Ⅲ to improve the transfer factor. In this configuration, the transfer factors ${f}_{{\rm{emi}}\to {\rm{abs}}}$ for Systems Ⅰ, Ⅱ, and III are 0.16, 0.27, and 0.27, respectively. The corresponding ${f}_{{\rm{abs}}\to {\rm{emi}}}$ values are 0.16, 0.16, and 0.27. Figure 2d shows the power consumption ${P}_{{\rm{consum}}}$ and efficiency η as functions of absorber temperature ${T}_{{\rm{abs}}}$ (see Supplementary Note 2 for more details). When absorbers are heated to the same temperature, System Ⅰ exhibits the lowest efficiency, while System Ⅲ achieves the highest due to the directional features of both emitter and absorber. As a result, System I consumes the most power, whereas System III requires the least. For example, when absorbers are heated to approximately 36 °C (Supplementary Fig. 7), the power consumption of Systems Ⅰ, Ⅱ, and Ⅲ are 375 W m^−2^, 232 W m^−2^, and 153 W m^−2^, respectively, corresponding to efficiencies of 7.3%, 13.6%, and 17.6% (Fig. 2d). Although the efficiency improves by only about 10%, it results in a substantial reduction of 59.2% in power consumption compared with System Ⅰ, highlighting the significant energy-saving potential of directional radiative heat transfer.

In the second scenario, an emitter (30 mm by 30 mm) is placed horizontally, with a parallel absorber of the same size positioned 30 mm above it. A directional emitter exhibits high emissivity within the angular range of −40° to 40° (Fig. 2c), is used as the emitter in System Ⅱ and as both the emitter and absorber in System Ⅲ to improve the transfer factor. In this configuration, the transfer factors ${f}_{{\rm{emi}}\to {\rm{abs}}}$ for Systems Ⅰ, Ⅱ, and III are 0.2, 0.4, and 0.4, respectively. The corresponding ${f}_{{\rm{abs}}\to {\rm{emi}}}$ values are 0.2, 0.2, and 0.4. When absorbers are heated to approximately 36 °C (Supplementary Fig. 8), the power consumption of Systems Ⅰ, Ⅱ, and Ⅲ are 643 W m^−2^, 365 W m^−2^ and 207 W m^−2^, respectively, corresponding to efficiencies of 9.2%, 14.6%, and 20% (Fig. 2e). In this case, System Ⅲ achieves a 67.8% reduction in power consumption compared with System Ⅰ. The experimental results (star-shaped markers) show reasonable consistency with the theoretical results (lines) in both Fig. 2d, e, with slight differences attributed to variations in the parasitic heat transfer coefficient between devices. Additionally, System Ⅲ consistently maintains the lowest emitter temperature in both scenarios (Fig. 2f, g).

**Fig. 3:**
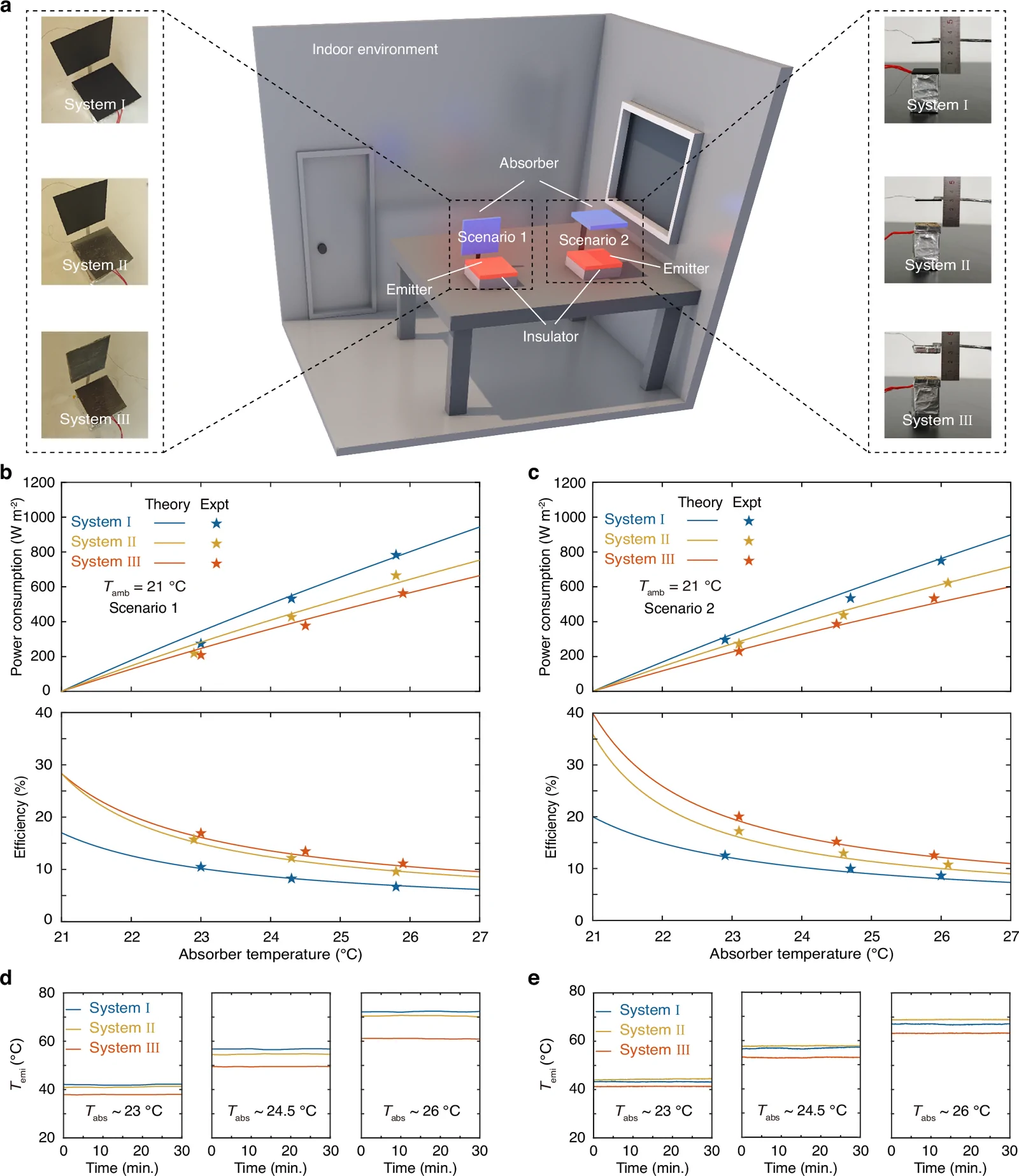
Demonstration of efficient radiative heat transfer in a typical indoor environment. **a** Schematic of the customized directional radiative heating setup in a typical indoor environment. Theoretical (lines) and experimental (star-shaped markers) results of power consumption ${P}_{{\rm{consum}}}$ and efficiency η for three systems at different absorber temperatures in scenario 1 (**b**) and scenario 2 (**c**). Although the typical indoor environment introduces additional convection and conduction heat losses, System Ⅲ (red) still achieves the highest efficiency and the lowest power consumption compared with System Ⅱ (yellow) and System Ⅰ (blue) at an ambient temperature of 21 °C. Measured steady-state emitter temperature ${T}_{{\rm{emi}}}$ for three systems in scenario 1 (**d**) and scenario 2 (**e**) at different absorber temperatures ${T}_{{\rm{abs}}}$.

The second experiment is conducted in a typical indoor environment to evaluate the practical impact of directional thermal emission (Fig. 3a). All three systems are tested simultaneously to minimize the impact of ambient temperature variations. In the first scenario, when absorbers are heated to approximately 26 °C (Supplementary Fig. 9), the power consumption of Systems Ⅰ, Ⅱ, and Ⅲ are 786 W m^−2^, 668 W m^−2^, and 565 W m⁻², respectively, corresponding to efficiencies of 6.7%, 9.6%, and 11.2% (Fig. 3b). System Ⅲ achieves a 28.1% reduction in power consumption compared with System Ⅰ. In the second scenario, when absorbers are heated to approximately 26 °C (Supplementary Fig. 10), the power consumption of Systems Ⅰ, Ⅱ, and Ⅲ are 752 W m^−2^, 625 W m^−2^ and 536 W m^−2^, respectively, corresponding to efficiencies of 8.7%, 10.8%, and 12.6% (Fig. 3c). Similarly, System Ⅲ achieves a 28.7% reduction in power consumption compared with System Ⅰ. Notably, although the typical indoor environment introduces additional convection and conduction heat losses, the System Ⅲ still achieves the highest efficiency and the lowest power consumption (Supplementary Fig. 11). Moreover, System Ⅲ still maintains the lowest emitter temperature (Fig. 3d, e), consistent with its behavior observed in the vacuum chamber.

### Directional radiative heating in vehicle applications

**Fig. 4:**
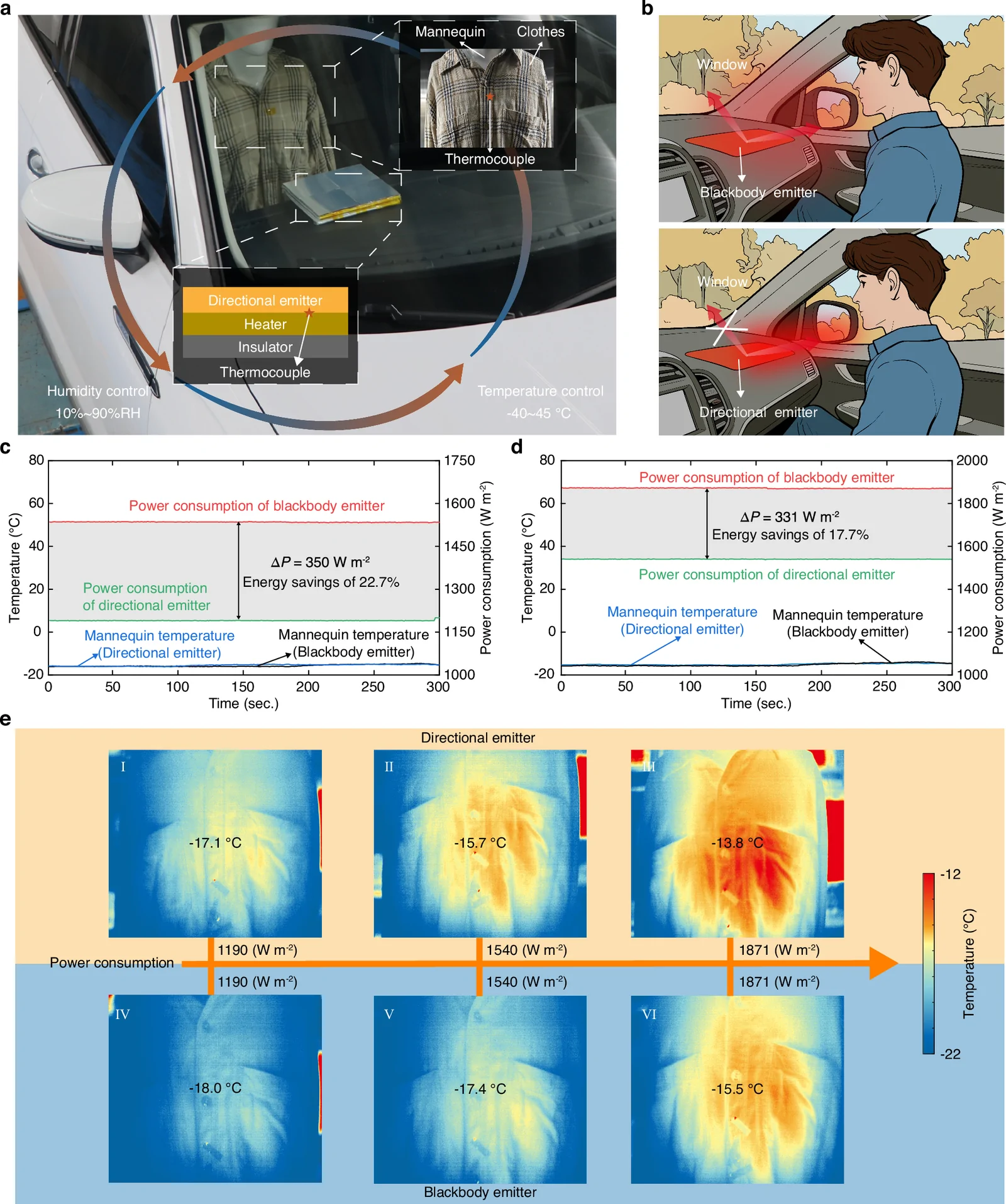
Directional radiative heating performance in a vehicle. **a** Photograph of the directional radiative heating setup in an environmental simulation chamber (ESC) which controls ambient temperature (−40 to 45 °C) and relative humidity (10 to 90%) outside the vehicle. **b** Schematic of a blackbody emitter and a directional emitter placed on the dashboard to heat a mannequin inside the vehicle. Power consumption of the blackbody emitter (red line) and directional emitter (green line) when heating the mannequin to approximately −17.2 °C (**c**) and −15.6 °C (**d**). The directional emitter achieves a 22.7% reduction in power consumption at −17.2 °C and a 17.7% reduction at −15.6 °C compared with the blackbody emitter. **e** Infrared images of the mannequin heated by directional and blackbody emitters under different power consumption. Under the same power consumption, the directional emitter produces a higher mannequin temperature than the blackbody emitter.

As electric vehicles (EVs) become increasingly widespread, efficient cabin heating has become an important challenge because EVs lack the waste heat available in internal combustion engine (ICE) vehicles. Consequently, radiative heating is attracting growing interest as a potential strategy for improving heating efficiency and localized thermal comfort in EVs^55^. In this context, we further evaluate the potential of directional radiative heat transfer for efficient vehicle heating using an environmental simulation chamber (ESC), which allows precise control of ambient temperature (−40 °C to 45 °C) and relative humidity (10 to 90%) (Fig. 4a). To simulate winter conditions, the chamber is set to −20 °C with 50% relative humidity. A directional emitter (Fig. 2b) and a blackbody emitter are placed separately on the dashboard to heat a mannequin inside the vehicle (Fig. 4b). Heating the mannequin to about −17.2 °C (Fig. 4c) and −15.6 °C (Fig. 4d) requires power consumption of 1540 W m^−2^ and 1871 W m^−2^ for the blackbody emitter, and 1190 W m^−2^ and 1540 W m^−2^ for the directional emitter, respectively. The corresponding reductions in power consumption represent significant energy savings of 22.7% and 17.7% for the directional emitter. Moreover, under the same power consumption, the directional emitter reaches a significantly higher temperature than the blackbody emitter (Supplementary Fig. 12). As a result, the mannequin heated by the directional emitter is warmer than that heated by the blackbody emitter. The efficient radiative heating performance of the directional emitter can be further visualized through an IR camera (Fig. 4e). To investigate heating performance under different ambient temperatures, we repeated the tests at an ambient temperature of 0 °C and obtained consistent results (Supplementary Fig. 13). Our results highlight the potential of directional radiative heating in vehicle applications, enabling selectively warming of targeted regions without the need to heat the entire vehicle cabin. This approach can serve as a complement to conventional HVAC systems by providing localized thermal comfort and is emerging as a promising strategy to improve heating efficiency in EVs.

### Energy savings in various practical scenarios

**Fig. 5:**
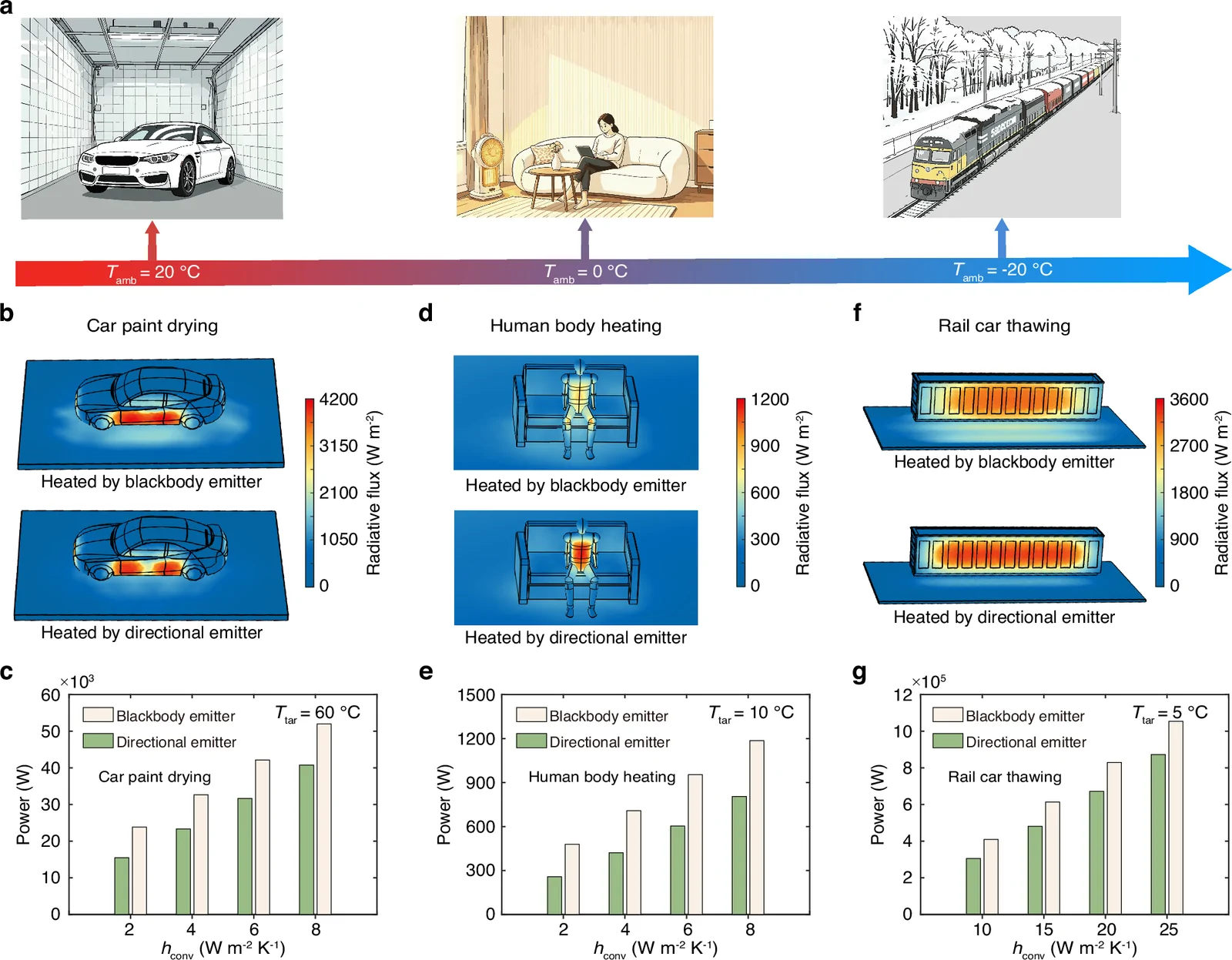
Efficient radiative heat transfer in various practical scenarios. **a** Energy-saving potential of directional radiative heat transfer is evaluated under varying ambient temperatures and convection coefficients for three scenarios: car paint drying, human body heating, and rail car thawing. Radiative flux distributions (**b**, **d**, **f**) and heating power consumption (**c**, **e**, **g**) for achieving target temperatures ${T}_{{\rm{tar}}}$ of 60 °C, 10 °C, and 5 °C in car paint drying, human body heating, and rail car thawing, respectively. The directional emitter concentrates radiative flux on the target area, achieving significant energy savings, whereas the blackbody emitter heats both the target and undesired areas. The radiative flux represents the total radiation received per unit area. Ambient temperatures are set to 20 °C for car paint drying (**b**, **c**), 0 °C for human body heating (**d**, **e**), and −20 °C for rail car thawing (**f**, **g**). Convection coefficients ${h}_{{\rm{conv}}}$ for each scenario are determined based on realistic conditions^56,57^.

We further showcase the broad applicability and energy-saving potential of directional radiative heat transfer across various energy applications, including industrial heat processes, human thermal comfort and transportation. Taking car paint drying, human body heating, and rail car thawing as examples (see Fig. 5a and Supplementary Figs. 14–16, and Supplementary Table 1 for more details), we calculated radiative flux distributions and heating power consumption across a range of convection coefficients ${h}_{{\rm{conv}}}$ under realistic conditions^56,57^. A directional emitter (see Supplementary Fig. 17 for its angular emissivity) and a blackbody emitter were used to heat the target area to the same temperature, enabling direct comparison of the energy-saving performance. For car paint drying (indoor environments, ${T}_{{\rm{amb}}}$ = 20 °C, ${h}_{{\rm{conv}}}$ = 2–8 W m^−2^ K^−1^), the radiative flux distribution indicates that the blackbody emitter heats not only the car paint but also the surrounding objects, leading to significant radiative loss (Fig. 5b). In contrast, the directional emitter primarily directs radiative flux toward the paint, achieving 22–35% energy savings compared with the blackbody emitter (Fig. 5c). Similar improvements are observed in human body heating (indoor environments, ${T}_{{\rm{amb}}}$ = 0 °C, ${h}_{{\rm{conv}}}$ = 2–8 W m^−2^ K^−1^) (Fig. 5d, e) and rail car thawing (outdoor environments, ${T}_{{\rm{amb}}}$ = -20 °C, ${h}_{{\rm{conv}}}$ = 10–25 W m^−2^ K^−1^) (Fig. 5f, g), where directional emitters achieve 32–46% and 17–25% energy savings, respectively. Notably, the energy-saving analysis is based on existing capabilities for directional control of thermal radiation, indicating significant potential for further improvement and highlighting directional radiative heat transfer as a promising strategy for substantial energy savings.

## Discussion

We have demonstrated that introducing directional thermal emission into radiative heat transfer systems can significantly enhance efficiency and enable substantial energy savings. The proposed theoretical framework reveals the impact of directional thermal emission on system efficiency and provides a practical strategy for optimizing radiative heat transfer in systems with complex geometries. By tailoring directional thermal emission to confine energy exchange exclusively between the emitter and absorber, our approach precisely directs heat flux to the target area while minimizing energy dissipation in undesired directions. Although convection and conduction diminish the contribution of radiation, our results clearly show that directional radiative heat transfer can still achieve significant energy savings under realistic conditions. Additionally, directional radiative heat transfer enables a lower emitter temperature, which is critical for safe and stable operation in various energy applications^54^. Leveraging rapid advances in photonics^37,58^, metamaterials^42,59^, and metasurfaces^30–36,38–40,43–50,52,53,60,61^, various directional thermal emitters are expected to emerge for a wide range of applications such as spacecraft thermal control^13–15^, building energy savings^16–20^, and thermal management of electronic devices^62,63^. In particular, driven by advances in thermal radiation control technologies related to thermal cycling durability^64,65^, large-area fabrication and integration^66,67^, directional radiative heat transfer is expected to become increasingly feasible for real-world deployment, offering a pathway toward advanced thermal management and efficient energy utilization.

## Methods

### Fabrication

The directional emitters in scenarios 1 and 2 were fabricated by a template molding technique. In scenario 1, a precision-machined metal template (an asymmetric sawtooth grating with a period of 1 mm and an aspect ratio of 2/3) was used to transfer our designed structure to a polymer substrate. In scenario 2, the metal template consisted of a periodic array of truncated cones with a 1.5 mm period, a top radius of 0.5 mm, and a height of 6 mm. Polydimethylsiloxane (PDMS, Sylgard 184) was selected as the substrate due to its excellent thermosetting properties. We first mixed the PDMS polymer and curing agent in a weight ratio of 10:1 and stirred evenly to obtain a uniform mixture. The uniform PDMS mixture was then poured into the corresponding template molds and cured in an oven at 80 °C for 1 h to form the structures. For the directional emitter in scenario 1, a 10 nm chromium (Cr) film as an adhesion layer and a 200 nm silver (Ag) film were directionally deposited only on the slanted surface using electron beam evaporation deposition with the sawtooth grating placed at an appropriate angle. This Ag film on the slanted surface is used to suppress the infrared radiation from the substrate. For the directional emitter in scenario 2, a 30 nm Cr adhesion layer and a 500 nm Ag film were sequentially deposited over the entire periodic conical surface using magnetron sputtering, followed by coating the bottom of each conical cavity with high emissivity paint to achieve directional thermal emission.

### Characterization

The optical properties of the samples were characterized in the long-wavelength infrared spectral range (6–20 μm). An FTIR equipped with a gold integrating sphere (A562, Bruker) was used to measure the infrared absorptance. The detection range of the equipped infrared detector (LN2-MCTs and RT-DLaTGS) is 350–8000 cm^−1^. The angle-resolved thermal emission spectrums of the two directional emitters were performed using a customized angle-resolved thermal emission spectrum measurement (ATESM) system (see Supplementary Note 5 and Supplementary Fig. 18 for more details). Thermographic images were taken using an infrared camera (Ti480 PRO, Fluke) working at a wavelength range of 7.5–14 μm.

### Directional radiative heat transfer tests

The performance of directional radiative heat transfer was evaluated in a vacuum chamber, where both the vacuum level and temperature could be controlled accurately. The vacuum chamber has a diameter of 1.2 m, with its inner surface coated in high emissivity paint. The vacuum level was kept at approximately 6 × 10^−6 ^Torr and the ambient temperature was maintained at 24 °C. High emissivity paint was used as both the blackbody emitter and absorber for the heating experiments (Supplementary Fig. 19). Polyimide resistance heaters were attached to the backs of the emitters for providing heating power. The emitters were placed on a thermal insulation device to minimize heat losses (Supplementary Note 4 and Supplementary Fig. 20). Temperatures of all samples were monitored by K-type thermocouples connected to a temperature data logger (RDXL6SD, Omega) outside the vacuum chamber through a thermocouple feedthrough. Thermocouples were fixed at the center of both the emitter and absorber backs, covered with aluminum foil to prevent radiative heat loss. For radiative heat transfer experiments conducted in a typical indoor environment, the convection coefficient was approximately 7 W m^−2^ K^−1^. Field tests for the vehicle application were performed in an ESC, where temperature and relative humidity were precisely controlled. We set the ambient temperature to −20 °C and the relative humidity to 50% to simulate winter climate conditions. We separately placed a directional emitter and a blackbody emitter, both measuring 200 mm by 200 mm, on the dashboard to heat a mannequin inside the vehicle. The temperatures of the mannequin and emitters inside the vehicle were also monitored using K-type thermocouples. All thermocouples were calibrated before the measurements. Power consumption data were collected using a power supply connected to a laptop, and measurements continued until steady-state conditions were reached.

### Energy-saving calculation

The energy-saving potential of directional thermal emission is evaluated in three scenarios (car paint drying, human body heating, and rail car thawing). In each scenario, the background is assumed to be an ideal blackbody that perfectly absorbs all incident radiation and emits thermal radiation uniformly across all wavelengths and directions. The directional emitter shown in Fig. 2c was redesigned with an emission zenith angle (θ) ranging from 0° to 25° (Supplementary Fig. 17). The model includes several simplifications, including a steady-state assumption and the neglect of conductive and radiative losses from the back of the emitter. We assess the energy-saving performance of three typical radiative heating applications using practical scenario parameters (Supplementary Table 1). The temperature of the target area is defined as the average temperature of its surface. We calculated the heating power consumption of emitters by the Supplementary Equation 17.

## Supplementary information


Supplementary Information
Transparent Peer Review file


## Source data


Source Data


## Data Availability

The data generated in this study are provided in the Source Data file. Source data are provided with this paper.
