# Supplementary material for: Directional thermal emission enables efficient energy savings
Source: Nat Commun. 2026 Jul 30;17:9261. doi: 10.1038/s41467-026-76043-z (PMC13526865; doi:10.1038/s41467-026-76043-z)
Supplement: Supplementary file 1 [file 41467_2026_76043_MOESM1_ESM.pdf]

Supplementary Information for

Directional thermal emission enables efficient energy savings

Hao Pan (潘昊)[1,2], Naiqin Yi (移乃琴)[1,2], Xuechao Li (李学超)[3], Yuelun Leng (冷岳伦)[3], Yang An (安阳)[1,2], Weifeng Meng (孟炜峰)[1,4], Xuezhi Zhang (张学志)[1,2], Fan Jiang (江帆)[3], Zifu Xu (徐子富)[1,2], Fei Xie (谢非)[1,2*], Weiqiang Chen (陈伟强)[1,2], Tianji Liu (刘天际)[1,2], Longnan Li (李龙男)[1,2], Donglin Xue (薛栋林)[2,5*], & Wei Li (李炜)[1,2*]

[1]GPL Photonics Laboratory, State Key Laboratory of Luminescence Science and Technology, Changchun Institute of Optics, Fine Mechanics and Physics, Chinese Academy of Sciences, Changchun, Jilin, 130033, P. R. China.

[2]University of Chinese Academy of Sciences, Beijing, 100049, China.

[3]Changchun Institute of Optics, Fine Mechanics and Physics, Chinese Academy of Sciences, Changchun, Jilin, 130033, P. R. China.

[4] School of Energy Science and Engineering, Harbin Institute of Technology, 92, West Dazhi Street, Harbin, 150001, P. R. China.

[5]State Key Laboratory of Advanced Manufacturing for Optical Systems, Changchun Institute of Optics, Fine Mechanics and Physics, Chinese Academy of Sciences, Changchun, Jilin, 130033, P. R. China.

*Correspondence to weili1@ciomp.ac.cn; xiefei@ciomp.ac.cn; xuedl@ciomp.ac.cn

This Supplementary Information file includes:

Supplementary Notes 1 to 5

Supplementary Figs. 1 to 20

Supplementary Table 1

Supplementary References

Supplementary Note 1: Radiative transfer factor f

As shown in equation (2) of the main text, the transfer factor $f_{\mathrm{emi}\to\mathrm{abs}}$ represents the fraction of radiation emitted by emitter A_{emi} that is received by absorber A_{abs}:

$$f_{\mathrm{emi}\to\mathrm{abs}} = \frac{\int_0^\infty d\lambda \int_{A_{\mathrm{emi}}} \int_{A_{\mathrm{abs}}} I_{\mathrm{BB}}(T_{\mathrm{emi}},\lambda)\varepsilon_{\mathrm{emi}}(\theta_{\mathrm{emi}},\varphi_{\mathrm{emi}},r_{\mathrm{emi}},\lambda)\alpha_{\mathrm{abs}}(\theta_{\mathrm{abs}},\varphi_{\mathrm{abs}},r_{\mathrm{abs}},\lambda)\frac{\cos\theta_{\mathrm{emi}}\cos\theta_{\mathrm{abs}}}{L^2}dA_{\mathrm{abs}}dA_{\mathrm{emi}}}{\int_{A_{\mathrm{emi}}} dA_{\mathrm{emi}} \int_0^\infty I_{\mathrm{BB}}(T_{\mathrm{emi}},\lambda)d\lambda \int_\Omega \varepsilon_{\mathrm{emi}}(\theta_{\mathrm{emi}},\varphi_{\mathrm{emi}},r_{\mathrm{emi}},\lambda)\cos\theta_{\mathrm{emi}}d\Omega} \tag{S1}$$

where the $I_{\mathrm{BB}}(T_{\mathrm{emi}},\lambda) = \frac{2hc^2}{\lambda^5}\frac{1}{e^{hc/(\lambda k_{\mathrm{B}}T_{\mathrm{emi}})}-1}$ is the spectral radiance of an ideal blackbody, h is Planck's constant, k_{B} is the Boltzmann constant, c is the speed of light, T_{emi} is the emitter temperature and λ is the wavelength. $\int_\Omega \mathrm{d}\Omega = \int_0^{2\pi} d\varphi \int_0^{\frac{\pi}{2}} d\theta \sin\theta$ is the angular integral over a hemisphere, r_{emi} and r_{abs} are position vectors on the emitter and absorber, respectively. $\varepsilon_{\mathrm{emi}}(\theta_{\mathrm{emi}},\varphi_{\mathrm{emi}},r_{\mathrm{emi}},\lambda)$ is the angular and spectral emissivity of the emitter at position r_{emi} , $\alpha_{\mathrm{abs}}(\theta_{\mathrm{abs}},\varphi_{\mathrm{abs}},r_{\mathrm{abs}},\lambda)$ is the angular and spectral absorptivity of the absorber at position r_{abs}. θ_{emi} and θ_{abs} are the angles between their surface normal and the line connecting dA_{emi} and dA_{abs} with length of L. The azimuth angle φ_{emi} (φ_{abs}) is measured between the x_{emi} (x_{abs}) axis on the A_{emi} (A_{abs}) and the projection of the line connecting dA_{emi} and dA_{abs} onto the A_{emi} (A_{abs}) (Supplementary Fig. 1). The transfer factor is calculated by integrating the radiative exchange between all interacting differential surface elements of the emitter and absorber over angle, wavelength, and surface area while accounting for directional emission and absorption features. Therefore, it quantifies the effective fraction of radiative power emitted by the emitter that is ultimately intercepted and absorbed by the target absorber, thereby providing a generalized framework for analyzing radiative heat transfer in systems with arbitrary geometric configurations and directional emission features.

Similarly, $f_{\mathrm{abs}\to\mathrm{emi}}$ represents the fraction of the radiation emitted by absorber A_{abs} that is received by emitter A_{emi}:

$$f_{\mathrm{abs}\to\mathrm{emi}} = \frac{\int_0^\infty d\lambda \int_{A_{\mathrm{emi}}} \int_{A_{\mathrm{abs}}} I_{\mathrm{BB}}(T_{\mathrm{abs}},\lambda)\varepsilon_{\mathrm{abs}}(\theta_{\mathrm{abs}},\varphi_{\mathrm{abs}},r_{\mathrm{abs}},\lambda)\alpha_{\mathrm{emi}}(\theta_{\mathrm{emi}},\varphi_{\mathrm{emi}},r_{\mathrm{emi}},\lambda)\frac{\cos\theta_{\mathrm{emi}}\cos\theta_{\mathrm{abs}}}{L^2}dA_{\mathrm{abs}}dA_{\mathrm{emi}}}{\int_{A_{\mathrm{abs}}} dA_{\mathrm{abs}} \int_0^\infty I_{\mathrm{BB}}(T_{\mathrm{abs}},\lambda)d\lambda \int_\Omega \varepsilon_{\mathrm{abs}}(\theta_{\mathrm{abs}},\varphi_{\mathrm{abs}},r_{\mathrm{abs}},\lambda)\cos\theta_{\mathrm{abs}}d\Omega} \tag{S2}$$

where T_{abs} is the absorber temperature, $\varepsilon_{\mathrm{abs}}(\theta_{\mathrm{abs}},\varphi_{\mathrm{abs}},r_{\mathrm{abs}},\lambda)$ is the angular and spectral emissivity of the absorber at position r_{abs}, $\alpha_{\mathrm{emi}}(\theta_{\mathrm{emi}},\varphi_{\mathrm{emi}},r_{\mathrm{emi}},\lambda)$ is the angular and spectral absorptivity of the emitter at position r_{emi}.

Supplementary Note 2: Theoretical analysis of radiative heat transfer

As discussed in the main text, we consider a radiative heat transfer system in thermal equilibrium, consisting of an emitter and absorber in background with temperature of T_{bg} (Supplementary Fig. 2). The total power leaving the emitter A_{emi} is:

$$P_{\mathrm{out,emi}} = P_{\mathrm{rad,emi}} + P_{\mathrm{parasitic1}} \tag{S3}$$

$P_{\mathrm{rad,emi}}$ is the total radiation emitted by the emitter:

$$P_{\mathrm{rad,emi}} = P_{\mathrm{emi\to abs}} + P_{\mathrm{emi\to bg}} \tag{S4}$$

The radiation emitted by the emitter toward the absorber $P_{\mathrm{emi\to abs}}$ can be expressed as:

$$P_{\mathrm{emi\to abs}} = \int_{A_{\mathrm{emi}}} dA_{\mathrm{emi}} \int_0^\infty I_{\mathrm{BB}}(T_{\mathrm{emi}}, \lambda) d\lambda \int_{\Omega_{\mathrm{e\to a}}} \varepsilon_{\mathrm{emi}}(\theta_{\mathrm{emi}}, \varphi_{\mathrm{emi}}, r_{\mathrm{emi}}, \lambda) \cos\theta_{\mathrm{emi}}\, d\Omega \tag{S5}$$

where $\Omega_{\mathrm{e\to a}}$ is the solid angle with which absorber is seen from surface element dA_{emi}, T_{emi} is the emitter temperature

And the radiation emitted by the emitter toward the background $P_{\mathrm{emi\to bg}}$ can be expressed as:

$$P_{\mathrm{emi\to bg}} = \int_{A_{\mathrm{emi}}} dA_{\mathrm{emi}} \int_0^\infty I_{\mathrm{BB}}(T_{\mathrm{emi}}, \lambda) d\lambda \int_{\Omega_{\mathrm{e\to b}}} \varepsilon_{\mathrm{emi}}(\theta_{\mathrm{emi}}, \varphi_{\mathrm{emi}}, r_{\mathrm{emi}}, \lambda) \cos\theta_{\mathrm{emi}}\, d\Omega \tag{S6}$$

where $\Omega_{\mathrm{e\to b}}$ is the solid angle with which background is seen from surface element dA_{emi}.

In Supplementary Equation 3, $P_{\mathrm{parasitic1}}$ is the parasitic heat transfer losses from the emitter:

$$P_{\mathrm{parasitic1}} = h\left(T_{\mathrm{emi}} - T_{\mathrm{bg}}\right) \tag{S7}$$

Here, the parasitic heat transfer coefficient h can be expressed as[1]

$$h = h_{\mathrm{conv}} + h_{\mathrm{rad}} + h_{\mathrm{cond}} \tag{S8}$$

where h_{conv} is the convection coefficient, h_{rad} is the radiative heat loss coefficient and h_{cond} is the conduction coefficient.

Similarly, $P_{\mathrm{out,abs}}$ is the total power leaving the absorber:

$$P_{\mathrm{out,abs}} = P_{\mathrm{rad,abs}} + P_{\mathrm{parasitic2}} \tag{S9}$$

$P_{\mathrm{rad,abs}}$ is the total radiation emitted by the absorber A_{abs}:

$$P_{\mathrm{rad,abs}} = P_{\mathrm{abs\to emi}} + P_{\mathrm{abs\to bg}} \tag{S10}$$

The radiation emitted by the absorber toward the emitter $P_{\mathrm{abs\to emi}}$ can be expressed as:

$$P_{\mathrm{abs\to emi}} = \int_{A_{\mathrm{abs}}} dA_{\mathrm{abs}} \int_0^\infty I_{\mathrm{BB}}(T_{\mathrm{abs}}, \lambda) d\lambda \int_{\Omega_{\mathrm{a\to e}}} \varepsilon_{\mathrm{abs}}(\theta_{\mathrm{abs}}, \varphi_{\mathrm{abs}}, r_{\mathrm{abs}}, \lambda) \cos\theta_{\mathrm{abs}}\, d\Omega \tag{S11}$$

where $\Omega_{\mathrm{a}\to\mathrm{e}}$ is the solid angle with which emitter is seen from surface element dA_{abs}, T_{abs} is the absorber temperature.

And the radiation emitted by the absorber toward the background $P_{\mathrm{abs}\to\mathrm{bg}}$ can be expressed as:

$$P_{\mathrm{abs}\to\mathrm{bg}} = \int_{A_{\mathrm{abs}}} dA_{\mathrm{abs}} \int_0^\infty I_{\mathrm{BB}}(T_{\mathrm{abs}}, \lambda) d\lambda \int_{\Omega_{\mathrm{a}\to\mathrm{b}}} \varepsilon_{\mathrm{abs}}(\theta_{\mathrm{abs}}, \varphi_{\mathrm{abs}}, r_{\mathrm{abs}}, \lambda) \cos\theta_{\mathrm{abs}}\, d\Omega \quad \text{(S12)}$$

where $\Omega_{\mathrm{a}\to\mathrm{b}}$ is the solid angle with which background is seen from surface element dA_{abs}.

Similarly, we can calculate the parasitic heat transfer coefficient of the absorber by the Supplementary Equation 8. The parasitic heat transfer losses from the absorber $P_{\mathrm{parasitic2}}$ is given by:

$$P_{\mathrm{parasitic2}} = h\left(T_{\mathrm{abs}} - T_{\mathrm{bg}}\right) \quad \text{(S13)}$$

Accordingly, the radiative heat transfer efficiency in equilibrium can be defined using the following equation:

$$\eta = \frac{P_{\mathrm{in,emi}}}{P_{\mathrm{out,emi}}} = \frac{P_{\mathrm{rad,abs}} f_{\mathrm{abs}\to\mathrm{emi}}}{P_{\mathrm{in,abs}}/f_{\mathrm{emi}\to\mathrm{abs}} + P_{\mathrm{parasitic1}}} \quad \text{(S14)}$$

The radiation emitted by the absorber and received by the emitter $P_{\mathrm{in,emi}}$ is given by:

$$P_{\mathrm{in,emi}} = \int_0^\infty d\lambda \int_{A_{\mathrm{emi}}} \int_{A_{\mathrm{abs}}} I_{\mathrm{BB}}(T_{\mathrm{abs}}, \lambda) \varepsilon_{\mathrm{abs}} \alpha_{\mathrm{emi}} \frac{\cos\theta_{\mathrm{emi}} \cos\theta_{\mathrm{abs}}}{L^2} dA_{\mathrm{abs}} dA_{\mathrm{emi}} \quad \text{(S15)}$$

The radiation emitted by the emitter and received by the absorber $P_{\mathrm{in,abs}}$ is given by:

$$P_{\mathrm{in,abs}} = \int_0^\infty d\lambda \int_{A_{\mathrm{emi}}} \int_{A_{\mathrm{abs}}} I_{\mathrm{BB}}(T_{\mathrm{emi}}, \lambda) \varepsilon_{\mathrm{emi}} \alpha_{\mathrm{abs}} \frac{\cos\theta_{\mathrm{emi}} \cos\theta_{\mathrm{abs}}}{L^2} dA_{\mathrm{abs}} dA_{\mathrm{emi}} \quad \text{(S16)}$$

And the heating power consumption of the emitter can be calculated using the following equation:

$$P_{\mathrm{consum}} = P_{\mathrm{out,emi}} - P_{\mathrm{in,emi}} - P_{\mathrm{bg-emi}} \quad \text{(S17)}$$

where $P_{\mathrm{bg-emi}}$ is the radiation emitted by the background A_{bg} and received by the emitter:

$$P_{\mathrm{bg-emi}} = \int_0^\infty d\lambda \int_{A_{\mathrm{emi}}} \int_{A_{\mathrm{bg}}} I_{\mathrm{BB}}\left(T_{\mathrm{bg}}, \lambda\right) \varepsilon_{\mathrm{bg}} \alpha_{\mathrm{emi}} \frac{\cos\theta_{\mathrm{emi}} \cos\theta_{\mathrm{bg}}}{L^2} dA_{\mathrm{bg}} dA_{\mathrm{emi}} \quad \text{(S18)}$$

$\varepsilon_{\mathrm{bg}}\left(\theta_{\mathrm{bg}}, \varphi_{\mathrm{bg}}, r_{\mathrm{bg}}, \lambda\right)$ is the angular and spectral emissivity of the background at position r_{bg}.

Supplementary Note 3: Radiative heat transfer efficiency η under different geometric configurations

To clarify the impact of geometric factors on radiative exchange, we have analyzed the dependence of radiative heat transfer efficiency η on several key parameters, including separation distance, relative orientation, and lateral misalignment.

First, we consider an emitter placed face-to-face with an absorber, each with dimensions of 100 mm by 100 mm. As the separation distance D increases from 100 to 150 and 200 mm, the corresponding transfer factors $f_{\mathrm{emi}\to\mathrm{abs}}$ and $f_{\mathrm{abs}\to\mathrm{emi}}$ of System I decrease from 0.20 to 0.11 and 0.07, respectively, leading to efficiencies of 8.7%, 3.0%, and 1.3% (Supplementary Fig. 4a). This clearly indicates that, in conventional blackbody systems, efficiency is strongly limited by separation distances and decreases rapidly as the distance increases. In contrast, at a separation distance of 200 mm, System II optimizes the directional features of the emitter according to the geometric configuration, increasing the transfer factor $f_{\mathrm{emi}\to\mathrm{abs}}$ from 0.07 to 1 and thereby improving efficiency from 1.3% to 18.9%. System III further achieves the theoretical upper limit of 100% efficiency by simultaneously optimizing both $f_{\mathrm{emi}\to\mathrm{abs}}$ and $f_{\mathrm{abs}\to\mathrm{emi}}$.

Second, we consider an emitter with a relative angle β of 0°, 45° and 60° to the absorber, at a fixed separation of 100 mm and identical dimensions of 100 mm by 100 mm. As the relative angle increases, the transfer factors $f_{\mathrm{emi}\to\mathrm{abs}}$ and $f_{\mathrm{abs}\to\mathrm{emi}}$ of System I decrease from 0.20 to 0.11 and 0.08, respectively, leading to efficiencies of 8.7%, 3.0%, and 1.7% (Supplementary Fig. 4b). At a relative angle of 60°, System II increases the transfer factor $f_{\mathrm{emi}\to\mathrm{abs}}$ from 0.08 to 1, thereby improving efficiency from 1.7% to 21.2%, while System III reaches the maximum theoretical efficiency of 100% by optimizing both $f_{\mathrm{emi}\to\mathrm{abs}}$ and $f_{\mathrm{abs}\to\mathrm{emi}}$.

Third, we consider a face-to-face configuration with lateral misalignments L of 0, 100, and 200 mm, with both emitter and absorber having dimensions of 100 mm by 100 mm. As the lateral misalignment increases, the transfer factors $f_{\mathrm{emi}\to\mathrm{abs}}$ and $f_{\mathrm{abs}\to\mathrm{emi}}$ of System I decrease from 0.20 to 0.09 and 0.02, respectively, leading to efficiencies of 8.7%, 2.1%, and 0.1% (Supplementary Fig. 4c). At a lateral misalignment of 200 mm, System II enhances the transfer factor $f_{\mathrm{emi}\to\mathrm{abs}}$ to 1, increasing the efficiency from 0.1% to 5.9%, while System III consistently maintains the theoretical maximum efficiency of 100%. Overall, these results highlight that Systems II and III mitigate the sensitivity to geometric variations through directional thermal emission, thereby demonstrating the geometric robustness and broad applicability of our framework.

Supplementary Note 4: Thermal design of radiative heat transfer systems in scenario 1 and scenario 2

As shown in Figs. 2 and 3 of the main text, a thermal insulation device was used to minimize the parasitic heat losses[1]. The parasitic heat transfer coefficient h for the experimental setup can be estimated using Supplementary Equation 8. The first experiment was conducted in a vacuum chamber (~10^{-6} Torr) to eliminate heat losses through convection, making the convection coefficient negligible[2]. The convection coefficient $h_{\text{conv-indoor}}$ of the second experiment is estimated to be approximately 7 W m^{-2} K^{-1} for a typical indoor environment[3].

We first evaluate radiative heat loss coefficient in scenario 1, where an emitter (100 mm by 100 mm) is placed horizontally on the thermal insulation device, and an absorber of the same size is positioned vertically 10 mm from the emitter edge. The positions of all components were carefully aligned according to the geometric configurations, with precise measurements performed to minimize alignment errors. Aluminum foil is applied to the back of the emitter to reduce radiative loss. In addition, seven concentric aluminum radiation shields are placed between the emitter and the vacuum chamber floor, supported by ceramic spacers (10 mm in height). The radiative heat loss coefficient is expressed as:

$$h_{\text{rad}} = \frac{4\varepsilon\sigma T_{\text{avg}}^3}{2N} \tag{S19}$$

where ε = 0.07 is the emissivity of the radiation shields, σ = 5.67 × 10^{-8} W m^{-2} K^{-4} is the Stefan–Boltzmann constant, and T_{avg} is the average temperature of the background and the emitter. The number of radiation shields is N = 7. For a conservative estimate, we assume that the temperature of the uppermost shield is equal to the background temperature, and thus the other radiation shields are invalidated. Considering the results of scenario 1 in vacuum, the average temperature T_{avg} of the background and the emitters in Systems I, II, and III is 323 K, 320K, and 314K, respectively. The corresponding radiative heat loss coefficients of emitters in vacuum are 0.04–0.27 W m^{-2} K^{-1}, 0.04–0.26 W m^{-2} K^{-1}, and 0.04–0.25 W m^{-2} K^{-1}. Similarly, in a typical indoor environment, the average temperature T_{avg} of the background and the emitters in Systems I, II, and III is 312 K, 310K, and 309K, respectively. The corresponding radiative heat loss coefficients of emitters in a typical indoor environment are 0.03–0.24 W m^{-2} K^{-1}, 0.03–0.24 W m^{-2} K^{-1}, and 0.03–0.23 W m^{-2} K^{-1}.

Next, to minimize the conductive loss of emitters, four polycarbonate (PC) pegs are used to support the emitter above the radiation shields. Each PC peg has a length of 100 mm and a diameter of 5 mm. The emitter is a square with a side length of 100 mm. The conduction coefficient of the emitters can be calculated using the following equation:

$$h_{\text{cond}} = \frac{k}{l} r \tag{S20}$$

where k and l denote the thermal conductivity and length of the PC pegs, respectively. The parameter r is the ratio between the cross-sectional area of the four PC pegs and the bottom surface area of the emitter. For the emitters in scenario 1, with r= 0.79% and k_{peg}= 0.5 W m^{-1} K^{-1}, the conduction coefficient $h_{\mathrm{cond-emi1}}$ is calculated to be 0.04 W m^{-2} K^{-1}. Therefore, in scenario 1, the parasitic heat transfer coefficient of emitters h_{emi1} is estimated to be in the range from 0.08 to 0.31 Wm^{-2} K^{-1} in vacuum, and from 7.07 to 7.28 Wm^{-2} K^{-1} in a typical indoor environment.

To minimize the radiative loss of absorbers, the backs of the absorbers are covered with aluminum foil, and the radiative heat loss coefficient is given by:

$$h_{\mathrm{rad-abs}} = 4\varepsilon\sigma T_{\mathrm{avg}}^{3} \tag{S21}$$

where ε= 0.07 is the emissivity of the aluminum foil. For scenario1 in vacuum, the average temperature T_{avg} of the background and absorbers is 301K. The corresponding radiative heat loss coefficient of absorbers is 0.43 W m^{-2} K^{-1}. Similarly, in a typical indoor environment, the average temperature T_{avg} of the background and absorbers is 297K. The corresponding radiative heat loss coefficient of absorbers is 0.42 W m^{-2} K^{-1}. In addition, a support plate (3 mm thick acrylic, with thermal conductivity k_{acrylic}= 0.19 W m^{-1} K^{-1}) is fixed to the back of the absorber. The cross-sectional area between the absorber and the support plate is 10 mm by 20 mm, resulting in $r =$ 2%. Then the conduction coefficient of the absorbers $h_{\mathrm{cond-abs1}}$ is calculated to be 1.27 W m^{-2} K^{-1}. Therefore, the parasitic heat transfer coefficient of absorbers h_{abs1} is estimated to be 1.7 Wm^{-2} K^{-1} in vacuum and 8.69 Wm^{-2} K^{-1} in a typical indoor environment.

In scenario 2, an emitter (30 mm by 30 mm) is placed horizontally on the thermal insulation device, and a parallel absorber of the same size is placed 30 mm above it. Four PC pegs are also used to support the emitter above the radiation shields. The parameter r is recalculated to be 8.72% for emitters and 3.33% for absorbers. Similarly, we calculate the parasitic heat transfer coefficients for scenario 2 using Supplementary Equation 8. The parasitic heat transfer coefficient of emitters h_{emi2} is estimated to be in the range from 0.47 to 0.72 Wm^{-2} K^{-1} in vacuum and from 7.47 to 7.67 Wm^{-2} K^{-1} in a typical indoor environment. The parasitic heat transfer coefficient of absorbers h_{abs2} is estimated to be 2.54 Wm^{-2} K^{-1} in vacuum and 9.53 Wm^{-2} K^{-1} in a typical indoor environment. The obtained parasitic heat transfer coefficients for different scenarios are used to calculate the radiative heat transfer efficiency and power consumption shown in Figs. 2 and 3.

Supplementary Note 5: Angle-resolved thermal emission spectrum measurement (ATESM)

Angle-resolved thermal emission spectrums (as shown in Fig. 2b,c of the main text) were performed using a customized ATESM system (Supplementary Fig. 18). This system includes a Fourier transform infrared spectrometer (FTIR) (VERTEX 80v, Bruker) coupled with a thermal emission collection setup. The emitter was mounted on a sample stage (Linkam FTIR600) with temperature control and two rotating stages. Rotating stage 1 and rotating stage 2 are used to control zenith angle θ (from -70° ~ 70°) and azimuth angle φ (from 0° ~ 360°), respectively. The sample was heated to 150 °C to ensure a reasonable signal-to-noise ratio. The emission was collimated using an off-axis parabolic mirror and then sent into FTIR. An array of vertically-aligned carbon nanotubes (500 μm tall CNT on Si substrate, NanoLab) was used as a 'blackbody' reference sample in calculating the sample emissivity.

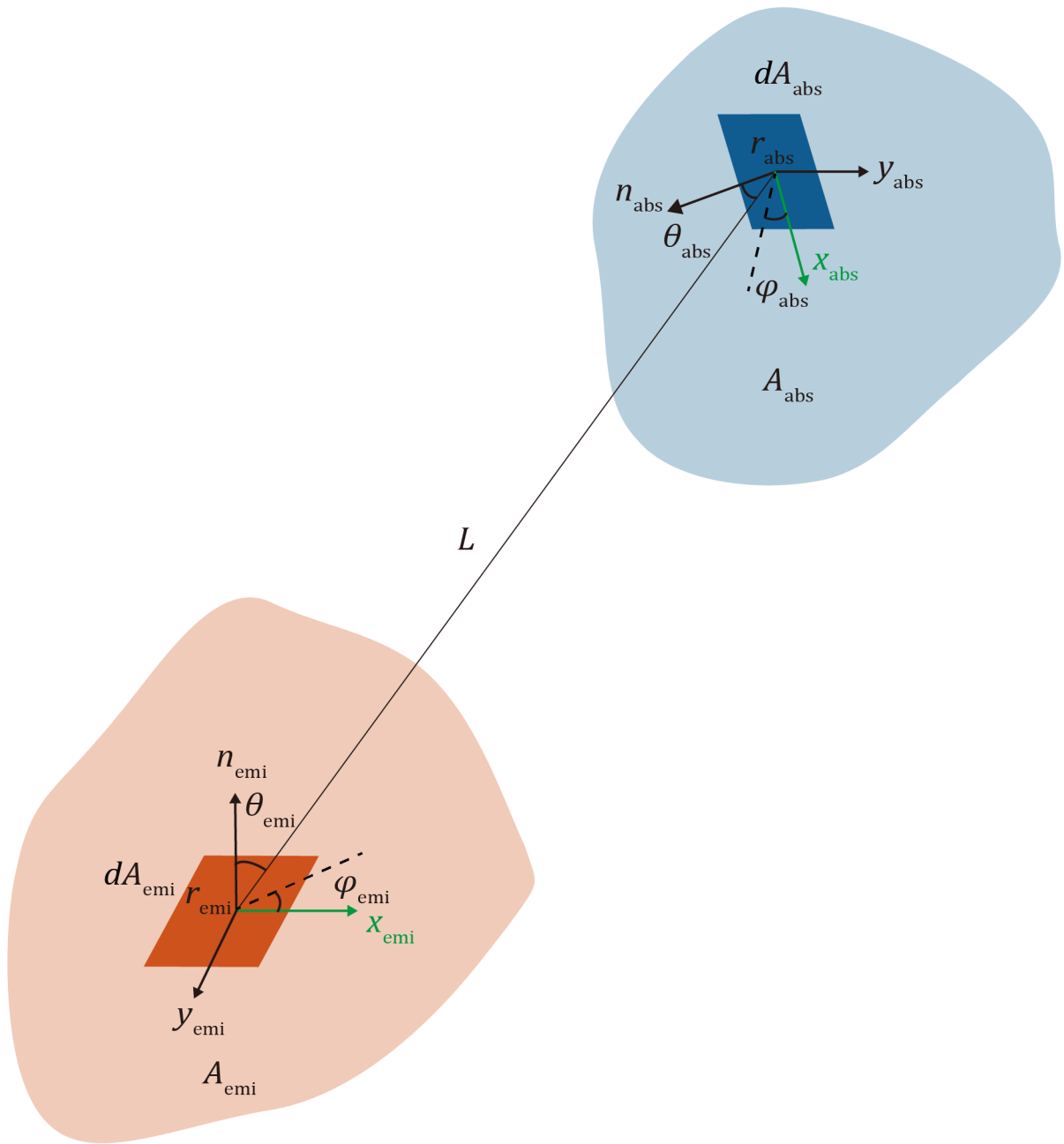

Supplementary Fig. 1. Schematic of emission angles between emitter $\boldsymbol{A}_{\mathbf{emi}}$ and absorber $\boldsymbol{A}_{\mathbf{abs}}$. The angles θ_{emi} and θ_{abs} are defined between their surface normal (n_{emi} and n_{abs}) and the line connecting dA_{emi} and dA_{abs} with length of L. The azimuth angle φ_{emi} is measured between the $\mathrm{x}_{\mathrm{emi}}$ axis on emitter A_{emi} and the projection of the line connecting dA_{emi} and dA_{abs} onto emitter A_{emi}. And the azimuth angle φ_{abs} is measured between the $\mathrm{x}_{\mathrm{abs}}$ axis on absorber A_{abs} and the projection of the line connecting dA_{emi} and dA_{abs} onto absorber A_{abs}. The vectors r_{emi} and r_{abs} denote the positions on A_{emi} and A_{abs}, respectively.

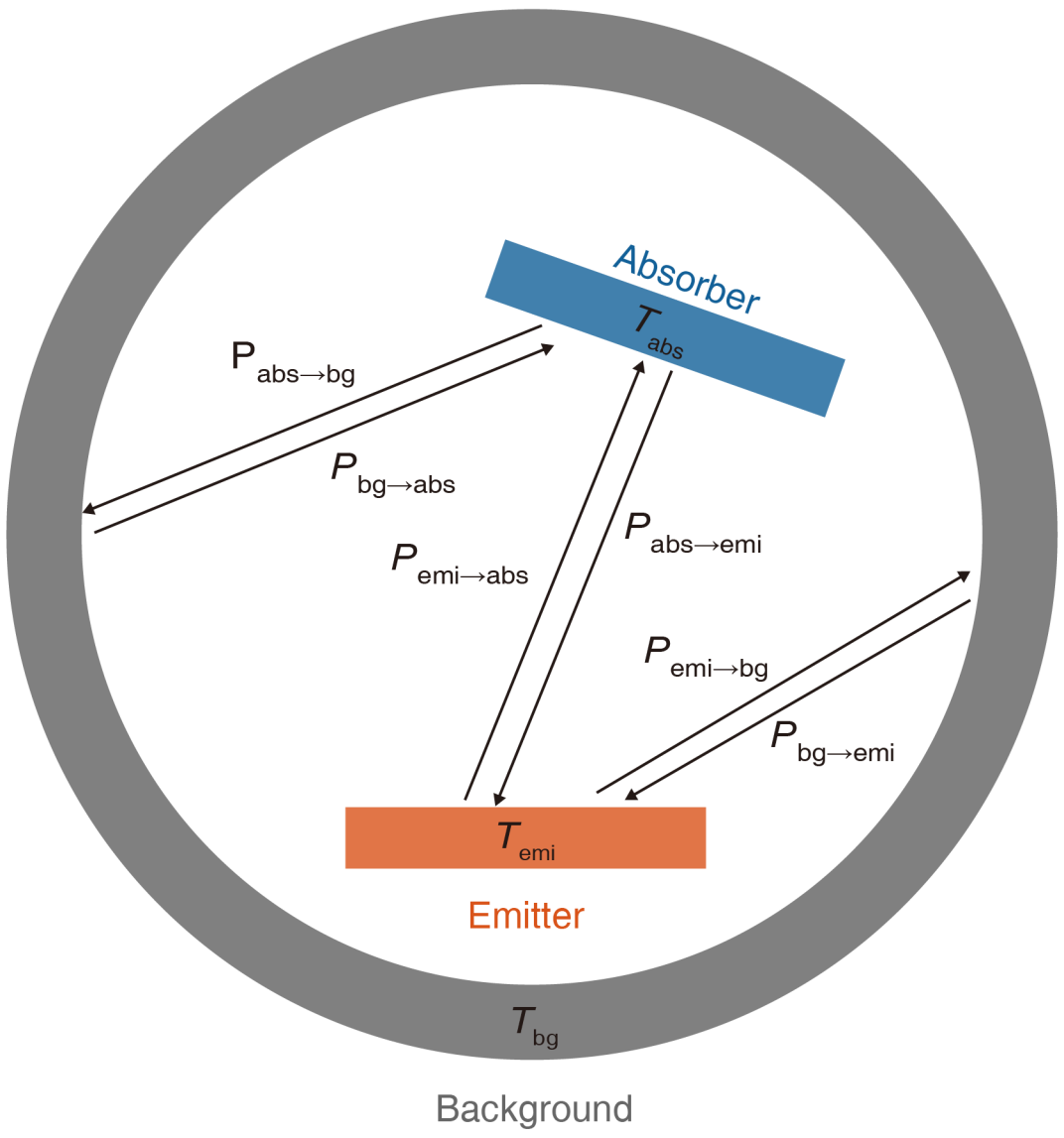

Supplementary Fig. 2. Radiative heat exchange among the emitter, absorber and background. T_{emi}, T_{abs} and T_{bg} are the temperatures of the emitter, absorber and background, respectively. $P_{\mathrm{bg}\rightarrow\mathrm{emi}}$ represents the radiation emitted by the background toward the emitter. $P_{\mathrm{bg}\rightarrow\mathrm{abs}}$ represents the radiation emitted by the background toward the absorber.

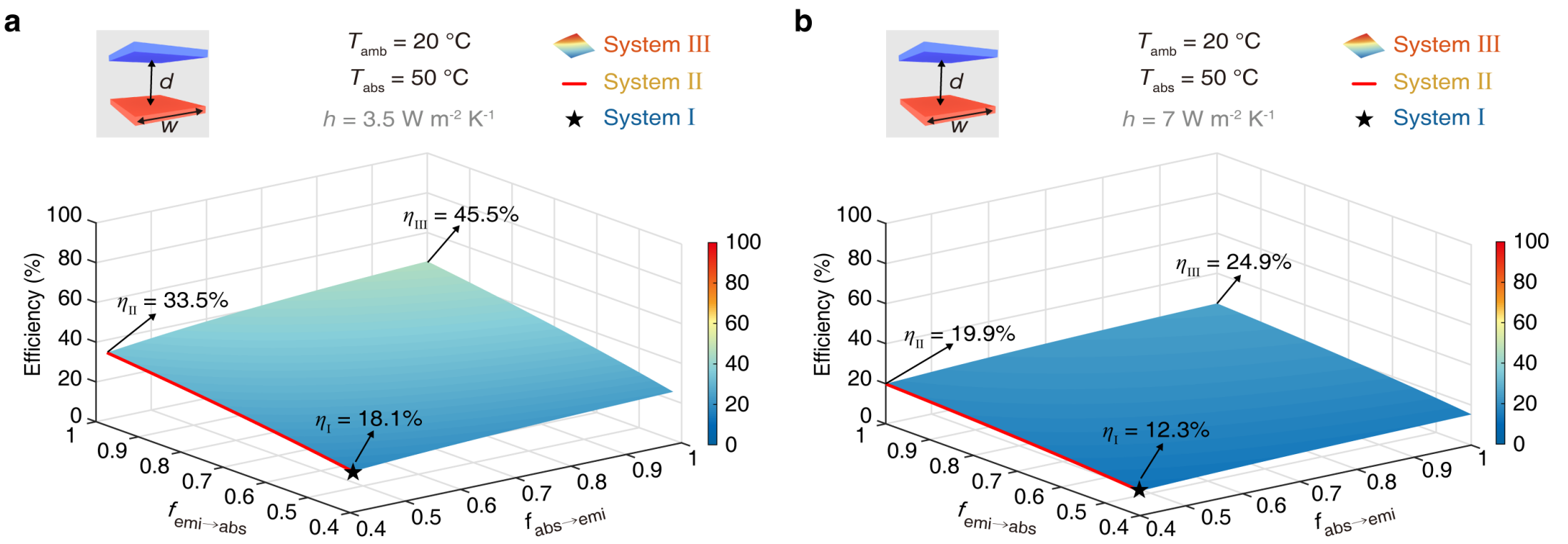

Supplementary Fig. 3. Radiative heat transfer efficiency η with different parasitic heat transfer coefficients. a,b, Radiative heat transfer efficiency η for the three systems as a function of transfer factors $f_{\mathrm{emi}\to\mathrm{abs}}$ and $f_{\mathrm{abs}\to\mathrm{emi}}$ under parasitic heat transfer coefficients h of 3.5 (**a**) and 7 (**b**) W m^{-2} K^{-1}, respectively. Although parasitic heat transfer losses significantly reduce efficiency, System III still can achieve the highest efficiency by optimizing the transfer factors. The ambient temperature and absorber temperature are set to T_{amb} = 20 °C and T_{abs} = 50 °C, respectively. Both the emitter and absorber have a width of w = 100 mm and are placed face-to-face with a separation distance of d = 50 mm.

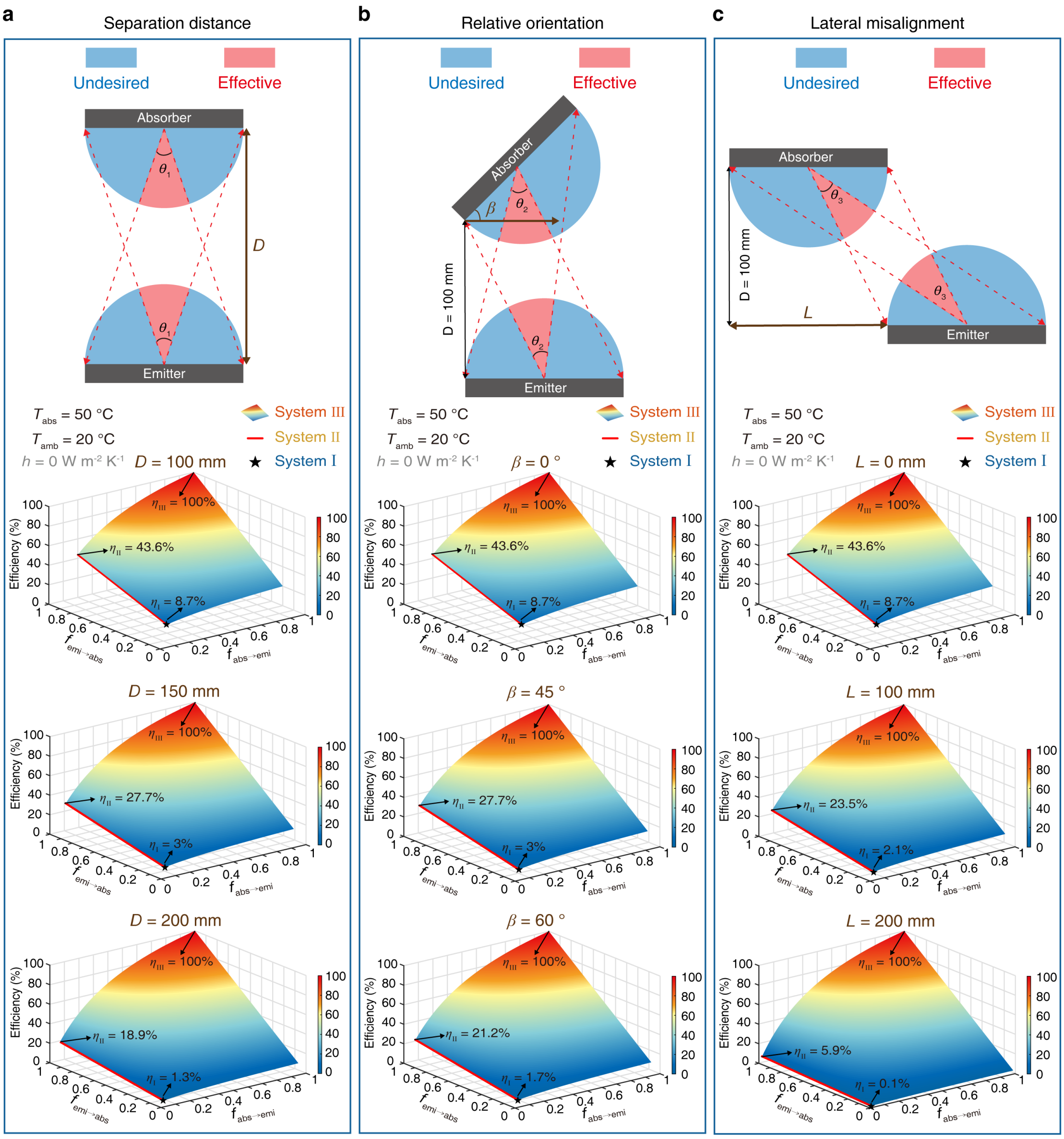

Supplementary Fig. 4. Theoretical radiative heat transfer efficiency η under different geometric configurations. a, Schematic of two surfaces (100 mm by 100 mm) placed face-to-face with a separation distance of D. The theoretical radiative heat transfer efficiencies of the three systems at different separation distances are shown below. **b,** Schematic of two surfaces (100 mm by 100 mm) oriented with a relative angle β at a fixed separation distance of 100 mm. The theoretical radiative heat transfer efficiencies of the three systems at different relative angles are shown below. **c,** Schematic of two surfaces (100 mm by 100 mm) with lateral misalignment L at a fixed separation distance of 100 mm. The theoretical radiative heat transfer efficiencies of the three systems at different lateral misalignments are shown below.

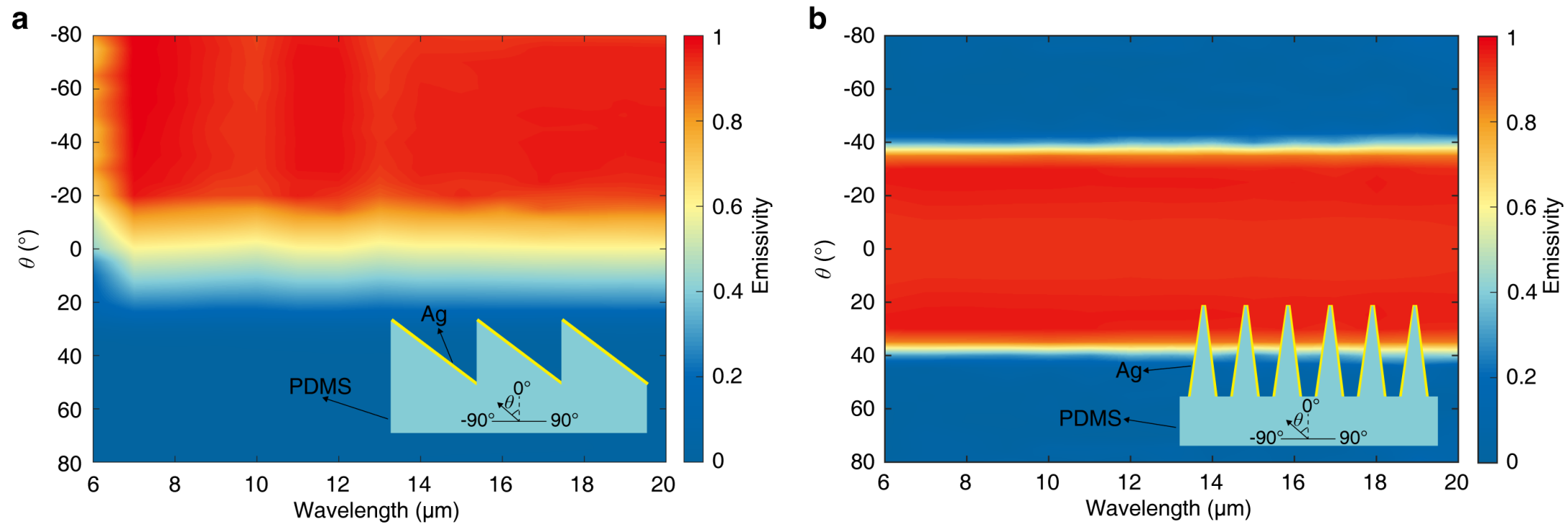

Supplementary Fig. 5. Simulated spectral angular emissivity of directional emitters in scenario 1 and 2. a, The directional emitter in scenario 1 exhibits angularly asymmetric emission arising from a sawtooth grating with broken in-plane mirror symmetry. In this design, the Ag layer on the slanted surfaces suppresses emission over the angular range of 0° to 80°, while emission from -80° to 0° is primarily contributed by the high-emissivity substrate (PDMS). **b,** The directional emitter exhibits high emissivity over the angular range of -40° to 40° based on a periodic conical structure. In this design, thermal emission from the bottom PDMS is collimated by Ag reflectors and redistributed into the designed angular range.

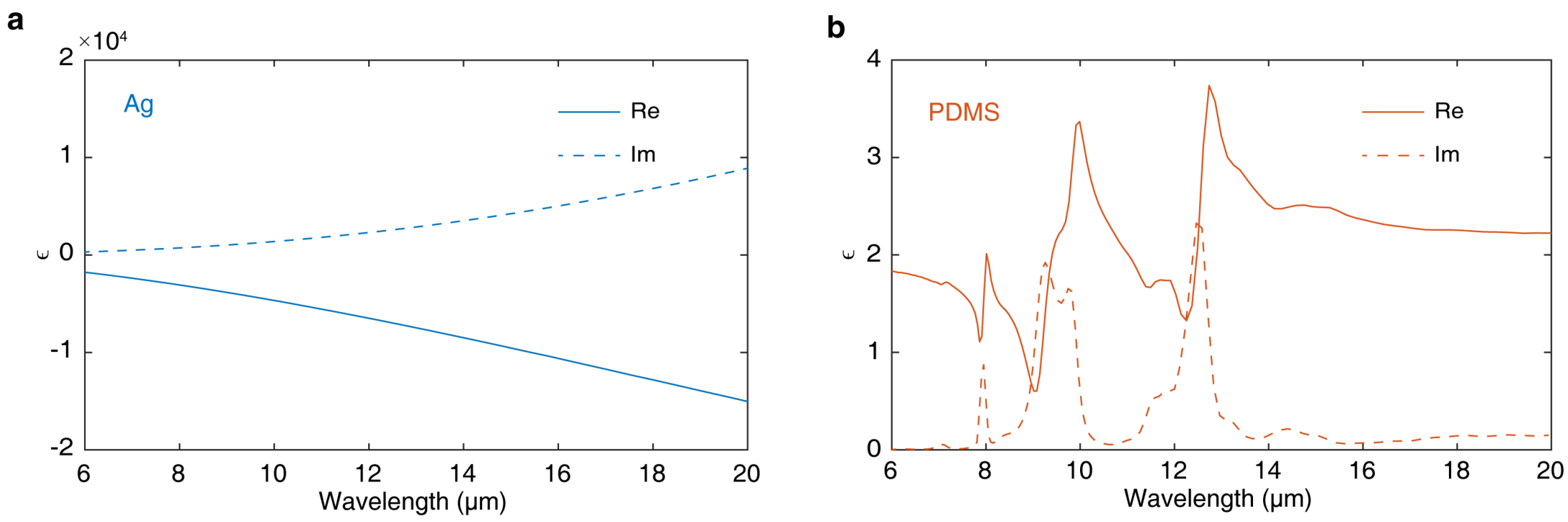

Supplementary Fig. 6. Permittivity of Ag and PDMS. a,b, Real (solid) and imaginary (dashed) parts of the permittivity of Ag (**a**) and PDMS (**b**) in the infrared wavelength range. Ag exhibits negligible absorptivity in the infrared range, whereas PDMS shows strong infrared absorption.

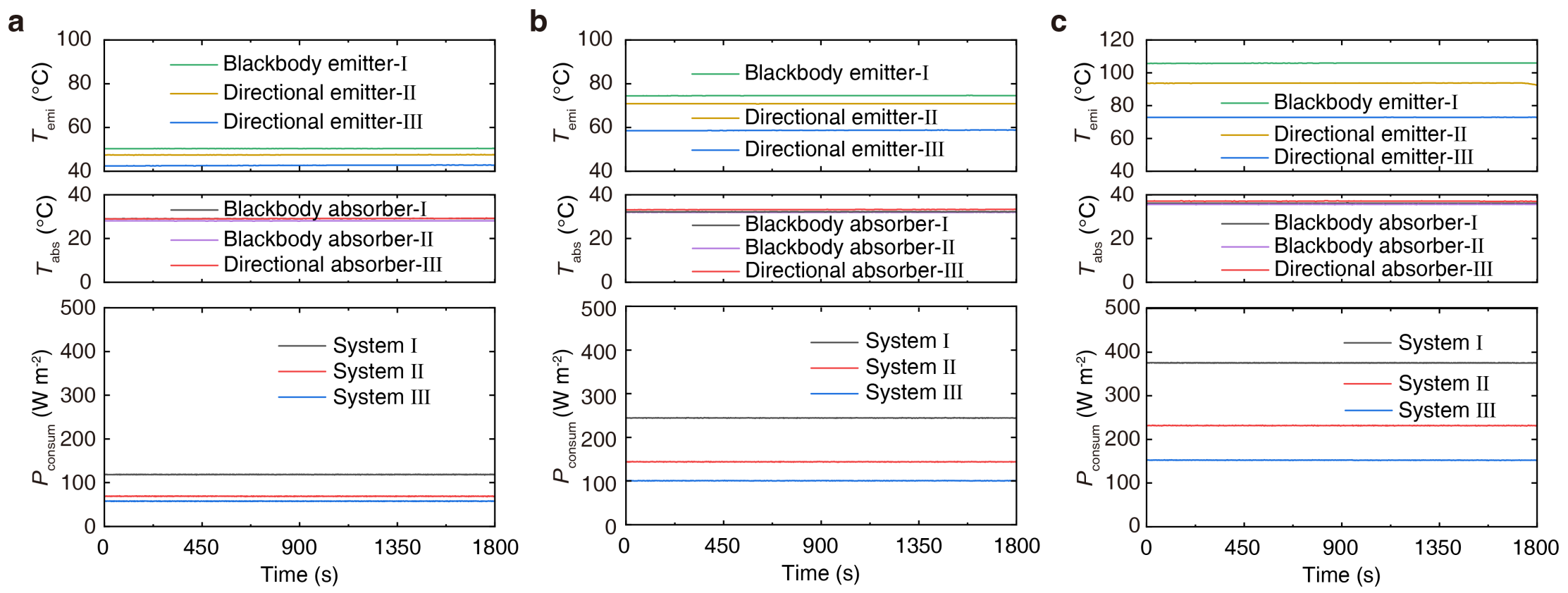

Supplementary Fig. 7. Equilibrium temperature and power consumption of scenario 1 in vacuum. a-c, The absorbers in the three systems are heated to approximately 28 ºC (**a**), 32 ºC (**b**), and 36 ºC (**c**), respectively. When the absorbers are maintained at the same temperature, System III exhibits the lowest emitter temperature T_{emi} and requires the least power consumption P_{consum}.

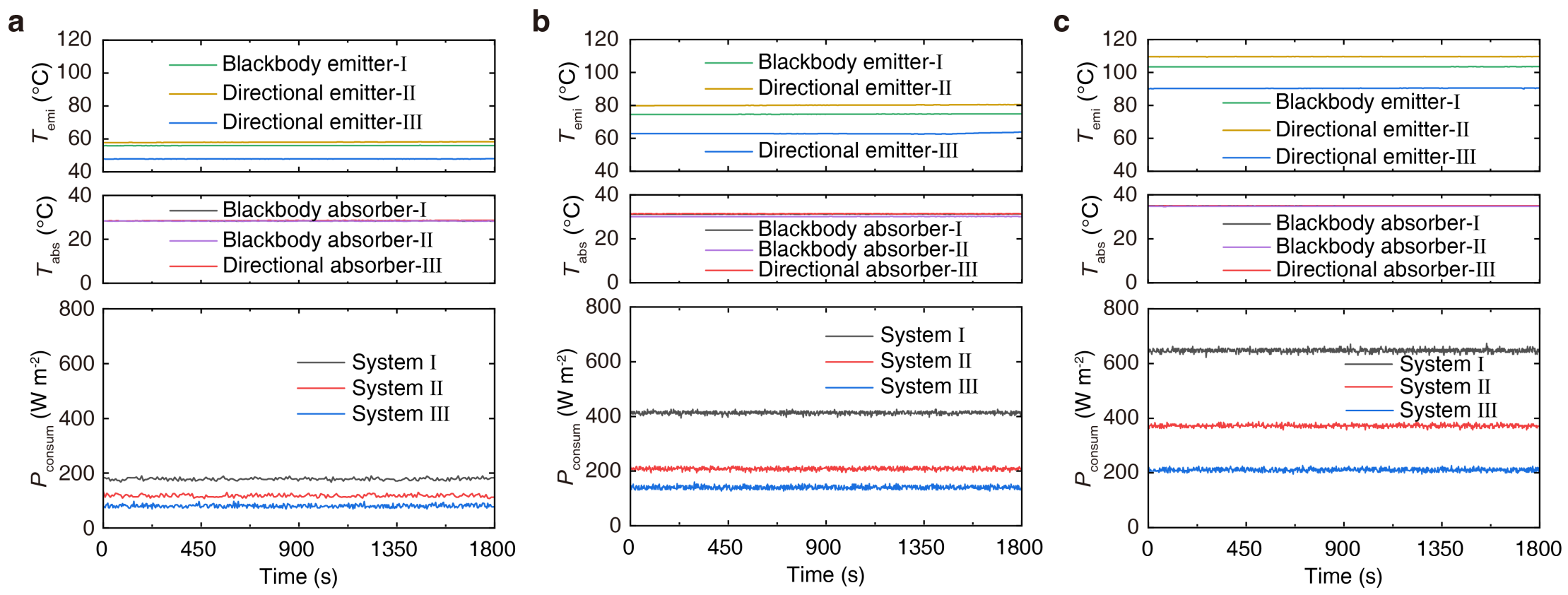

Supplementary Fig. 8. Equilibrium temperature and power consumption of scenario 2 in vacuum. a-c, The absorbers in the three systems are heated to approximately 28 ºC (**a**), 32 ºC (**b**), and 36 ºC (**c**), respectively. When the absorbers are maintained at the same temperature, System III exhibits the lowest emitter temperature T_{emi} and requires the least power consumption P_{consum}.

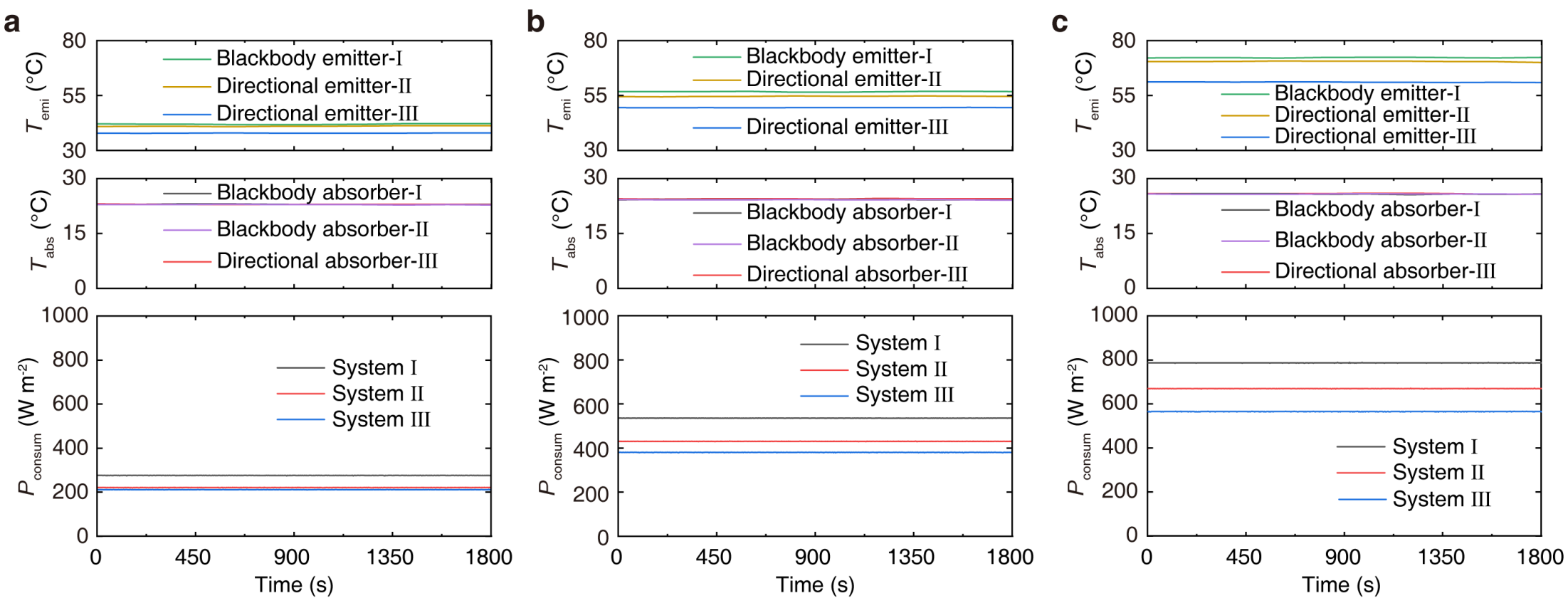

Supplementary Fig. 9. Equilibrium temperature and power consumption of scenario 1 in a typical indoor environment. a-c, The absorbers in the three systems are heated to approximately 23 ºC (**a**), 24.5 ºC (**b**), and 26 ºC (**c**), respectively. When the absorbers are maintained at the same temperature, System III exhibits the lowest emitter temperature T_{emi} and requires the least power consumption P_{consum}.

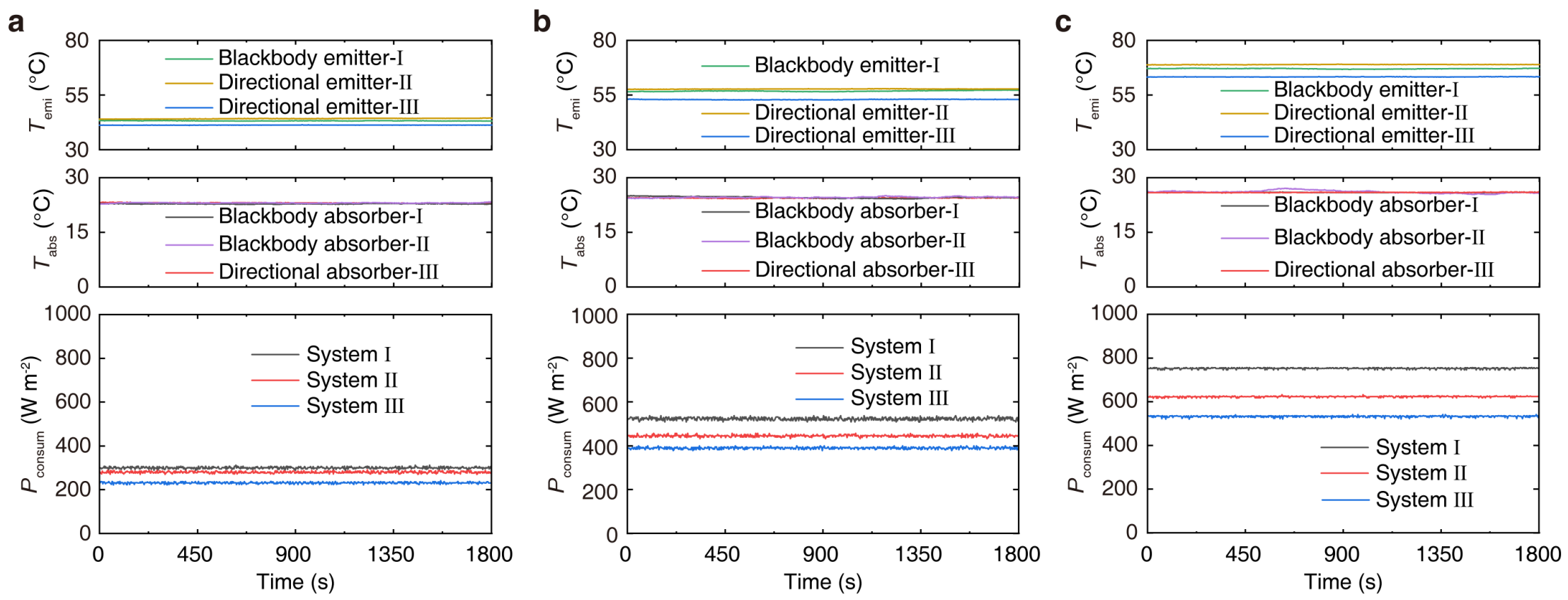

Supplementary Fig. 10. Equilibrium temperature and power consumption of scenario 2 in a typical indoor environment. a-c, The absorbers in the three systems are heated to approximately 23 ºC (**a**), 24.5 ºC (**b**), and 26 ºC (**c**), respectively. When the absorbers are maintained at the same temperature, System III exhibits the lowest emitter temperature T_{emi} and requires the least power consumption P_{consum}.

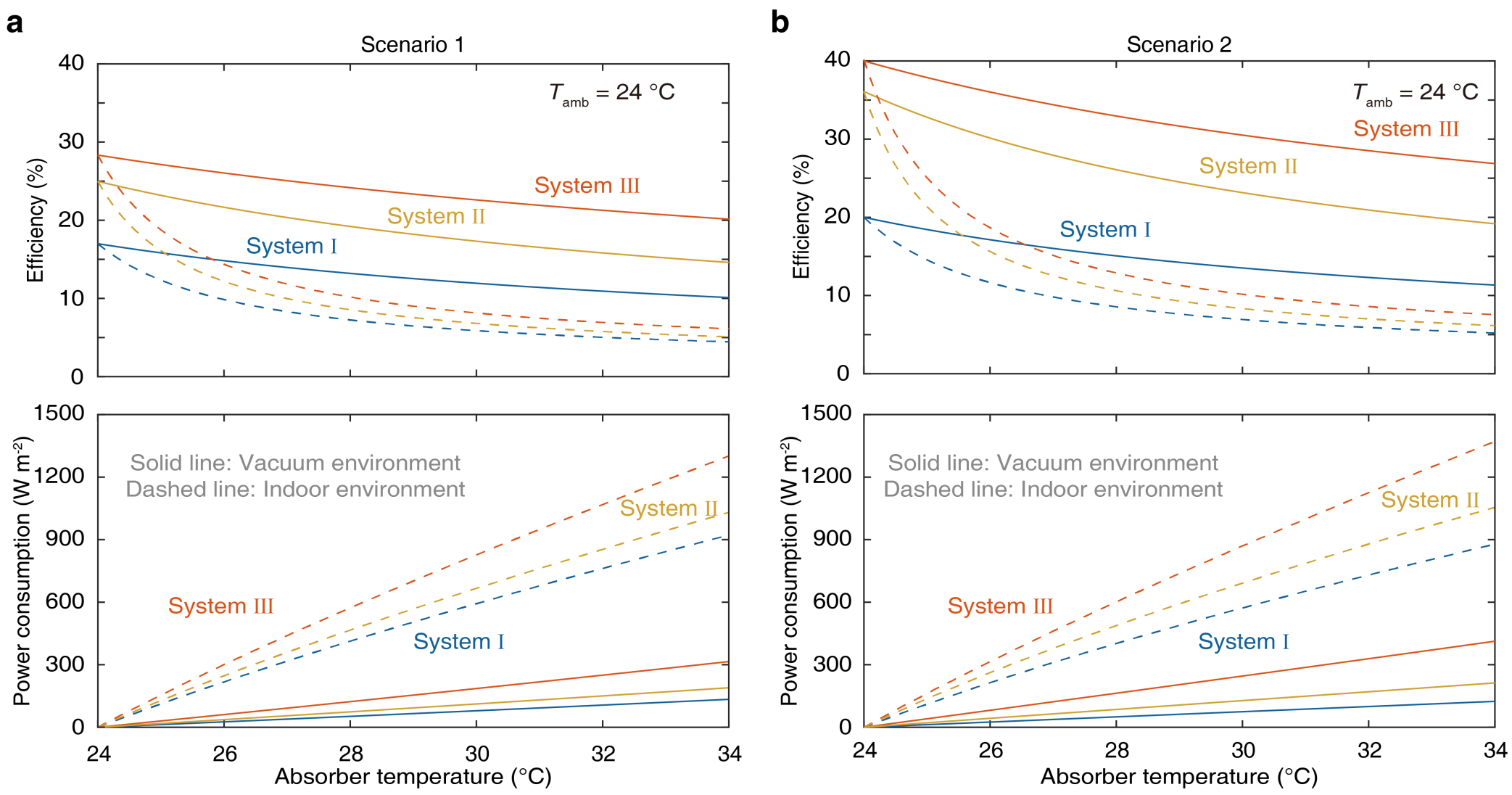

Supplementary Fig. 11. Simulated radiative heat transfer efficiency and power consumption in vacuum and typical indoor environments. a,b, Efficiency η and power consumption P_{consum} as functions of absorber temperature T_{abs} for three systems in scenario 1 (**a**) and scenario 2 (**b**). In both vacuum (solid line) and indoor environments (dashed line), System III consistently exhibits the highest efficiency and the lowest power consumption.

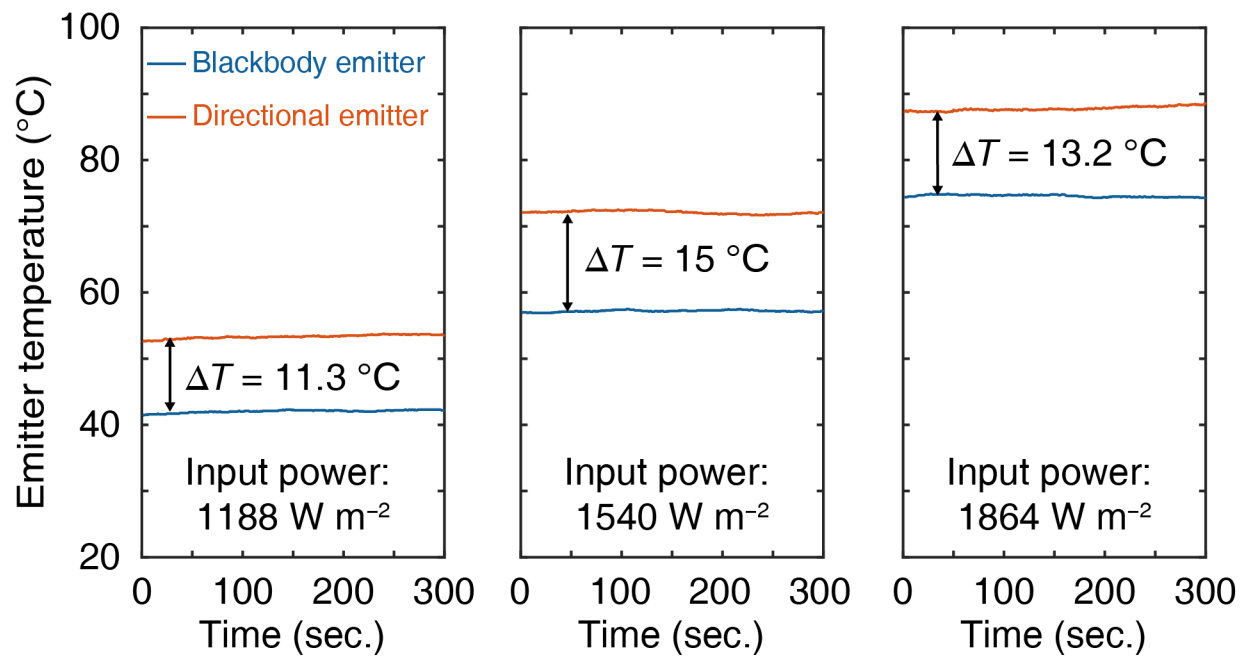

Supplementary Fig. 12. Equilibrium emitter temperature difference under the same input power in vehicle applications. At input powers of 1190, 1540, and 1871 W m^{-2} (with ambient temperature of T_{amb} = -20 °C in an environmental simulation chamber), the temperature of the directional emitter exceeds that of the blackbody emitter by approximately 11.3 °C, 15 °C, and 13.2 °C, respectively.

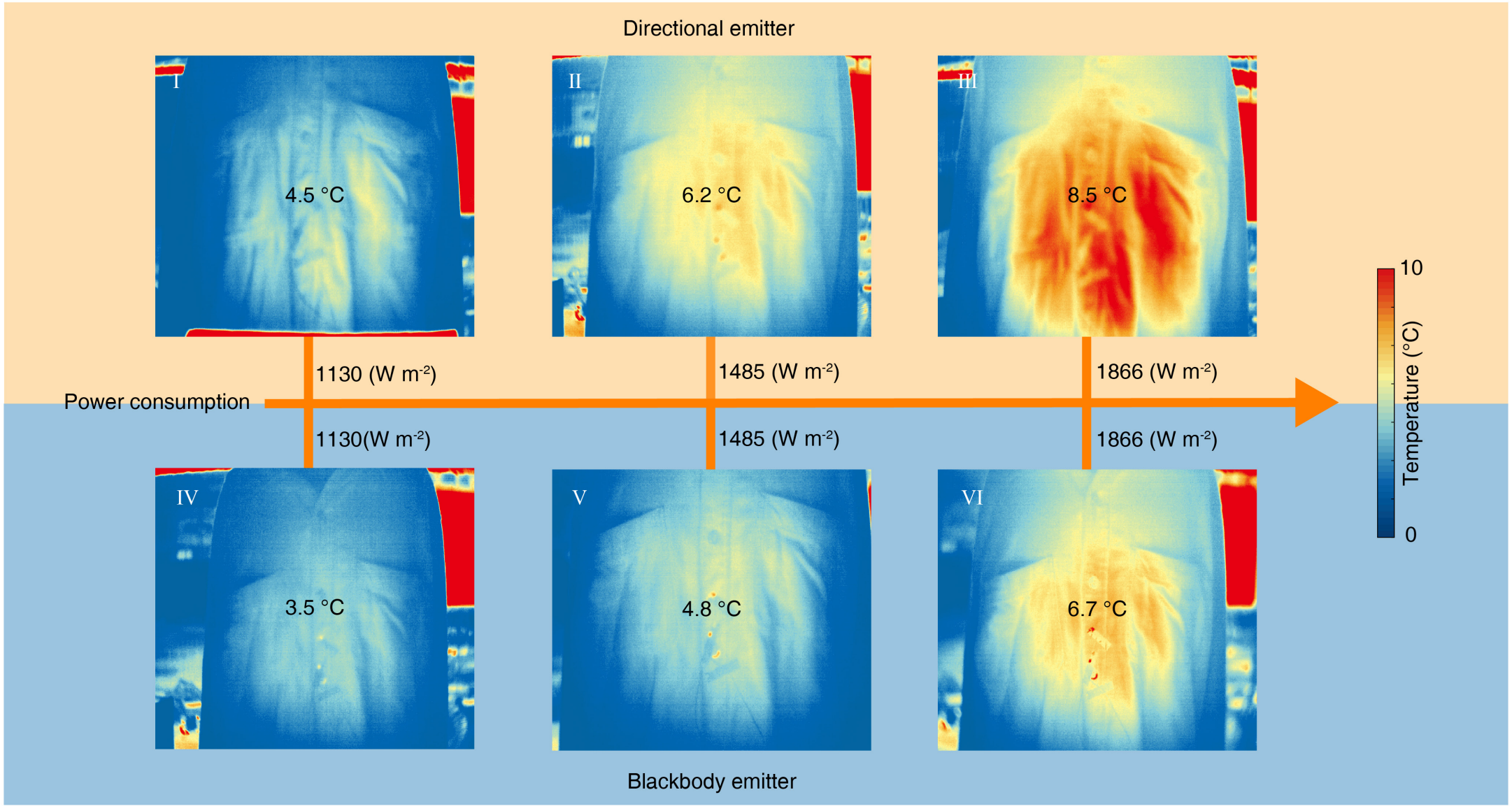

Supplementary Fig. 13. Infrared images of the mannequin at an ambient temperature of 0 °C. At power consumptions of 1130 (I and IV), 1485 (II and V), and 1866 (III and VI) W m^{-2}, the mannequin heated by the directional emitter is warmer than that heated by the blackbody emitter.

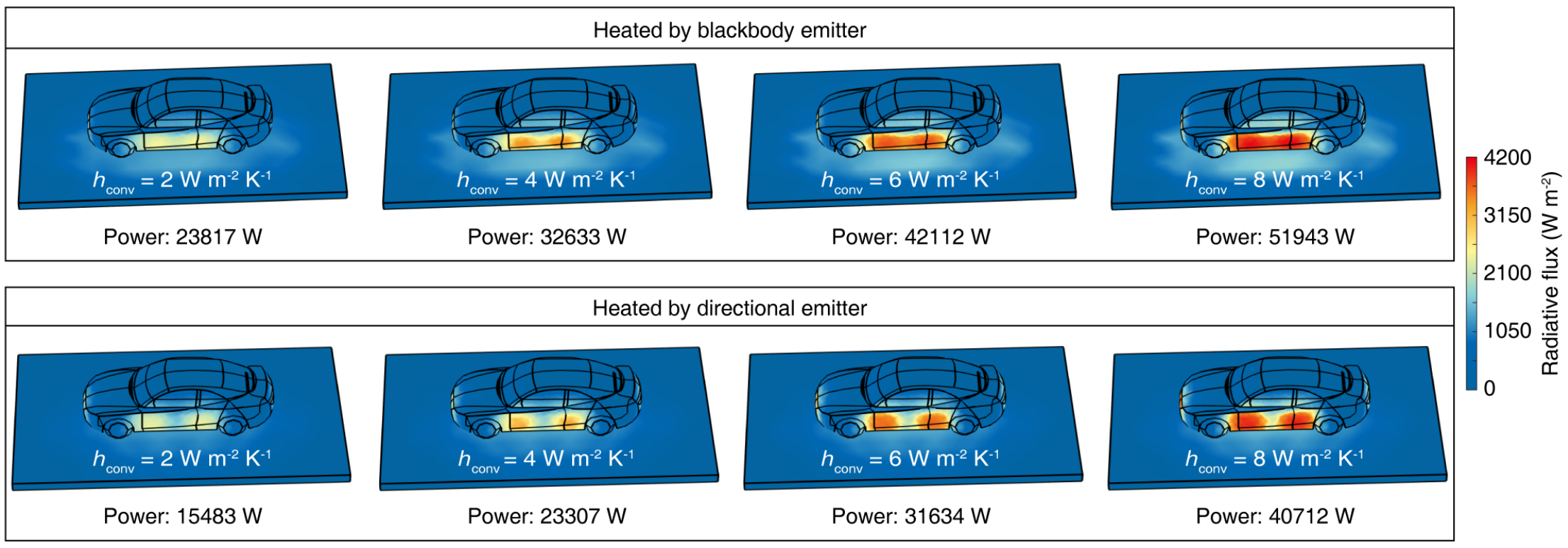

Supplementary Fig. 14. Simulated radiative flux distribution for car paint drying using blackbody emitters and directional emitters. Radiative flux distributions for achieving car paint temperatures of 60 °C, with the convection coefficient h_{conv} ranging from 2 to 8 W m^{-2} K^{-1} in indoor environments. The directional emitter primarily concentrates the radiative flux on the paint, leading to lower power consumption.

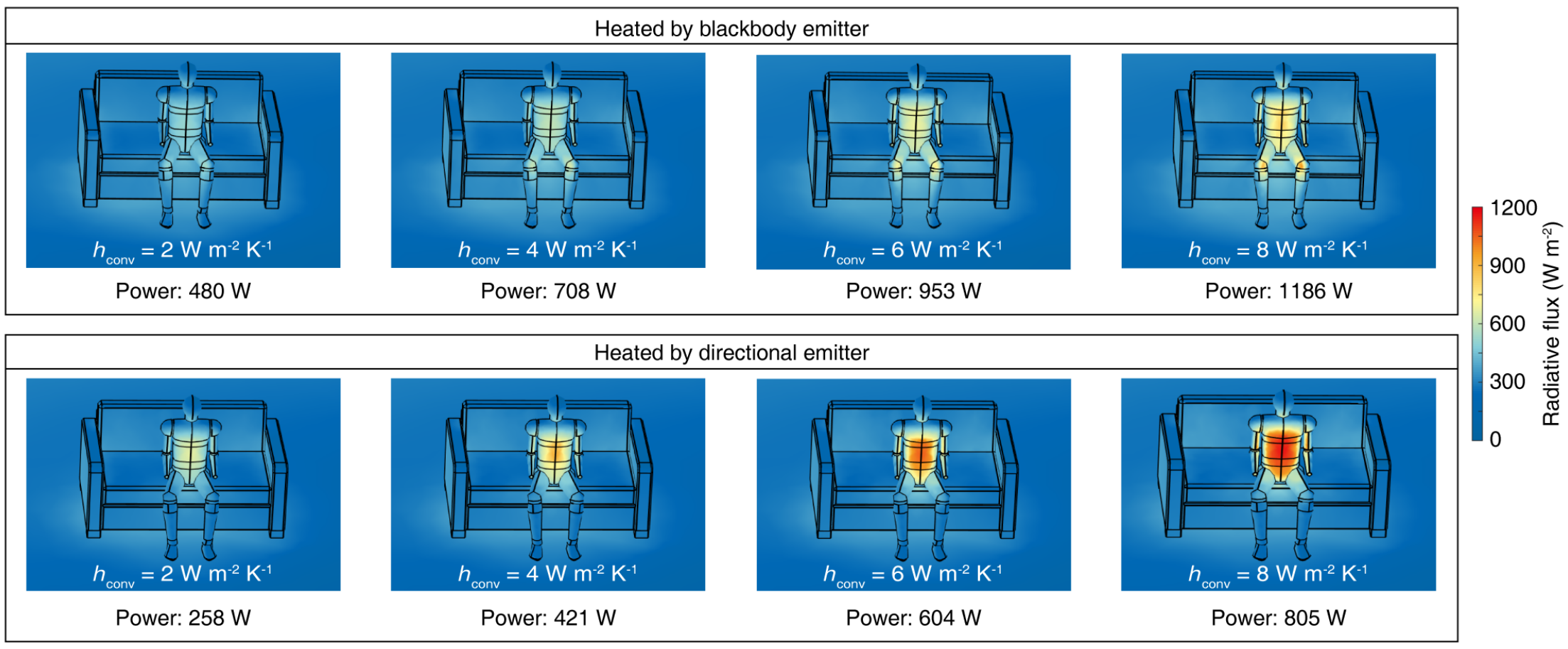

Supplementary Fig. 15. Simulated radiative flux distribution for human body heating using blackbody emitters and directional emitters. Radiative flux distributions required to raise the surface temperature of the mannequin by 3.5 °C, with the convection coefficient h_{conv} ranging from 2 to 8 W m^{-2} K^{-1} in indoor environments. The directional emitter primarily concentrates the radiative flux on the human body, leading to lower power consumption.

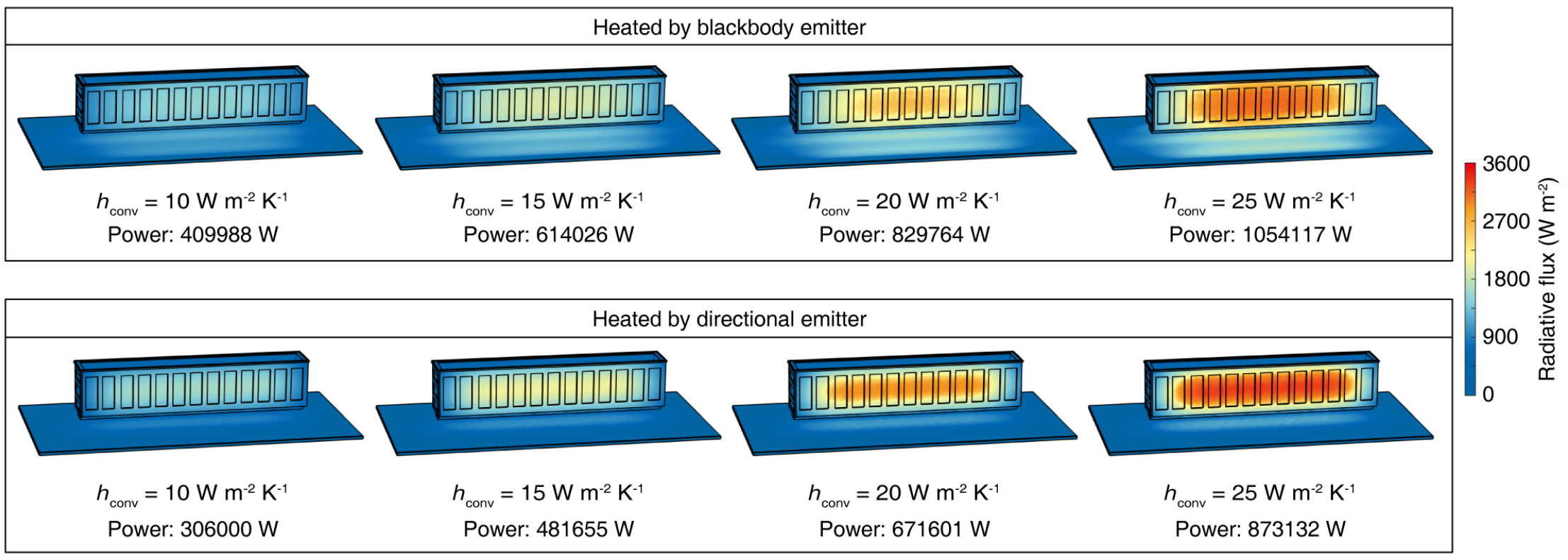

Supplementary Fig. 16. Simulated radiative flux distribution for rail car thawing using blackbody emitters and directional emitters. Radiative flux distributions for achieving rail car temperatures of 5 °C, with the convection coefficient h_{conv} ranging from 10 to 25 W m^{-2} K^{-1} in outdoor environments. The directional emitter primarily concentrates the radiative flux on the rail car, leading to lower power consumption.

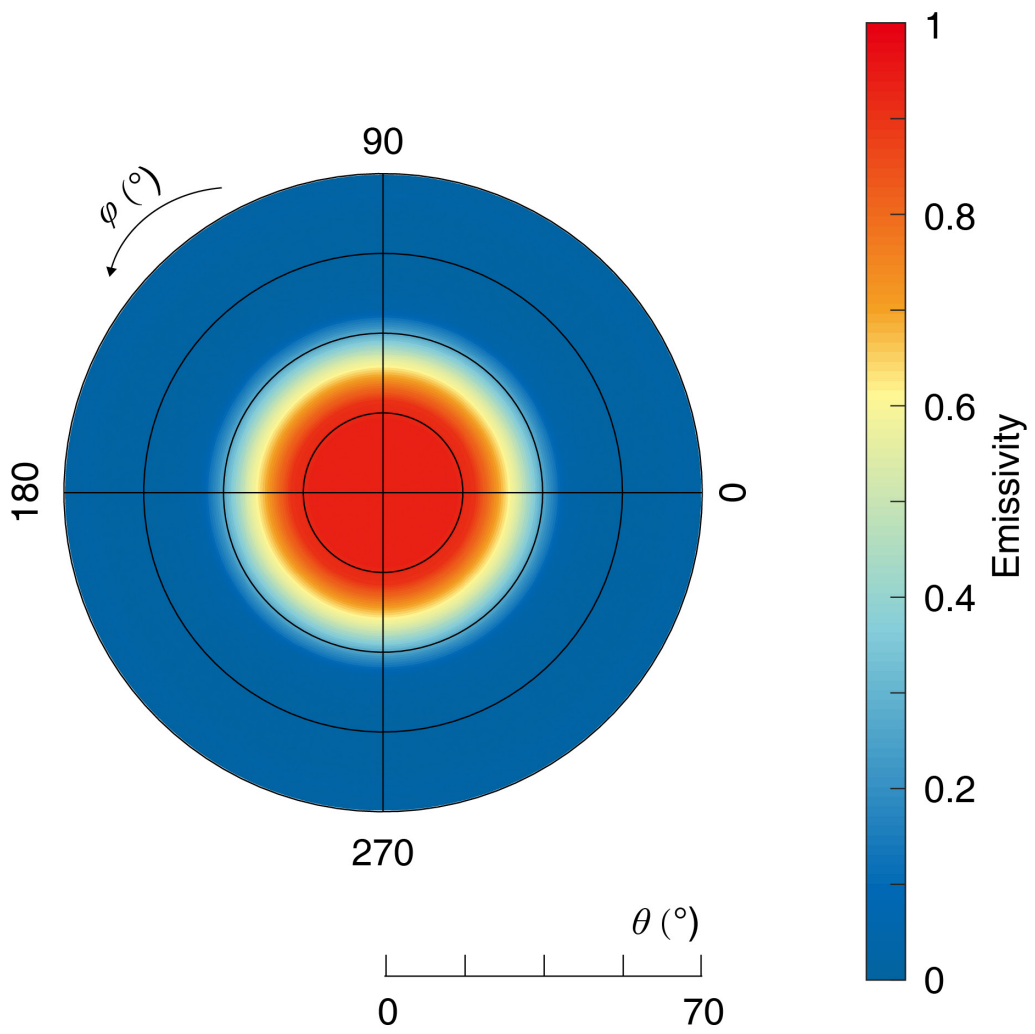

Supplementary Fig. 17. Simulated angular emissivity of the directional emitter for energy-saving evaluation. We redesigned the directional emitter shown in Fig. 2c with an emission zenith angle (θ) ranging from 0° to 25° for the energy-saving evaluation.

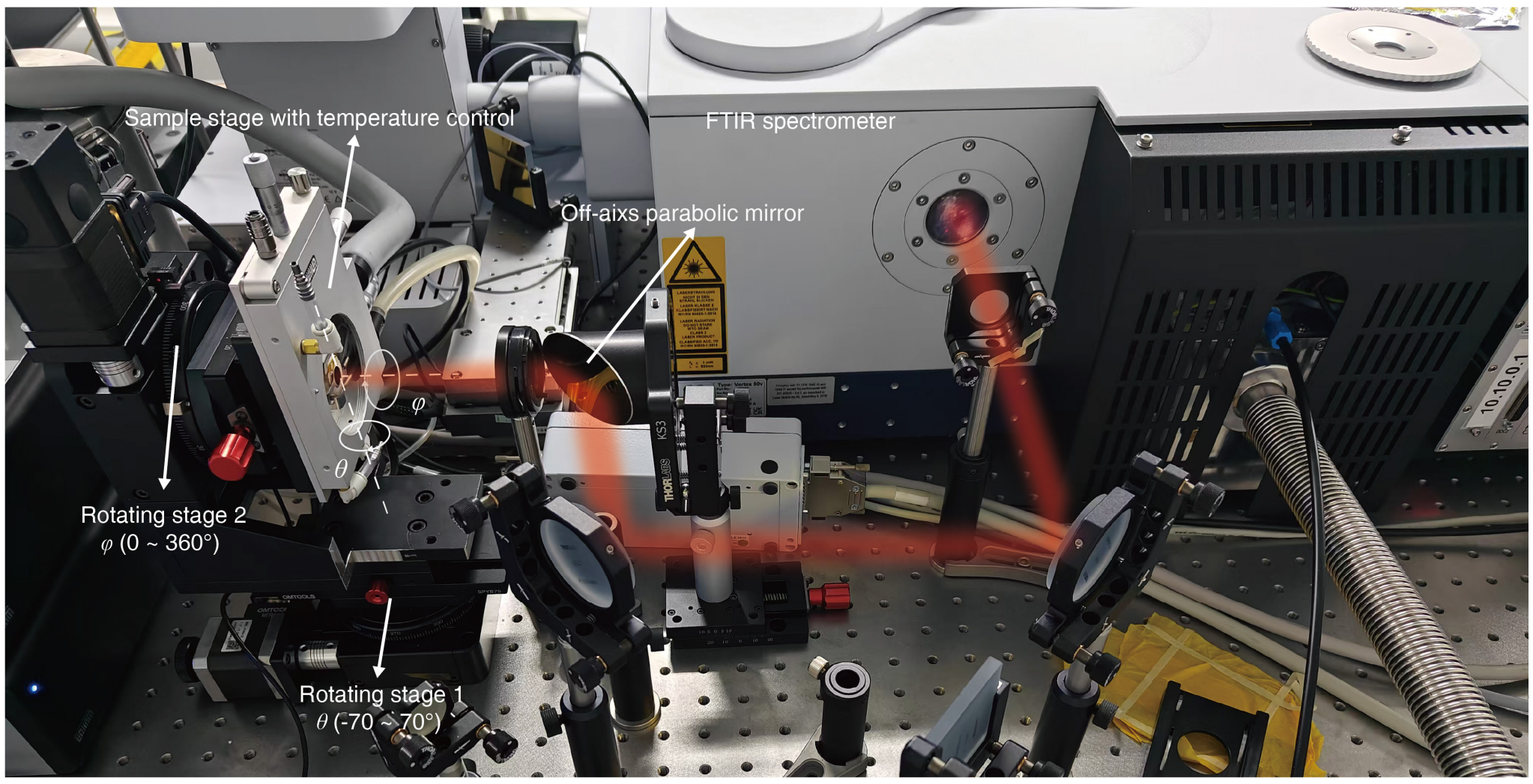

Supplementary Fig. 18. A customized angle-resolved thermal emission spectrum measurements (ATESM). This system includes a Fourier transform infrared spectrometer (FTIR) (VERTEX 80v, Bruker) coupled with a thermal emission collection setup. The emitter was mounted on a sample stage (Linkam FTIR600) with temperature control and two rotating stages. Rotating stage 1 and rotating stage 2 are used to control zenith angle θ (from -70° ~ 70°) and azimuth angle φ (from 0° ~ 360°), respectively. The sample was heated to 150 °C to ensure a reasonable signal-to-noise ratio. The emission was collimated using an off-axis parabolic mirror and then sent into FTIR.

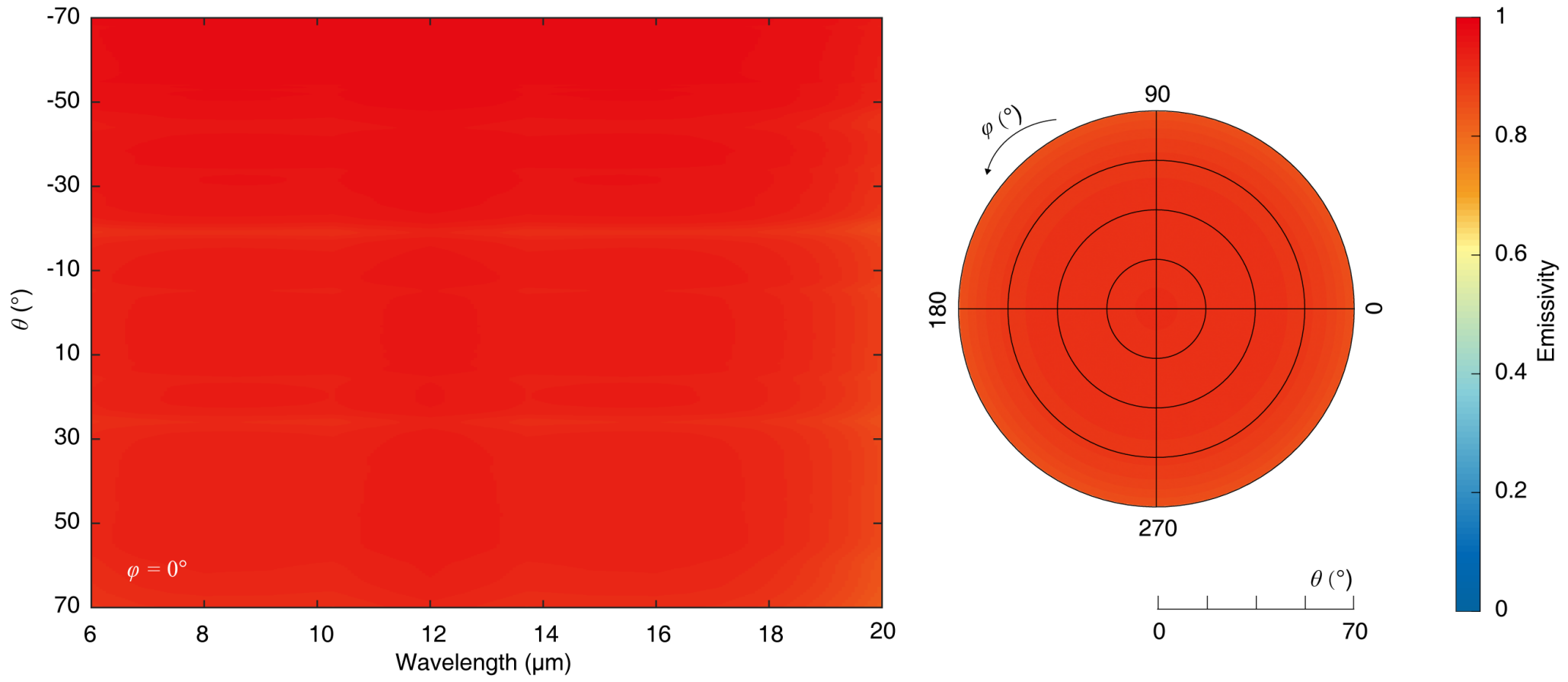

Supplementary Fig. 19. Measured spectral angular emissivity of the high emissivity paint. Spectral angular emissivity $\varepsilon(\lambda, \theta)$ (left) and angular emissivity $\varepsilon_{10\ \mu m}\ (\theta, \varphi)$ (right) at a wavelength of 10 μm varying with zenith (θ) and azimuth (φ) angles. The high emissivity paint exhibits broadband and omnidirectional thermal radiation with an emissivity of about 0.95.

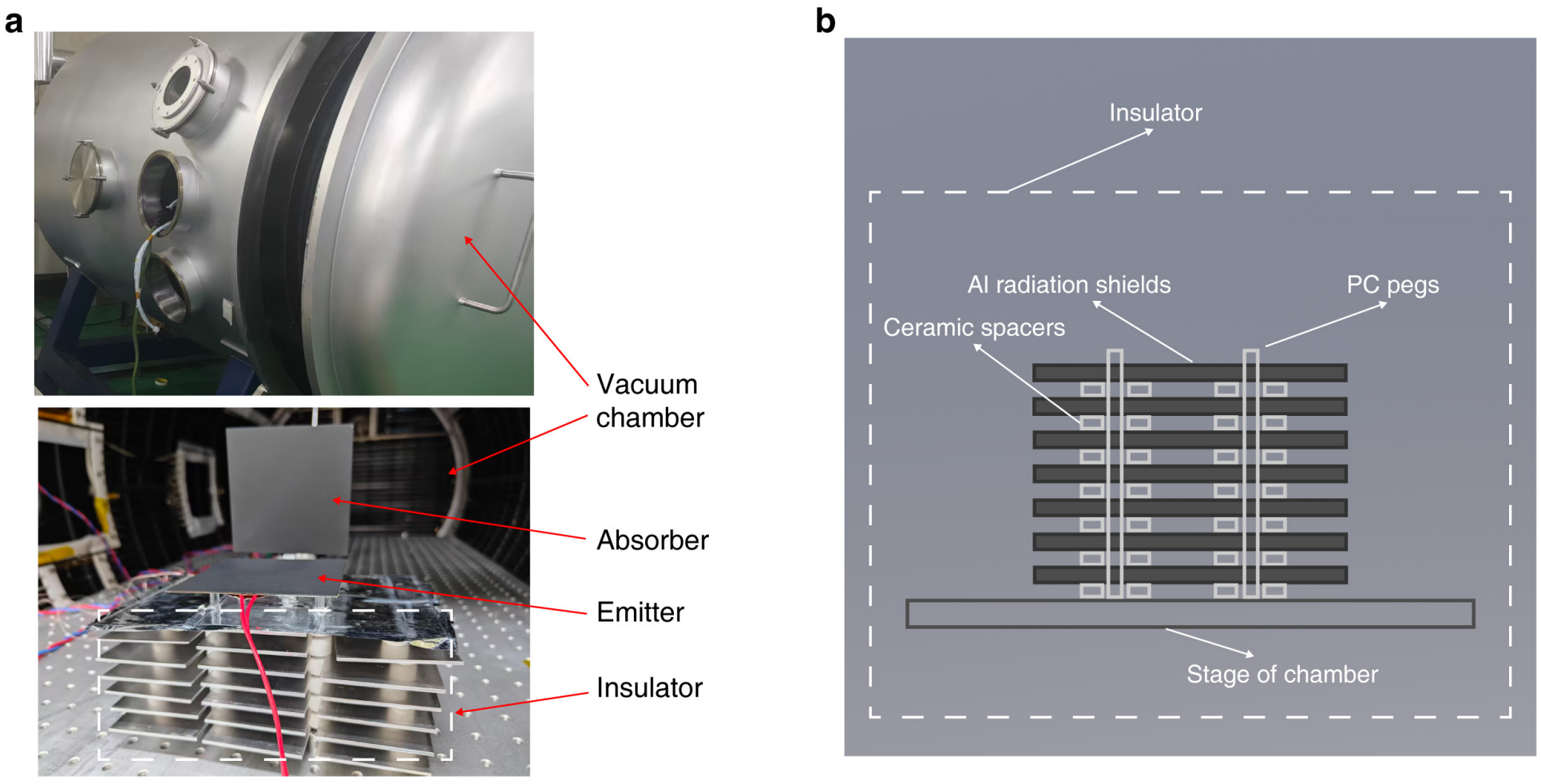

Supplementary Fig. 20. Photographs of the vacuum set-up and schematic of the thermal insulation device. a, Photographs of the vacuum chamber and experimental set-up. The vacuum level was kept at approximately 6×10^{-6} Torr. **b,** The thermal insulation device consists of four PC pegs and seven aluminum radiation shields, designed to minimize heat losses from radiation and conduction.

Supplementary Table 1| Practical scenario parameter for three radiative heating applications. Three practical scenarios are taken as examples: car paint drying[4], human body heating[5] and rail car thawing[6].

	Car paint drying	Human body heating	Rail car thawing
System schematic	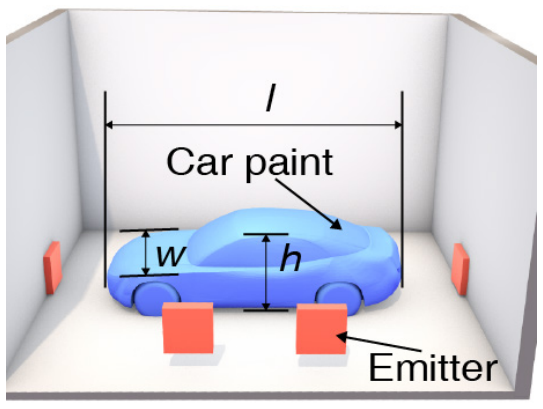	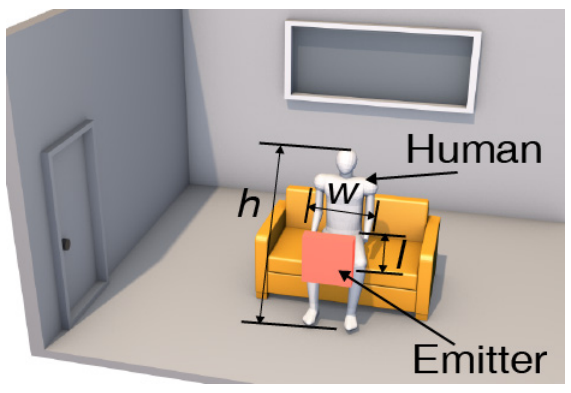	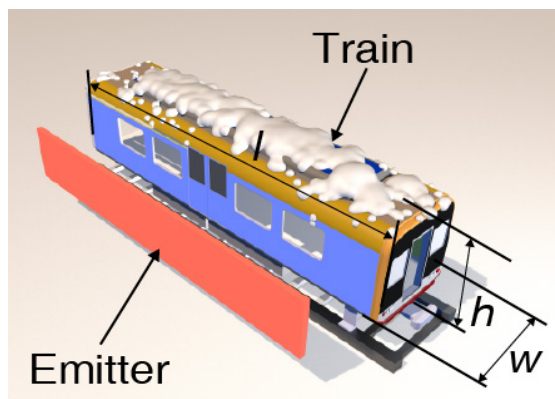
Ambient temperature (°C)	20	0	-20
Structure shape	length *l*, 4.58 m; width *w*, 1.82 m; height *h*, 1.40 m	length *l*, 0.54 m; width *w*, 0.68 m; height *h*, 1.35 m	length *l*, 15.43 m; width *w*, 2.45 m; height *h*, 3.16 m
Emitter shape	1 m*1 m	0.41 m*0.41 m	13.60 m*1.60 m
Convection coefficient ($W\ m^{-2}\ K^{-1}$)	2~8	2~8	10~25
Target area temperature (°C)	60	10	5

Supplementary References

1. Chen, Z., Zhu, L., Raman, A. & Fan, S. Radiative cooling to deep sub-freezing temperatures through a 24-h day–night cycle. *Nat. Commun.* **7**, 13729 (2016).
2. Bergman, L. T. *Introduction to Heat Transfer*. (Wiley, 2011).
3. Camci, M., Karakoyun, Y., Acikgoz, O. & Dalkilic, A. S. A comparative study on convective heat transfer in indoor applications. *Energy Build.* **242**, 110985 (2021).
4. Pendar, M.-R., Rodrigues, F., Páscoa, J. C. & Lima, R. Review of coating and curing processes: Evaluation in automotive industry. *Phys. Fluids* **34**, 101301 (2022).
5. Wang, H., Wang, J., Li, W. & Liang, S. Experimental study on a radiant leg warmer to improve thermal comfort of office workers in winter. *Build. Environ.* **207**, 108461 (2022).
6. Enerco Group, Inc. Rail Car Thawing. https://www.enerco.com/pages/rail-car-thawing
