# Supplementary material for: Directional thermal emission enables efficient energy savings
Source: Nat Commun. 2026 Jul 30;17:9261. doi: 10.1038/s41467-026-76043-z (PMC13526865; doi:10.1038/s41467-026-76043-z)
Supplement: Supplementary file 2 [file 41467_2026_76043_MOESM2_ESM.pdf]

nature portfolio

Peer Review File

Directional thermal emission enables efficient energy savings

Corresponding Author: Professor Wei Li

This file contains all reviewer reports in order by version, followed by all author rebuttals in order by version.

Version 0:

Reviewer comments:

Reviewer #1

(Remarks to the Author)
This work experimentally demonstrates that radiative heat transfer can be significantly enhanced when both the emitter and absorber are directionally engineered. The combination of experiments and calculations for realistic energy-saving scenarios provides valuable guidance for future research and practical energy-efficient thermal system design. The manuscript is therefore of remarkable significance, and I recommend publication in Nature Communications after minor revisions.

I would like the authors to clarify the following points:
1. In Fig. 1, the authors show that in the ideal limit the radiative heat-transfer efficiency can approach 100%. With realistic microstructural design of the emitter and absorber, what level of efficiency do the authors believe is practically attainable? It would be helpful to provide an estimate or briefly discuss the main limiting factors (e.g., impedance mismatch, parasitic loss, fabrication imperfections).

2. For scenario 2, the directional emitter exhibits noticeably higher emissivity in the simulations (Fig. S3) than in the experimental measurements (Fig. 2c). Could the authors comment on the possible physical or practical factors that may reduce the emissivity of the fabricated emitter in the desired angular range?

3. For Scenario 2, System II requires the highest emitter temperature to heat the absorber to the same target temperature as the other systems, as shown in both Fig. 2g and Fig. 3e. This does not seem to correspond well with the power consumption and efficiency curves in Fig. 2e and Fig. 3c. Could the authors elaborate on the physical mechanism responsible for this behavior? In addition, why does this trend differ from Scenario 1, where System I instead requires the highest emitter temperature, as shown in both Fig. 2f and Fig. 3d?

4. In Fig. 2f, the temperature difference between the emitters in Systems II and III is much larger than that between Systems I and II. However, in the realistic scenario (Fig. 3d), when T_abs=24 C and 27 C, the temperature difference between Systems II and III becomes very small, while the difference between Systems I and II remains noticeable. Could the authors clarify the physical origin of this discrepancy between the idealized and realistic cases?

5. In Fig. 4a, the mannequin and clothing appear positioned relatively low with respect to the emitter. The authors may wish to comment on the effective view factor or angular overlap between the directional emission lobe and the heated target, and clarify whether geometric mismatch could affect the measured performance.

6. In Fig. 3a, the caption states a separation of 30 mm between the parallel plates. From the photograph, the spacing appears visually larger. The authors may wish to confirm the actual separation distance and clarify the measurement definition.

7. The authors may wish to clarify that the analysis is dominated by mid-infrared thermal radiation and comment briefly on whether shorter-wavelength absorption plays any measurable role in their experiments.

8. In Supplementary Note 3, the thermal conductivity values appear to be given in units of $W \cdot m^{-2} \cdot K^{-1}$, whereas thermal conductivity should have units of $W \cdot m^{-1} \cdot K^{-1}$.

Reviewer #2

(Remarks to the Author)
Thermal radiation is a fundamental heat transfer mechanism in many energy-related systems, including building heating, industrial processes, transportation, and human thermal comfort. Despite its importance, radiative heat transfer in practical systems is commonly treated as omnidirectional, which inevitably leads to energy dissipation toward non-target regions and constrains overall system efficiency. While recent advances in photonic and thermal design have enabled increasing control over the directionality of thermal emission, the system-level implications of directional radiative heat transfer, particularly in realistic operating environments, remain insufficiently explored. This manuscript investigates directional radiative heat transfer through a combination of a generalized theoretical framework and experimental demonstrations conducted under vacuum, indoor, and application-oriented conditions. By introducing the transfer factor, the work provides a unified description of directional radiative coupling that extends beyond conventional view-factor-based approaches. The accompanying experiments illustrate how directional thermal emission can influence energy consumption in several representative heating scenarios across different environments. Overall, the study addresses a topic of clear relevance to thermal management and energy-related applications and contributes a systematic analysis of directional radiative heat transfer that bridges theory and experiment. Therefore, it is a timely and exciting research that warrant consideration by Nature Comm.

Major issues:
In the theoretical analysis presented in Figs. 1–3, the manuscript emphasizes the advantages of System III, in which both the emitter and the absorber exhibit directional characteristics and are directionally matched to achieve the highest radiative heat transfer efficiency. However, the application-oriented demonstrations, particularly the vehicle heating experiment in Fig. 4, appear to correspond more closely to the configuration of System II, since the human body and clothing, acting as absorbers in this scenario, generally behave as broadband, nearly blackbody absorbers without directional selectivity. A similar issue may also arise in the practical scenarios illustrated in Fig. 5, where the absorbers do not appear to exhibit directional absorption characteristics. The authors should clarify this apparent disconnection between the theoretical framework and the application demonstrations, and clearly define the scope within which the advantages of System III are expected to be realized.

Detailed technical issues:
1)Page 4: In Fig. 1, the theoretical analysis adopts a configuration consisting of two planar surfaces with fixed dimensions and a defined separation distance. While the geometric parameters are specified, the dependence of the transfer factor f on geometric variables such as separation distance, relative orientation, and lateral misalignment is not explicitly discussed. Since geometric coupling fundamentally governs radiative exchange, this dependence should be clarified. A supplementary discussion or parametric analysis showing how variations in distance or relative angle influence f and the efficiency η is necessary. Even a limited example would help readers understand the geometric robustness and applicability of the proposed framework.
2)The theoretical comparison in Fig. 1 assumes a transfer factor of approximately 0.42 for blackbody-based systems, corresponding to a specific close-range configuration. In practical applications, however, the transfer factor can be significantly smaller due to larger separations or more complex geometries. The manuscript should clarify how the efficiency advantage of System III evolves as f decreases. Under a fixed heating requirement, it is not clear whether the magnitude of power savings remains substantial when f becomes much smaller than the illustrative value used in Fig. 1. This issue is also relevant to the experimental configurations in Figs. 2 and 3, where emitter and absorber are placed in close proximity. It should be clarified whether short separation distances are an inherent requirement of the proposed approach. A brief parametric trend showing efficiency enhancement as a function of f would clearly define the applicable range of the directional strategy, particularly for far-field or large-scale scenarios.
3)Line 92 – 104 on page 3: The manuscript emphasizes that the transfer factor formulation generalizes conventional view-factor analysis and applies to systems with directional emission. However, the practical implementation of this formulation for complex or asymmetric geometries is not explained. The manuscript should clarify how the transfer factor would be evaluated in such cases. Providing guidance on computational methods, complexity, or expected accuracy would help readers assess whether the framework can be applied to realistic engineering systems.
4)Page 6: Figure 2 presents vacuum experiments demonstrating directional radiative heat transfer. While the schematic is clear, the experimental description lacks sufficient detail for reproducibility. Photographs of the vacuum setup and a concise description of key parameters should be provided in the Supporting Information. These should include sample mounting, alignment tolerances, temperature sensor placement, vacuum level stability, and radiation shielding configuration. Such information is necessary for evaluating parasitic effects and reproducing the reported results.
5)Page 8: In the non-vacuum indoor experiments shown in Fig. 3, convection and conduction contribute to heat transfer in addition to radiation. Although parasitic heat transfer coefficients are included in the model, the relative contributions of radiation, convection, and conduction are not quantified. A quantitative decomposition based on the existing model parameters should be provided. This analysis is necessary to demonstrate whether directional radiation remains the dominant mechanism under realistic indoor conditions.
6)Page 10: The vehicle heating demonstration in Fig. 4 compares a directional emitter with a blackbody emitter. While this comparison isolates the effect of directional radiation, its engineering relevance requires clarification. In practical vehicles, cabin heating is primarily achieved through convective air heating, typically using heat-pump-based HVAC systems. Moreover, the radiative heating configuration illustrated beneath Fig. 4b represents a heating mode that is uncommon in real vehicle applications, which limits the interpretability of the chosen reference. The manuscript should clearly define the intended role of directional radiative heating in vehicles, for example whether it is proposed as a primary heating mechanism

or as a supplemental method for localized thermal comfort. The limitations of comparing with a blackbody emitter, rather than with widely used HVAC systems, should also be discussed.
7)In the human heating scenario presented in Fig. 5, the local radiative heat flux incident on the target region reaches values on the order of 1000 W m^{-2}. While the manuscript correctly emphasizes the localized nature of this heating, such flux levels may raise concerns regarding thermal comfort or safety if interpreted without context. How these flux values relate to accepted thermal comfort or exposure thresholds, and how to specify the spatial extent and typical exposure duration associated with the reported conditions?
8)While the Discussion highlights the conceptual advantages of directional radiative heat transfer, the manuscript would benefit from a brief perspective on manufacturing and deployment considerations. Directional emitters often rely on photonic or metamaterial structures whose scalability, cost, and long-term stability may influence real-world adoption. A brief discussion should be added on large-area fabrication, integration with existing heating systems, and durability under thermal cycling. This perspective is necessary to place the work in a realistic engineering context.

In summary, the work provides a systematic analysis of directional radiative heat transfer, linking theoretical modeling with experimental validation in realistic environments. The results help clarify the conditions under which directional emission can improve energy efficiency, which is of interest to the thermal management and energy community.

Reviewer #3

(Remarks to the Author)
I co-reviewed this manuscript with one of the reviewers who provided the listed reports. This is part of the Nature Communications initiative to facilitate training in peer review and to provide appropriate recognition for Early Career Researchers who co-review manuscripts.

Reviewer #4

(Remarks to the Author)
This manuscript presents a well-designed and experimentally validated study on directional radiative heat transfer. The introduction of the transfer factor provides a meaningful extension beyond the conventional view factor framework, and the combination of theoretical analysis and practical demonstrations convincingly shows substantial energy savings in targeted heating applications. The reduction in emitter temperature together with decreased power consumption further highlights the practical significance of the work.

However, I encourage the authors to clarify the thermodynamic interpretation of the reported efficiency enhancement. In many demonstrated cases, the improvement primarily results from suppressing radiative losses to undesired surroundings and concentrating heat onto a specific target. In this sense, the mechanism may be better interpreted as spatial heat relocation rather than an increase in net heat extraction capability. A brief discussion distinguishing directional heat focusing from true net heat removal to an external reservoir would improve conceptual clarity and position the work more precisely within the broader context of radiative thermal management.

Reviewer #5

(Remarks to the Author)
In this work, the authors demonstrate that directional thermal emission can significantly enhance radiative heat transfer efficiency by reducing unwanted radiative losses into undesired directions. They introduce a transfer-factor framework to quantify efficiency in directional emitter–absorber systems and show that perfectly matched angular emission and absorption can theoretically yield 100% efficiency. Experimental demonstrations confirm substantial power savings compared with blackbody emitters, and several application scenarios illustrate potential practical benefits. Overall, the work is interesting and could merit publication in Nature Communications. However, several important issues must be addressed and clarified before it can be considered for publication.
Major issues:

The physical mechanism underlying the thermal radiation directivity is not described in sufficient depth. Directionality is treated largely as an imposed or effective property through angular emissivity profiles, without a clear electromagnetic explanation of how the proposed structures generate directional emission. Key wave-physics mechanisms—such as interference effects, resonant mode selection, momentum filtering, or anisotropic dispersion—are not rigorously analyzed, which limits physical insight. This aspect should constitute the core of the manuscript.
In addition, the materials and emitter structuration are mainly characterized through effective emissivity or absorptivity. The roles of frequency-dependent dielectric functions, material losses, dispersion, temperature dependence, and fabrication tolerances are not systematically discussed. As a result, the constraints imposed by real materials on the achievable directionality and efficiency remain unclear.
Finally, foundational theoretical works on directional thermal emission and thermal antennas appears to be underrepresented or absent from the bibliography. Given the close conceptual connection to the present study, this omission weakens both the completeness of the references and the physical positioning of the work.
Minor issues:

Additional limitations include the highly idealized nature of the 100% efficiency claim, the simplified treatment of convection and conduction, and the strong dependence of the reported performance on geometry and alignment. Consequently, the estimated energy savings are likely optimistic under realistic operating conditions.

Version 1:

Reviewer comments:

Reviewer #1

(Remarks to the Author)
The authors have thoroughly addressed all of my comments and concerns. The revised manuscript and Supplementary Information have substantially improved the quality, completeness, and rigor of the work. In my view, the paper is now ready for publication in Nature Communications.

Reviewer #2

(Remarks to the Author)

Reviewer #3

(Remarks to the Author)
I co-reviewed this manuscript with one of the reviewers who provided the listed reports. This is part of the Nature Communications initiative to facilitate training in peer review and to provide appropriate recognition for Early Career Researchers who co-review manuscripts.

Reviewer #4

(Remarks to the Author)
The revised version is ready for publication.

Reviewer #5

(Remarks to the Author)
Overall the authors have significantly improved their manuscript. However several aspects of the physics should be clarified further to strengthen the manuscript.

-The manuscript sometimes gives the impression that the efficiency enhancement is fundamentally new physics, whereas much of the gain originates from geometric/angular confinement of radiation. It would help to emphasize more explicitly that the key novelty is the extension of radiative transfer
theory to directional emitters/absorbers and its practical implementation.

-The distinction between “directional thermal emission” and “true thermal antennas” is not sufficiently discussed. The manuscript cites ENZ thermal antennas and broadband directional emitters, but the physical connection between these previous works and the present macroscopic radiative-transfer framework should be explained more clearly.

-Equation (2) is central to the paper, but the derivation and physical meaning of the transfer factor deserve more intuition. A short discussion explaining its physical meaning would greatly improve readability.

-The practical applications section is convincing from an engineering perspective, but some claims could be toned down slightly. For example, the link to “global carbon neutrality and sustainability” appears several times and may sound broader than what is directly demonstrated experimentally.
Scientifically, the manuscript would also benefit from citing and discussing several missing papers. The following missing references are important and should be cited because they cover several key advances that are directly related to the present work but are not adequately represented in the current bibliography, the first explicit formulation of a “true thermal antenna” based on hyperbolic metamaterials (Barbillon et al.) and thermally tunable broadband directional emitters (Hwang et al.),

- J. S. Hwang, J. Xu, and A. P. Raman, “Thermally Tunable Angular Selectivity of Broadband Directional Thermal Emission,” Nano Lett. 25, 8064 (2025).
- G. Barbillon, J.-P. Hugonin, S.-A. Biehs, and P. Ben-Abdallah, “True thermal antenna with hyperbolic metamaterials,” Opt. Express 25, 23356 (2017).

Open Access This Peer Review File is licensed under a Creative Commons Attribution 4.0 International License, which permits use, sharing, adaptation, distribution and reproduction in any medium or format, as long as you give appropriate credit to the original author(s) and the source, provide a link to the Creative Commons license, and indicate if changes were made.
In cases where reviewers are anonymous, credit should be given to 'Anonymous Referee' and the source.
The images or other third party material in this Peer Review File are included in the article's Creative Commons license, unless indicated otherwise in a credit line to the material. If material is not included in the article's Creative Commons license and your intended use is not permitted by statutory regulation or exceeds the permitted use, you will need to obtain permission directly from the copyright holder.
To view a copy of this license, visit https://creativecommons.org/licenses/by/4.0/

Response to the Reviewers' Comments on the manuscript entitled 'Directional thermal emission enables efficient energy savings' submitted to *Nature Communications*

We extend our sincere appreciation to the editor and all reviewers for their careful review of our manuscript and positive feedback on our manuscript. We are grateful for their recognition of the significance and excitement of our work, and for offering constructive suggestions to further enhance the quality of our work. In the response letter below, we address each comment of the reviewers, and append a revised manuscript with all revisions highlighted in yellow (highlighted figure captions correspond to updated figures). With these revisions implemented, we believe that our manuscript in alignment with the standards for publication in *Nature Communications*.

Response to Reviewer #1:

Reviewer #1:

This work experimentally demonstrates that radiative heat transfer can be significantly enhanced when both the emitter and absorber are directionally engineered. The combination of experiments and calculations for realistic energy-saving scenarios provides valuable guidance for future research and practical energy-efficient thermal system design. The manuscript is therefore of remarkable significance, and I recommend publication in Nature Communications after minor revisions.

I would like the authors to clarify the following points:

Response: We express our gratitude to the reviewer for carefully reviewing our manuscript and for recognizing the potential impact and remarkable significance of our work, as well as supporting its publication in *Nature Communications*. We also appreciate the reviewer's insightful comments and suggestions which we address point by point as below.

Comment #1: In Fig. 1, the authors show that in the ideal limit the radiative heat-transfer efficiency can approach 100%. With realistic microstructural design of the emitter and absorber, what level of efficiency do the authors believe is practically attainable? It would be helpful to provide an estimate or briefly discuss the main limiting factors (e.g., impedance mismatch, parasitic loss, fabrication imperfections).

Response: We appreciate the reviewer's comment. The 100% efficiency limit shown in Fig. 1 of the main text represents an idealized upper bound that highlights the fundamental advantage of directional radiative heat transfer. As the reviewer correctly pointed out, practical implementations will inevitably deviate from this limit due to realistic constraints such as directional feature mismatch, parasitic losses, and fabrication imperfections. Below we provide an estimate of the attainable efficiency by accounting for these main limiting factors.

Directional feature mismatch

We first adopt the realistic microstructural design from scenario 2 (Fig. R1a) as a representative case to estimate the attainable radiative heat transfer efficiency under the same geometric configuration shown in Fig. 1 of the main text. Due to deviations from the ideal emissivity distribution, a directional feature mismatch arises between the emitter and absorber relative to the ideal design conditions, leading to a reduction in the attainable efficiency (Fig. R1b). Nevertheless, System III still improves efficiency from 29% to 74% at an absorber temperature of 50 °C compared with System I.

Fabrication imperfections

We further estimate the radiative heat transfer efficiency of the fabricated emitter (Fig. R1a) by accounting for fabrication imperfections, which introduce additional deviations from the ideal emissivity distribution and further reduce the attainable efficiency (Fig. R1b). Even when realistic fabrication imperfections are considered, System III still improves the efficiency from 29% to 62% at an absorber temperature of 50 °C compared with System I.

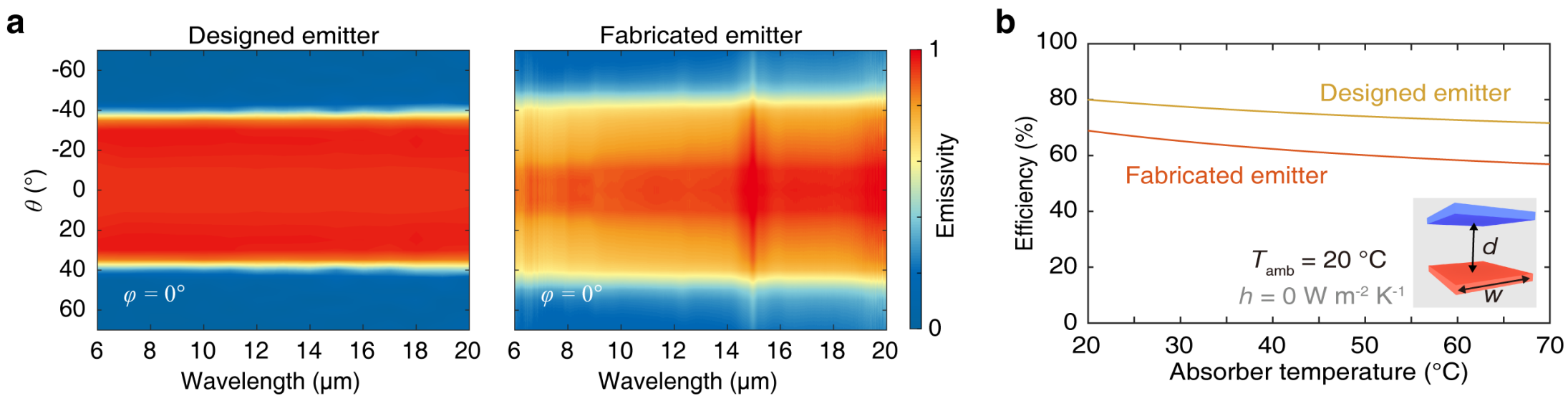

Fig. R1. Radiative heat transfer efficiency under realistic microstructural design and fabrication imperfections. a, Simulated (left) and measured (right) spectral angular emissivity $\varepsilon(\lambda, \theta)$ of the directional emitter. **b,** Radiative heat transfer efficiency as a function of absorber temperature T_{abs} for system III. The efficiency of the designed emitter is reduced due to deviations from the ideal emissivity distribution, while fabrication imperfections further decrease the efficiency of the fabricated emitter. The ambient temperature and parasitic heat transfer coefficient are set to T_{amb} = 20 °C and h = 0 W m^{-2} K^{-1}, respectively. Both the emitter and absorber have a width of w = 100 mm and are placed face-to-face with a separation distance of d = 50 mm.

Parasitic heat transfer losses

To further estimate the influence of parasitic heat transfer losses on radiative heat transfer efficiency, we calculated the efficiency under different parasitic heat transfer coefficients h, using the same geometric configuration as that shown in Fig. 1 of the main text (Fig. R2). As h increase to 3.5 and 7 W m^{-2} K^{-1}, the efficiency of System I decrease to 18.1% and 12.3%, respectively. Although the efficiency limit of System III is reduced in the presence of parasitic heat transfer losses, it still maintains superior performance due to its optimized transfer factors, achieving efficiencies of 45.5% and 24.9%, respectively.

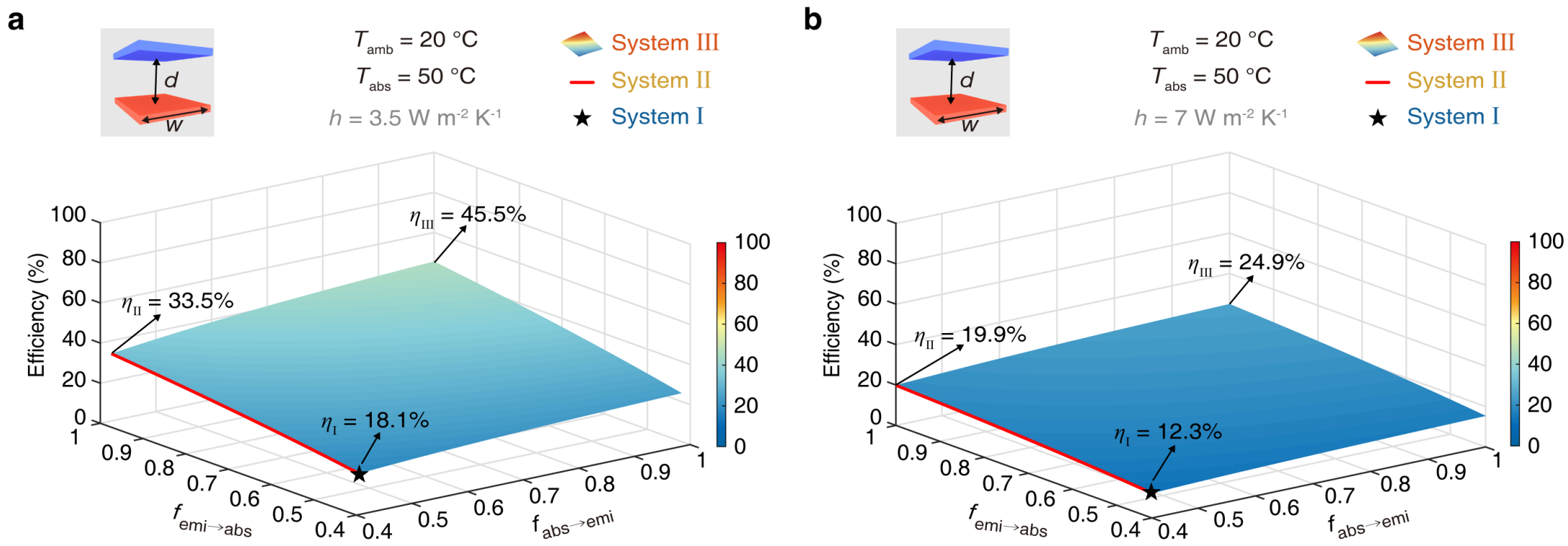

Fig. R2. Radiative heat transfer efficiency with different parasitic heat transfer coefficients. a,b, Radiative heat transfer efficiency η for the three systems as a function of transfer factors $f_{\mathrm{emi}\to\mathrm{abs}}$ and $f_{\mathrm{abs}\to\mathrm{emi}}$ under parasitic heat transfer coefficients h of 3.5 (**a**) and 7 (**b**) W m^{-2} K^{-1}, respectively. Although parasitic heat transfer losses significantly reduce efficiency, System III still achieves the highest efficiency by optimizing the transfer factors. The ambient temperature and absorber temperature are set to T_{amb} = 20 °C and T_{abs} = 50 °C, respectively. Both the emitter and absorber have a width of w = 100 mm and are placed face-to-face with a separation distance of d = 50 mm.

We have included a brief discussion of the practical limitations affecting the attainable efficiency in the revised manuscript.

We have made the below revisions in our revised manuscript.

Revision:

Main text:

Line 161 - 164 on page 5:

Notably, in practical radiative heat transfer scenarios, despite constraints such as parasitic heat transfer losses (Supplementary Fig. 3) and geometric configurations (Supplementary Note 3 and Supplementary Fig. 4), System III consistently achieves the highest efficiency.

Supplementary Information:

Supplementary Fig. 3:

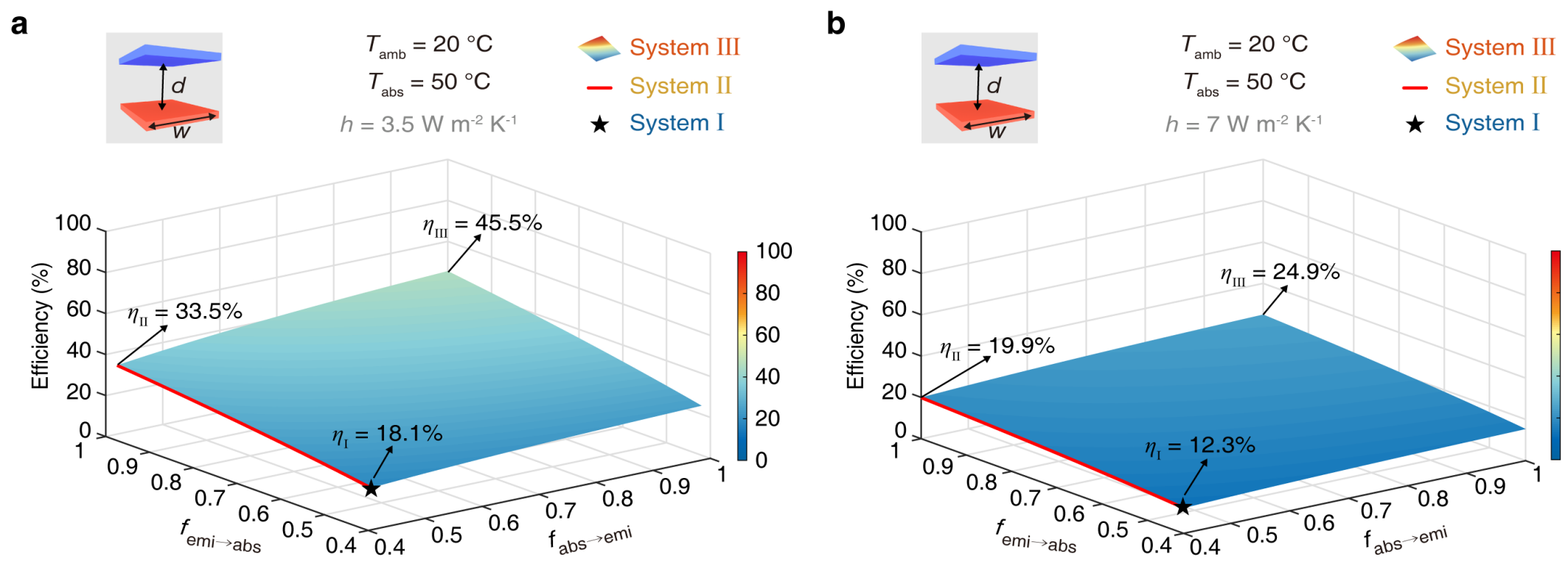

Supplementary Fig. 3. Radiative heat transfer efficiency with different parasitic heat transfer coefficients. a,b, Radiative heat transfer efficiency η for the three systems as a function of transfer factors $f_{emi\to abs}$ and $f_{abs\to emi}$ under parasitic heat transfer coefficients h of 3.5 (**a**) and 7 (**b**) W m^{-2} K^{-1}, respectively. Although parasitic heat transfer losses significantly reduce efficiency, System III still achieves the highest efficiency by optimizing the transfer factors. The ambient temperature and absorber temperature are set to T_{amb} = 20 °C and T_{abs} = 50 °C, respectively. Both the emitter and absorber have a width of w = 100 mm and are placed face-to-face with a separation distance of d = 50 mm.

Comment #2: For scenario 2, the directional emitter exhibits noticeably higher emissivity in the simulations (Fig. S3) than in the experimental measurements (Fig. 2c). Could the authors comment on the possible physical or practical factors that may reduce the emissivity of the fabricated emitter in the desired angular range?

Response: We appreciate the reviewer's comment. The lower measured emissivity compared with simulations is primarily attributed to fabrication-related imperfections, including surface roughness and incomplete Ag film coverage. These factors weaken the collimation effect of the Ag reflector and disrupt the designed optical response, thereby reducing the emissivity in the desired angular range.

To illustrate the actual structural roughness and Ag film coverage, we provide SEM and EDS mapping images of the directional emitter in scenario 2 (Fig. R3). From the SEM images, the structure exhibits pronounced surface undulations and roughness. In addition, the EDS mapping images (insets of Fig. R3) show that the Ag layer progressively decreases in thickness and becomes discontinuous as the position approaches the bottom region of the structure. Such surface roughness and discontinuous Ag coverage enhance diffuse scattering and absorption of the conical surface, reducing the specular reflectivity of the Ag reflector and limiting its ability to direct thermal radiation into the desired angular range. Consequently, a portion of the thermal emission is redistributed into undesired directions, leading to reduced

directional emissivity within the desired angular range.

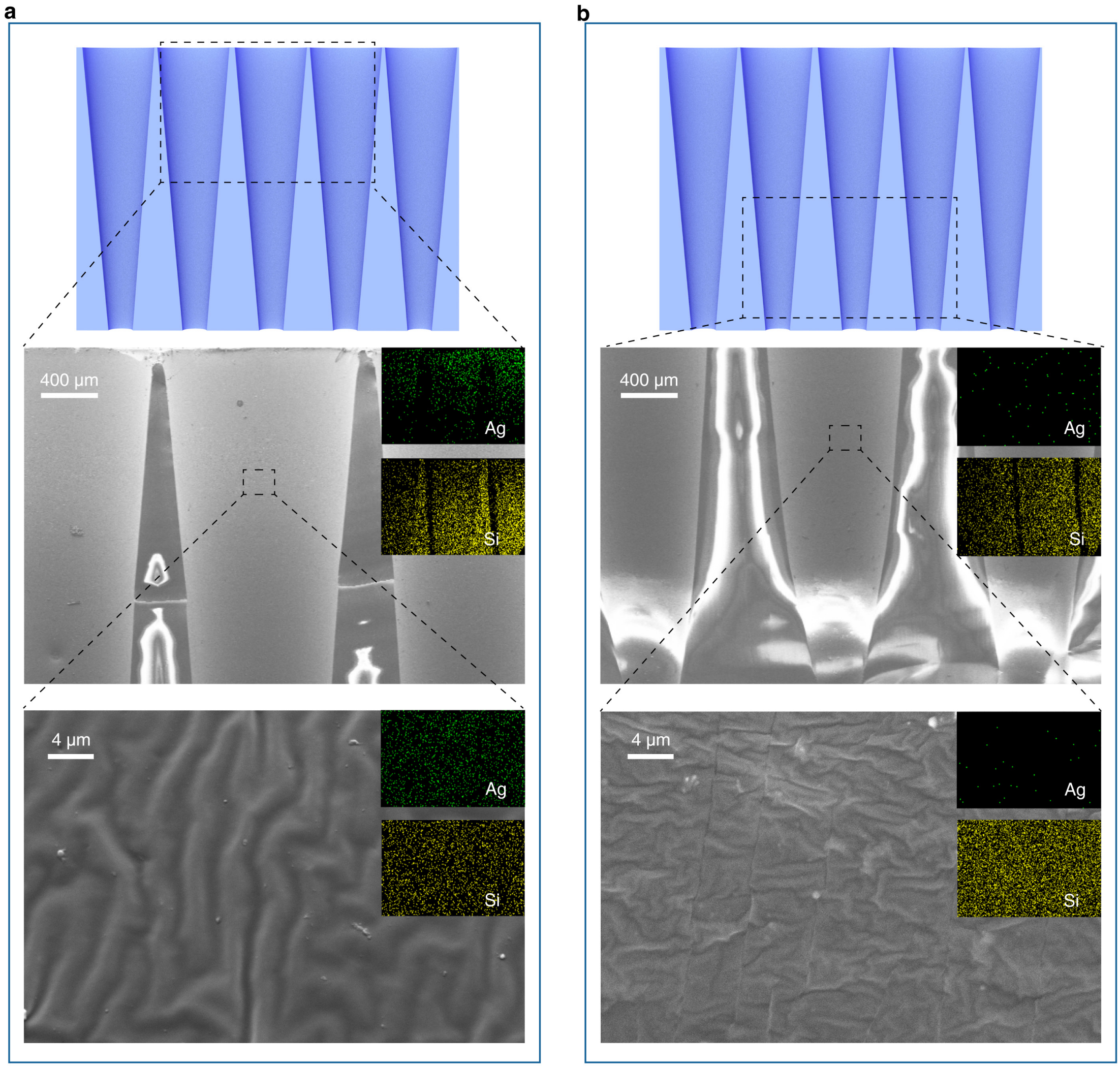

Fig. R3. Cross-sectional SEM images of the directional emitter in scenario 2. a,b, , Cross-sectional SEM images of the upper (a) and lower (b) regions of the directional emitter. The structure exhibits a relatively rough surface with pronounced undulations. EDS mapping images (insets) reveal that, as the position approaches the bottom region, the Ag layer progressively decreases in thickness and becomes discontinuous. The structural roughness and discontinuous Ag coverage reduce the specular reflectivity of the Ag reflector, thereby decreasing the emissivity within the desired angular range.

Comment #3: For Scenario 2, System II requires the highest emitter temperature to heat the absorber to the same target temperature as the other systems, as shown in both Fig. 2g and Fig. 3e. This does not seem to correspond well with the power consumption and efficiency curves in Fig. 2e and Fig. 3c. Could the authors elaborate on the physical mechanism responsible for this behavior? In addition, why does this trend differ from Scenario 1, where System I instead requires the highest emitter temperature, as shown in both Fig. 2f and Fig. 3d?

Response: We appreciate the reviewer's comment. In scenario 2, although the

directional emitter in System II operates at a higher temperature than the blackbody emitter in System I, its directional emission features reduce the total emitted energy, thereby enabling lower power consumption and higher efficiency than System I. By further suppressing radiative losses from the absorber, System III operates at the lowest emitter temperature, resulting in the lowest power consumption and the highest efficiency among the three systems.

In addition, the difference in emitter temperature trends observed between scenario 1 and scenario 2 is primarily attributed to deviations of the experimental fabricated emitters and absorbers from ideal emission features, which arise from practical limitations in microstructural design and fabrication. Below we provide a detailed analysis:

Analysis of emitter temperature and power consumption

In the ideal case, when heating the absorbers to the same target temperature, the blackbody absorbers in Systems I and II would require identical input power, since their total radiative losses are equal at a given absorber temperature (Fig. 1d in the main text). In contrast, the directional absorber in System III suppresses radiative losses, thereby reducing the required input power and resulting in a lower emitter temperature. However, in our experiments, both the directional and blackbody emitters deviate from ideal emission features. In scenario 2, the fabricated directional emitter exhibits lower emissivity than the blackbody emitter within the designed angular range (Fig. R4). This deviation leads to a reduction in the power $P_{\mathrm{emi-abs}}$ received by the absorber from the emitter. Consequently, System II requires a higher emitter temperature than System I to achieve the same absorber temperature. Despite these deviations, System III still achieves the lowest emitter temperature through improved directional matching between the emitter and absorber, further suppressing radiative losses from the absorber.

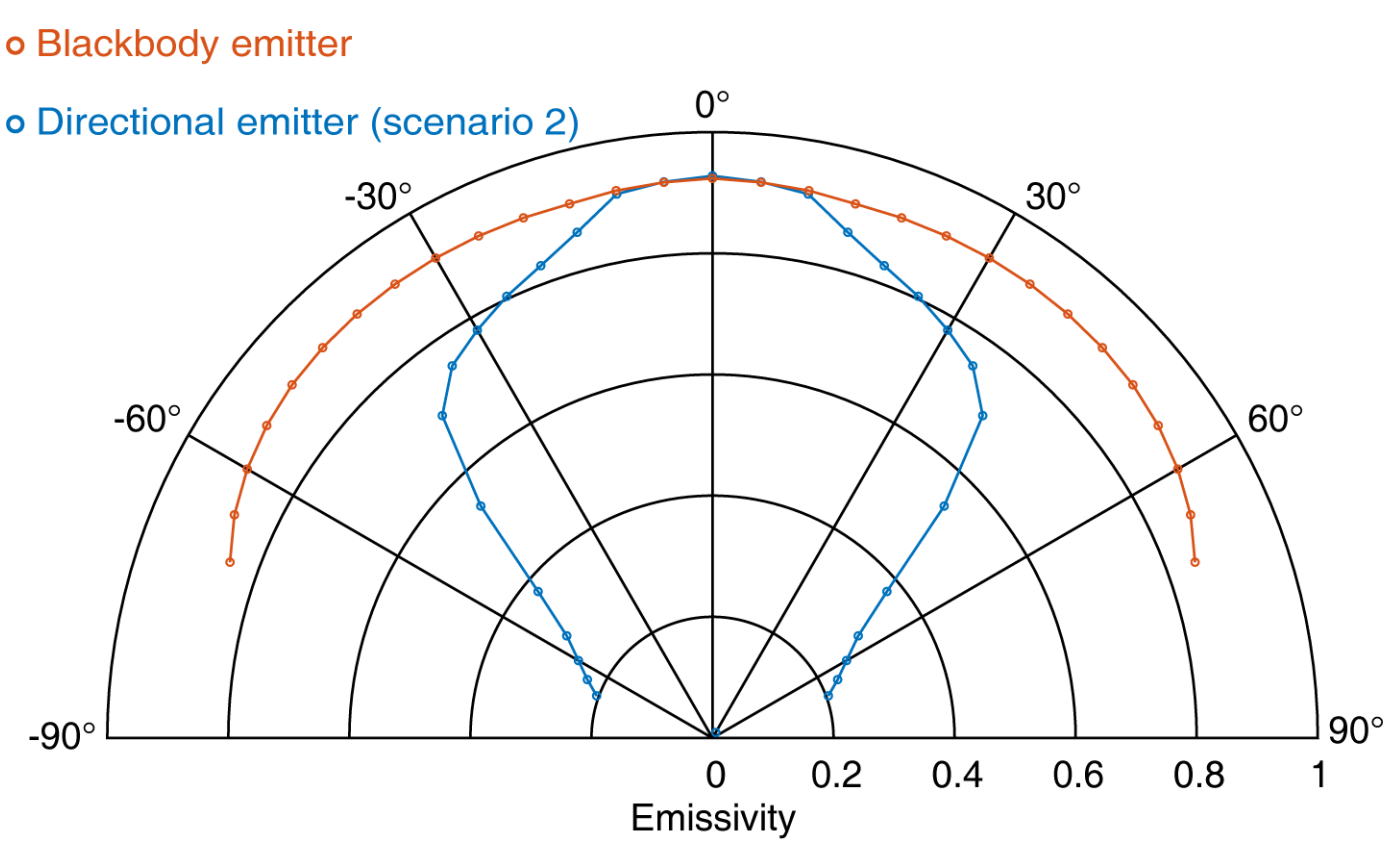

Fig. R4. Measured angular emissivity of emitters in scenario 2. Directional emitter in scenario 2 exhibits lower emissivity than blackbody emitter within the designed angular range.

Although System II requires a higher emitter temperature, its power consumption remains lower than that of System I. To further clarify the power variations among the

three systems, we quantified the relative contributions of each component to the total power consumption, which can be expressed as:

$$P_{\text{consum}} = Q_{\text{emi-bg}} + Q_{\text{emi-abs}} + P_{\text{parasitic1}}$$

where $Q_{\text{emi-bg}}$ is the net heat flux from the emitter to the background, $Q_{\text{emi-abs}}$ is the net heat flux from the emitter to the absorber, and $P_{\text{parasitic1}}$ is the parasitic heat transfer losses from the emitter. Using the experimentally measured emission features, we evaluated these terms at a fixed absorber temperature rise of 4 °C under both vacuum (Fig. R5a-b) and indoor (Fig. R5c-d) conditions. Compared with System I, System II employs a directional emitter that significantly suppresses radiative losses to the background $Q_{\text{emi-bg}}$, thereby reducing the overall power consumption and improving efficiency. System III further minimizes radiative losses by incorporating both a directional emitter and a directional absorber, resulting in the lowest power consumption and highest efficiency. These trends are in good agreement with the experimental results shown in Figs. 2-3 of the main text.

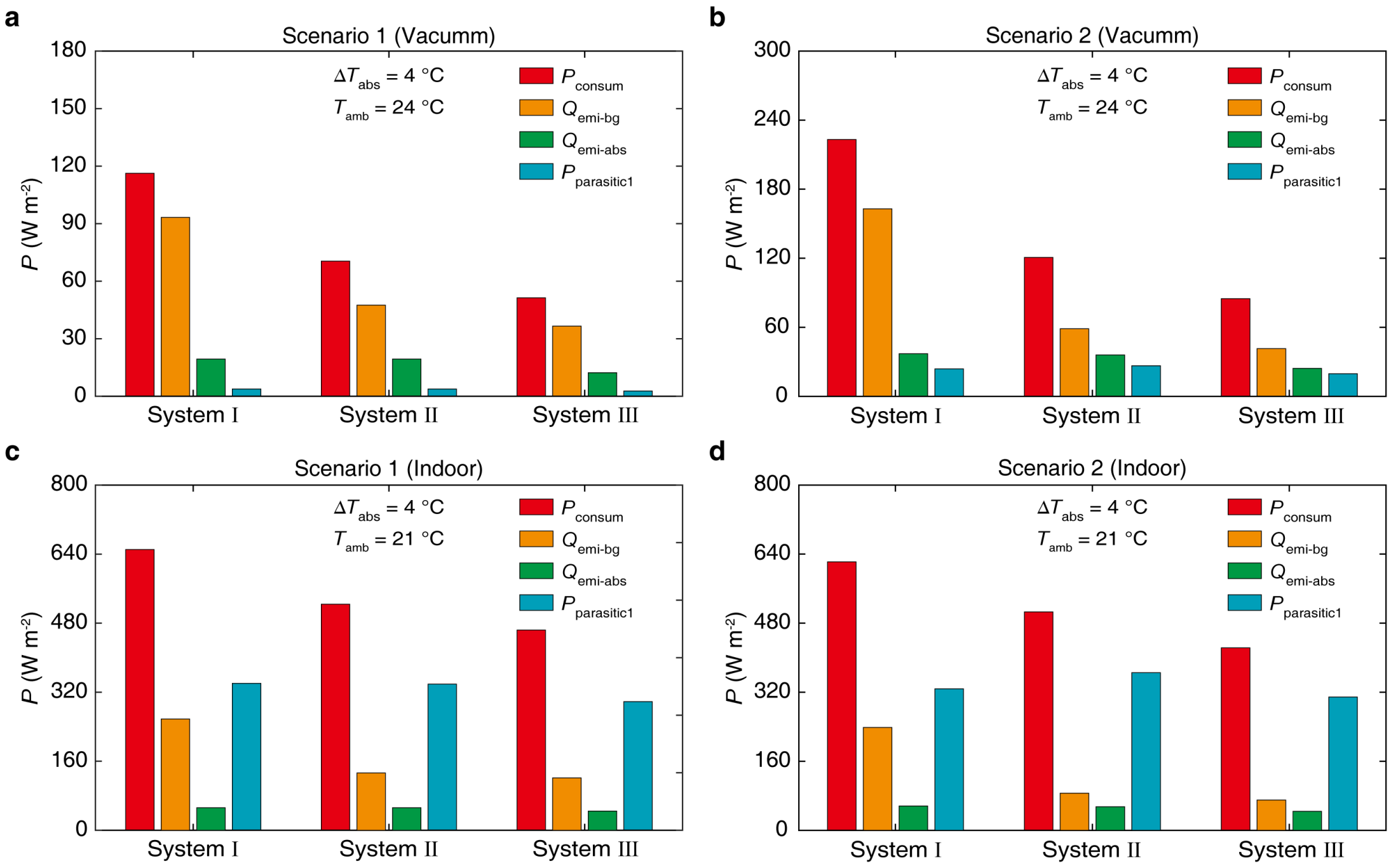

Fig. R5. Calculated relative contributions of each component to power consumption at a fixed absorber temperature rise ΔT_{abs} = 4 ºC. a,b, Power consumption and relative contributions for scenario 1 (**a**) and scenario 2 (**b**) in a vacuum environment, with an ambient temperature T_{amb} = 24 °C. **c,d,** Power consumption and relative contributions for scenario 1 (**c**) and scenario 2 (**d**) in a typical indoor environment, with an ambient temperature T_{amb} = 21 °C. System I consistently exhibits the highest power consumption, while System III shows the lowest, in both vacuum and indoor scenarios.

Different emitter temperature trends

The different emitter temperature trends observed in scenario 1 and scenario 2 are primarily attributed to deviations of the experimentally fabricated emitters from ideal

emission features. In scenario 1, the directional emitter exhibits higher emissivity at large angles than the blackbody emitter used in our experiments (Fig. R6). In contrast, the directional emitter in scenario 2 exhibits lower emissivity than the blackbody emitter (Fig. R4). These differences in emission features give rise to the distinct emitter temperature trends observed in the two scenarios.

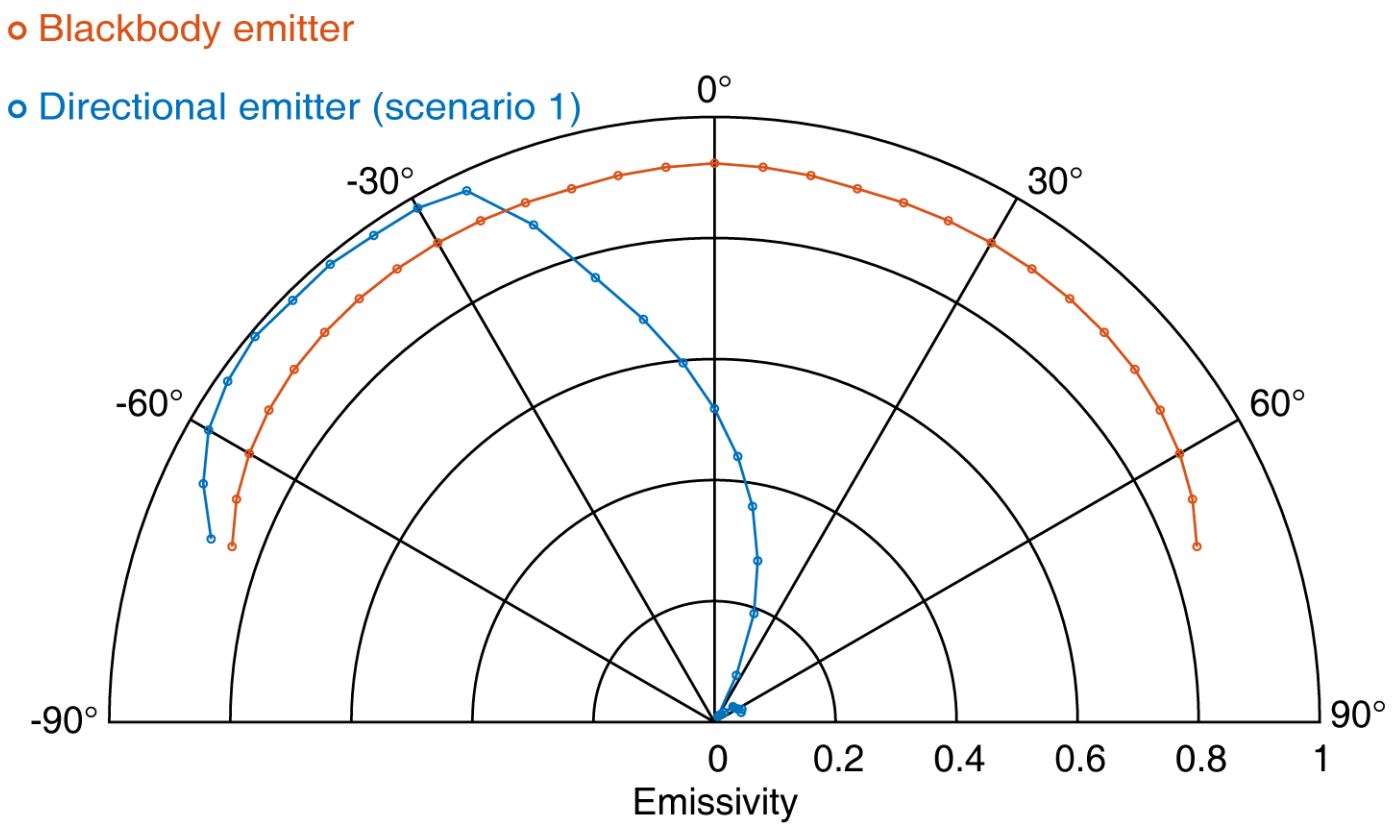

Fig. R6. Measured angular emissivity for emitters in scenario 1. Directional emitter in scenario 1 exhibits higher emissivity than blackbody emitter at large angles.

To further clarify the underlying physical mechanisms, we performed additional calculations for the scenarios in Figs. 2-3 of the main text using experimentally measured emission features. Specifically, we calculated the power received by the absorber from the emitter $P_{\mathrm{emi-abs}}$ and the corresponding absorber temperature rise ΔT_{abs} for all three systems (Fig. R7). In scenario 1, the power received by the blackbody absorber from the blackbody emitter in System I is lower than that from the directional emitter in System II (Fig. R7a). As a result, System I requires a higher emitter temperature to achieve the same absorber temperature increase. In contrast, in scenario 2, the power received by the blackbody absorber from the blackbody emitter in System I exceeds that from the directional emitter in System II (Fig. R7b). Therefore, System II must operate at a higher emitter temperature to reach the same absorber temperature. A similar trend is observed in a typical indoor environment (Fig. R7c-d). In both vacuum and indoor scenarios, System III consistently requires the lowest emitter temperature due to the combined use of a directional emitter and a directional absorber, which effectively suppress undesired radiative heat losses.

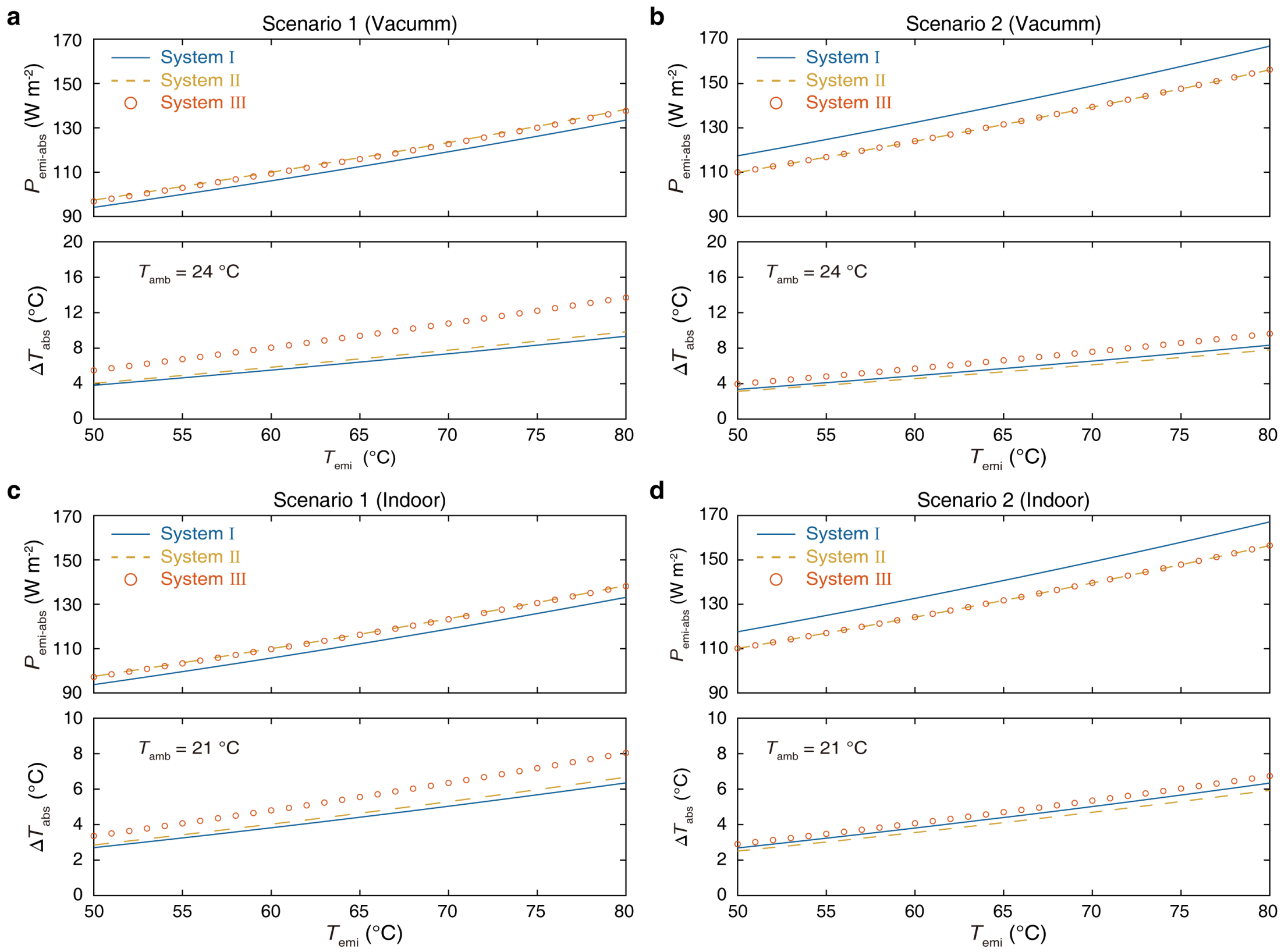

Fig. R7. Impact of emitter temperature T_{emi} on power $P_{emi-abs}$ and absorber temperature rise ΔT_{abs} in vacuum and indoor environments. a,b, $P_{emi-abs}$ and ΔT_{abs} as functions of T_{emi} for scenario 1 (**a**) and scenario 2 (**b**) in a vacuum environment with an ambient temperature T_{amb} = 24 °C. **c,d,** $P_{emi-abs}$ and ΔT_{abs} as functions of T_{emi} for scenario 1 (**c**) and scenario 2 (**d**) in a typical indoor environment with an ambient temperature T_{amb} = 21 °C. All calculations were conducted based on the experimentally measured emission features.

Comment #4: In Fig. 2f, the temperature difference between the emitters in Systems II and III is much larger than that between Systems I and II. However, in the realistic scenario (Fig. 3d), when T_abs=24 C and 27 C, the temperature difference between Systems II and III becomes very small, while the difference between Systems I and II remains noticeable. Could the authors clarify the physical origin of this discrepancy between the idealized and realistic cases?

Response: We appreciate the reviewer's comment. The observed discrepancy between the vacuum and indoor cases originates primarily from slight variations in ambient temperature. These temperature variations further introduce convective and conductive heat losses under indoor conditions. To minimize this influence, we re-conducted the experiments, with all three systems tested simultaneously to ensure identical ambient temperature T_{amb} and convection coefficient. Below we provide a detailed analysis:

Influence of ambient temperature variations

To illustrate the influence of ambient temperature variations, we presented the measured

ambient temperature T_{amb} and the corresponding calculated emitter temperature T_{emi} as functions of T_{amb} in Figs. R8-9. Due to enhanced convective losses under non-vacuum conditions, even slight ambient temperature variations can lead to noticeable differences in emitter temperature T_{emi}. In scenario 1, although the ambient temperature T_{amb} of System II is only approximately 0.5 ºC higher than that of Systems I and III, a nearly 5 °C decrease in emitter temperature is observed for System II (Fig. R8). This results in a significant reduction in the emitter temperature difference between Systems II and III, leading to a trend that differs from that observed under vacuum conditions. A similar behavior is observed in scenario 2 (Fig. R9).

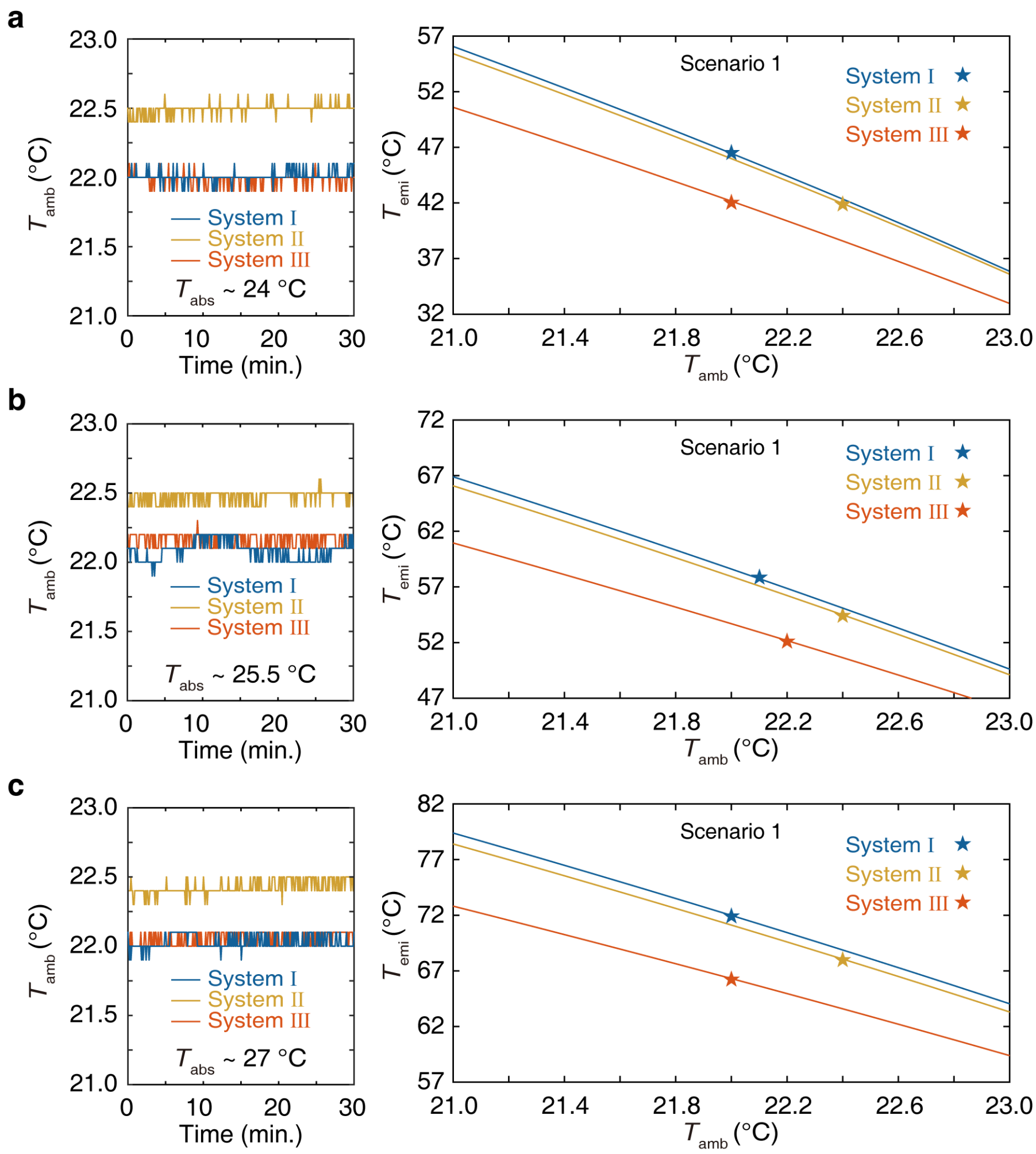

Fig. R8. Measured ambient temperature T_{amb} and calculated emitter temperature T_{emi} as a function of T_{amb} in scenario 1, with absorber temperatures T_{abs} of approximately 24 ºC (a), 25.5 ºC (b), and 27 ºC (c), respectively. Variations in indoor ambient temperature lead to emitter temperature differences that differs from those observed under vacuum conditions.

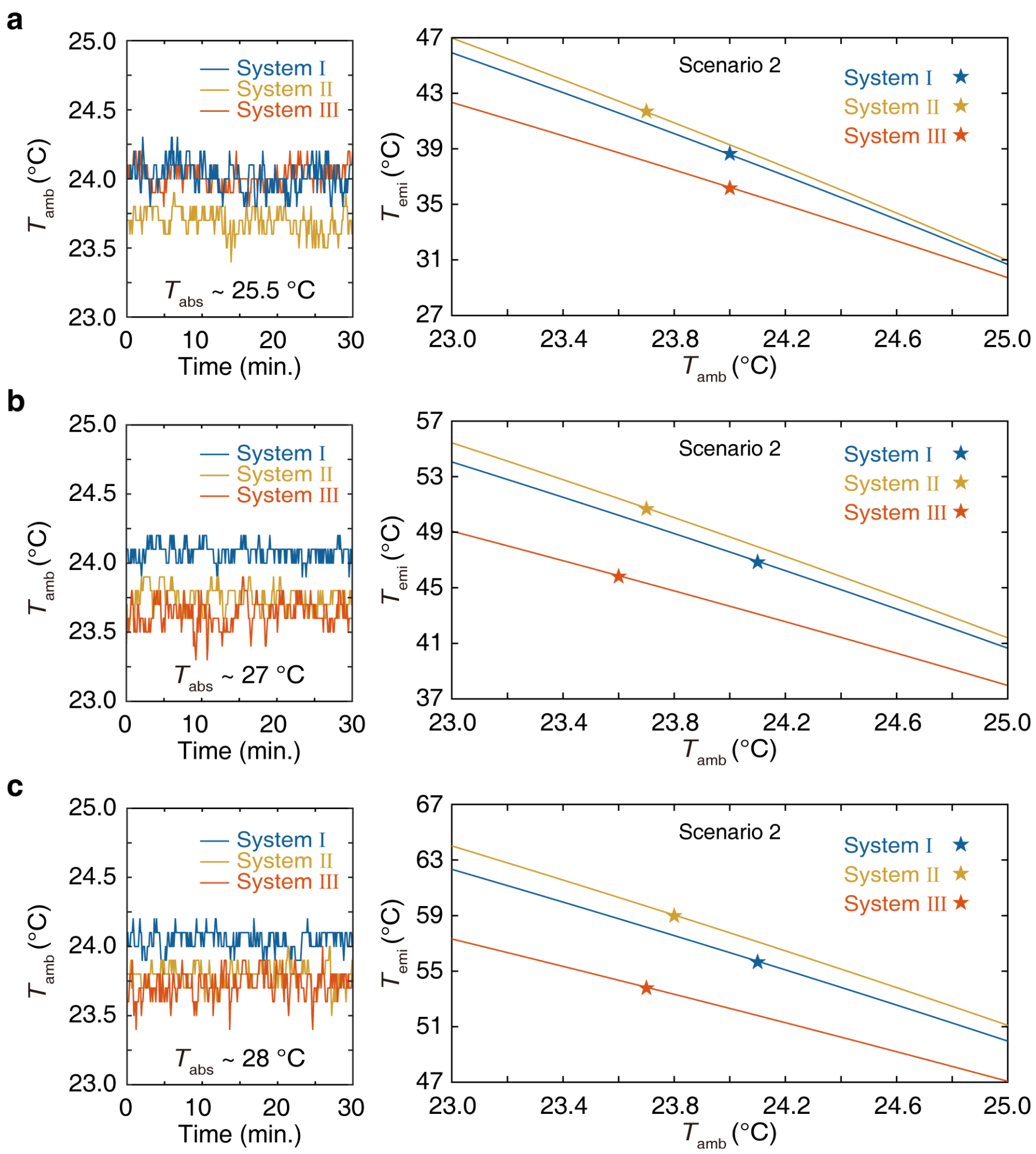

Fig. R9. Measured ambient temperature T_{amb} and simulated emitter temperature T_{emi} as a function of T_{amb} in scenario 2, with absorber temperatures T_{abs} of approximately 25.5 ºC (a), 27 ºC (b), and 28 ºC (c), respectively. Variations in indoor ambient temperature lead to emitter temperature differences that differs from those observed under vacuum conditions.

Indoor experiments with identical ambient temperature

To minimize the influence of ambient temperature variations, we re-conducted the experiments by simultaneously measuring all three systems to ensure identical ambient temperature T_{amb} and convection coefficient. Under this condition, the emitter temperatures measured in both scenario 1 (Fig. R10) and scenario 2 (Fig. R11) show good agreement with the theoretical predictions and follow the trends observed in the vacuum case.

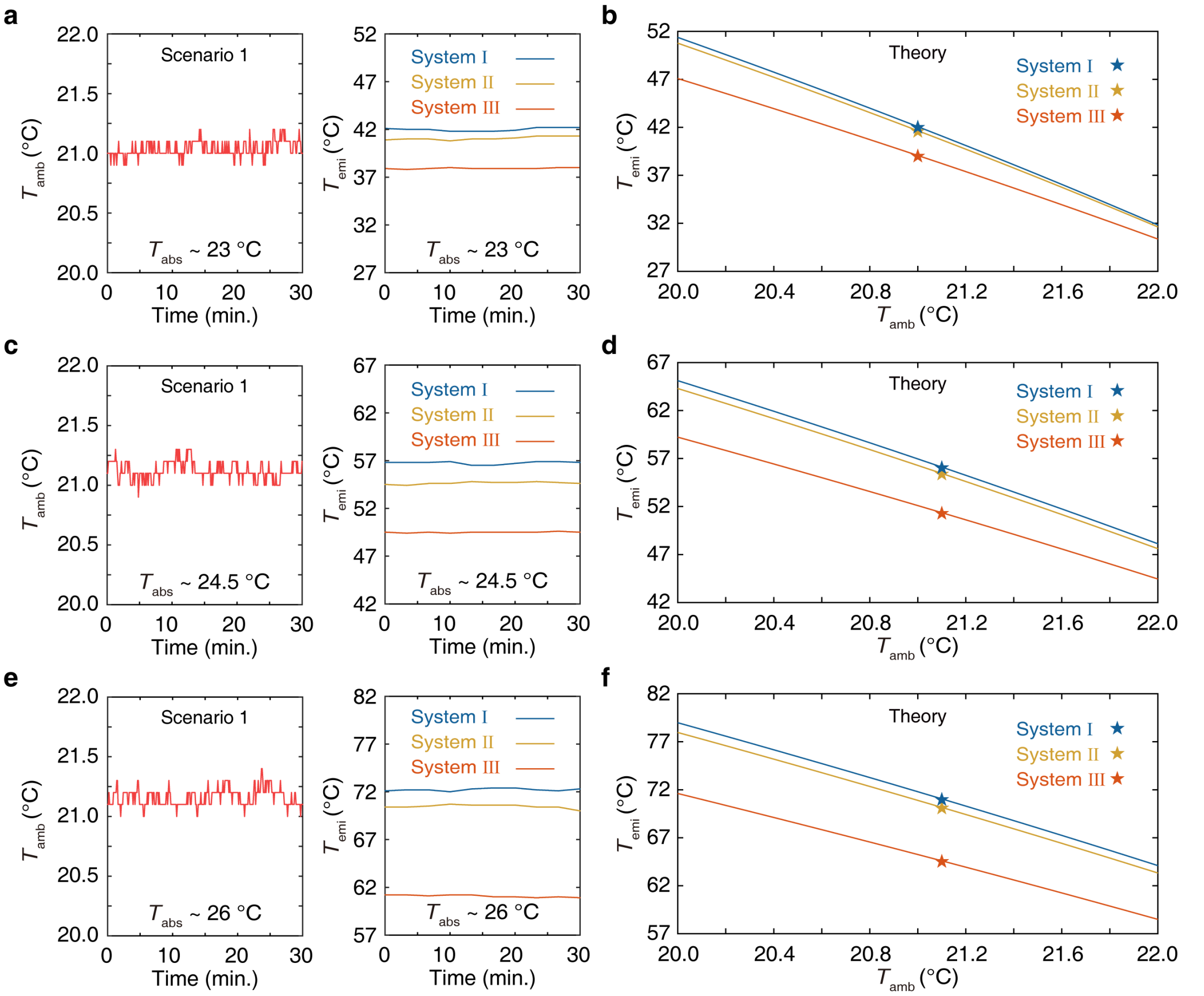

Fig. R10. Experimental results and theoretical predictions of emitter temperature T_{emi} in scenario 1, with absorber temperatures T_{abs} of approximately 23 °C (a,b), 24.5 °C (c,d), and 26 °C (e,f), respectively. The experimentally measured emitter temperatures at the same ambient temperature (**a,c,e**) show good agreement with the corresponding theoretical predictions (**b,d,f**).

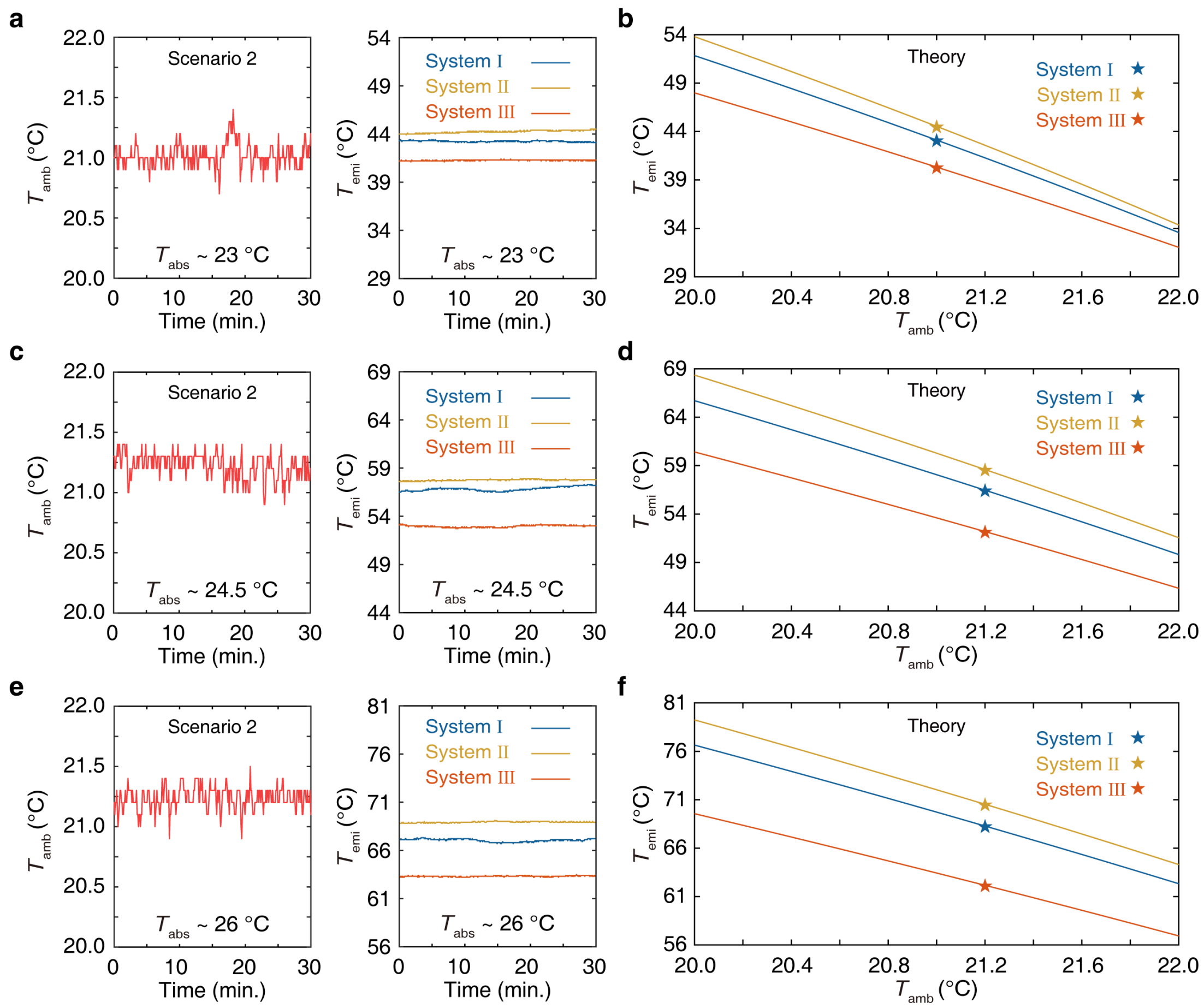

Fig. R11. Experimental results and theoretical predictions of emitter temperature T_{emi} in scenario 2, with absorber temperatures T_{abs} of approximately 23 ºC (a,b), 24.5 ºC (c,d), and 26 ºC (e,f), respectively. The experimentally measured emitter temperatures at the same ambient temperature (**a,c,e**) show good agreement with the corresponding theoretical predictions (**b,d,f**).

As shown in Fig. R12a, in scenario 1, when the absorbers are heated to approximately 26 ºC at an ambient temperature of 21 °C, the power consumption of Systems I, II, and III are 786 W m^{-2}, 668 W m^{-2}, and 565 W m^{-2}, respectively, corresponding to efficiencies of 6.7%, 9.6%, and 11.2%. System III exhibits the lowest emitter temperature, followed by System II and then System I (Fig. R12c). Notably, the temperature difference between the emitters in Systems II and III remains substantially larger than that between Systems I and II, consistent with the trend observed in the vacuum environment (Fig. 2f in the main text). In scenario 2, when the absorbers are heated to approximately 26 ºC, the power consumption of Systems I, II, and III are 752 W m^{-2}, 625 W m^{-2} and 536 W m^{-2}, respectively, corresponding to efficiencies of 8.7%, 10.8%, and 12.6% (Fig. R12b). System III exhibits the lowest emitter temperature, followed by System I and then System II, consistent with the trend observed in Fig. 2g of the main text (Fig. R12d).

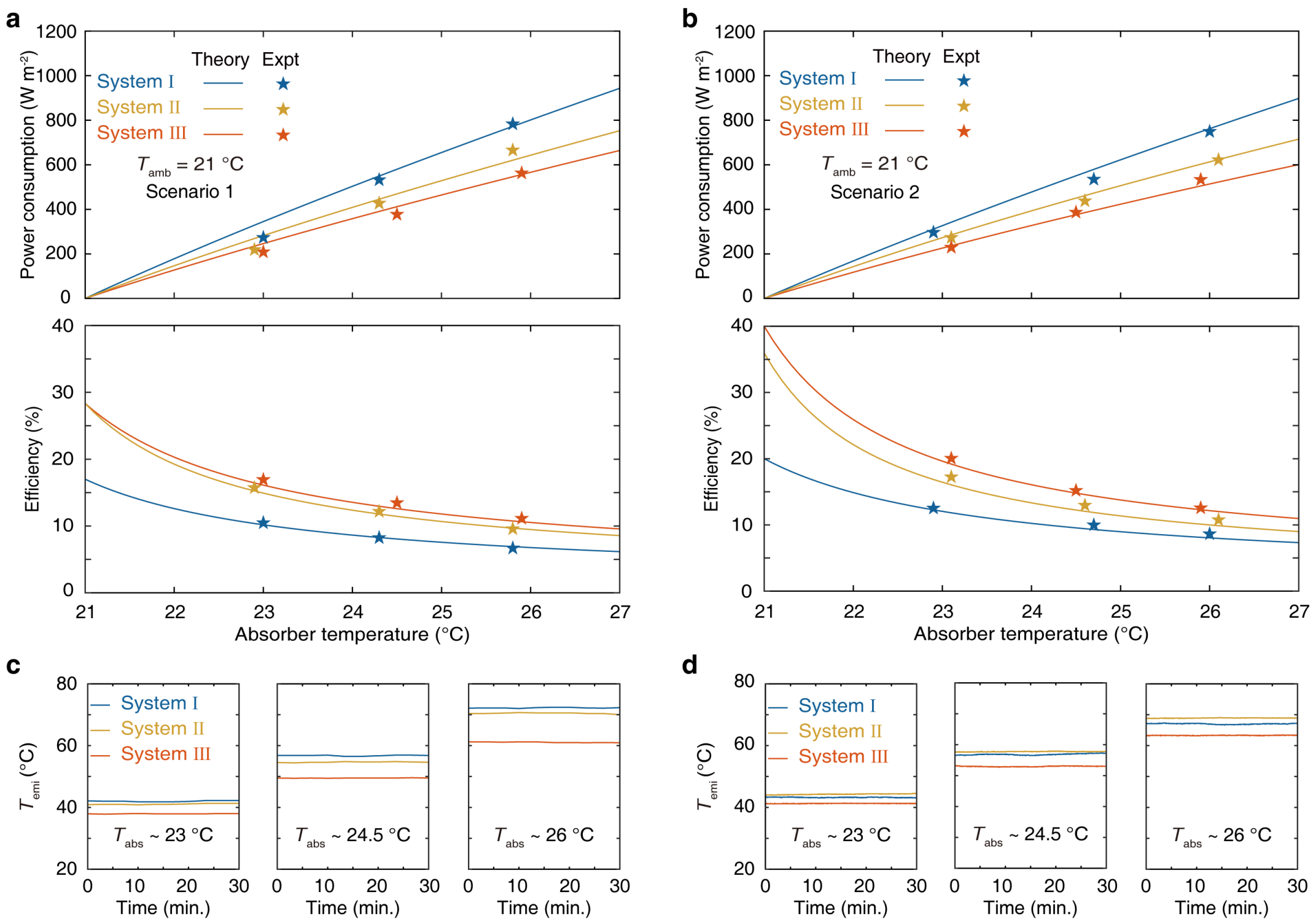

Fig. R12. Demonstration of efficient radiative heat transfer in a typical indoor environment. a,b, Theoretical (lines) and experimental (star-shaped markers) results of power consumption P_{consum} and efficiency η for three systems at different absorber temperatures in scenario 1 (**a**) and scenario 2 (**b**). Although the typical indoor environment introduces additional convection and conduction heat losses, System III (red line) still achieves the highest efficiency and the lowest power consumption compared with System II (yellow line) and System I (blue line) at an ambient temperature of 21 °C. **c,d,** Measured steady-state emitter temperature T_{emi} for three systems in scenario 1 (**c**) and scenario 2 (**d**) with absorber temperatures T_{abs} of approximately 23 ºC, 24.5 ºC, and 26 ºC, respectively.

We have made the below revisions in our revised manuscript.

Revision:

Main text:

Line 240 - 251 on page 10:

The second experiment is conducted in a typical indoor environment to evaluate the practical impact of directional thermal emission (Fig. 3a). All three systems are tested simultaneously to minimize the impact of ambient temperature variations. In the first scenario, when absorbers are heated to approximately 26 ºC (Supplementary Fig. 9), the power consumption of Systems I, II, and III are 786 W m^{-2}, 668 W m^{-2}, and 565 W m^{-2}, respectively, corresponding to efficiencies of 6.7%, 9.6%, and 11.2% (Fig. 3b). System III achieves a 28.1% reduction in power consumption compared with System I. In the second scenario, when absorbers are heated to approximately 26 ºC (Supplementary Fig. 10), the power consumption of Systems I, II, and III are 752 W

m^{-2}, 625 W m^{-2} and 536 W m^{-2}, respectively, corresponding to efficiencies of 8.7%, 10.8%, and 12.6% (Fig. 3c). Similarly, System III achieves a 28.7% reduction in power consumption compared with System I.

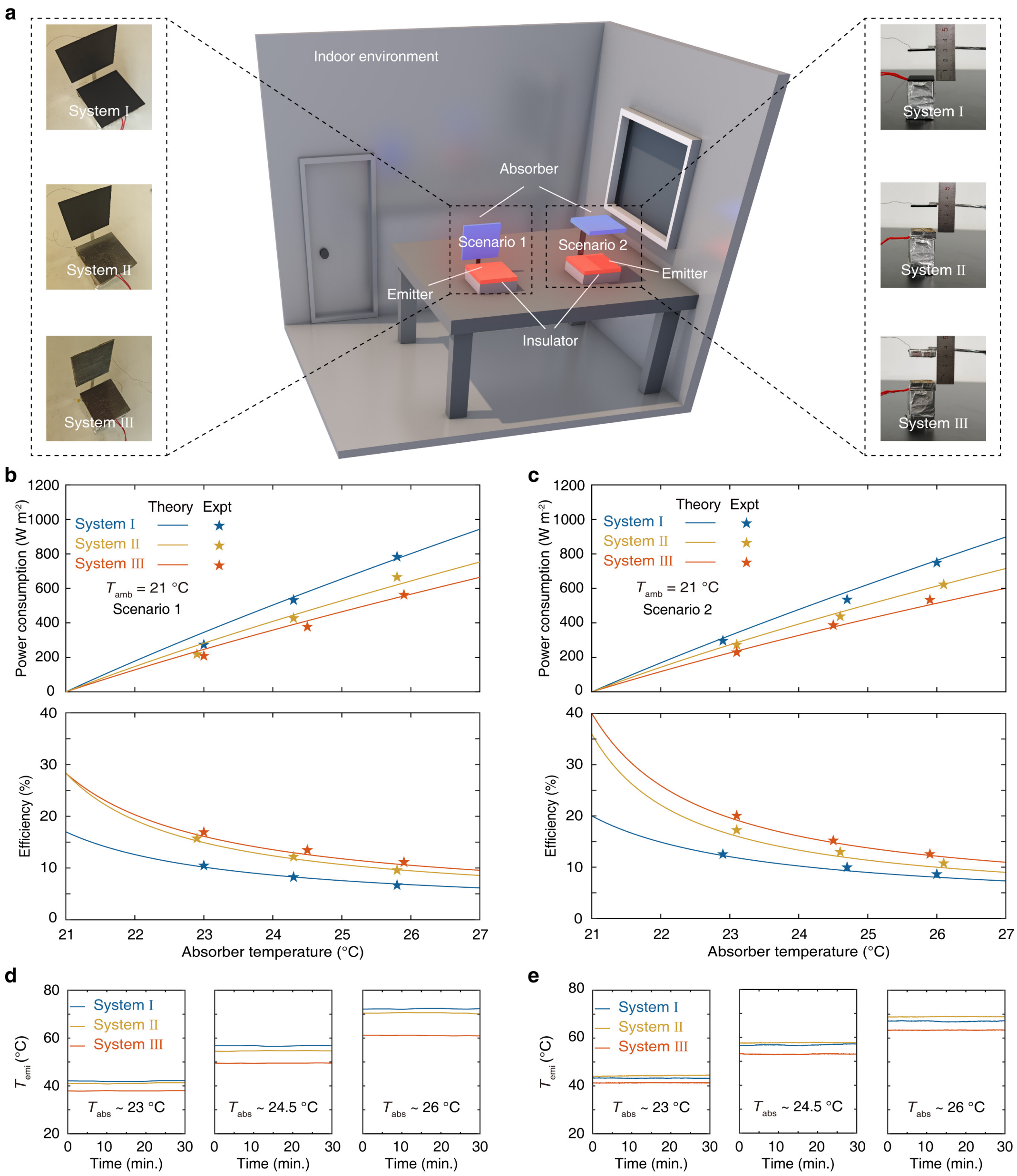

Fig. 3 | Demonstration of efficient radiative heat transfer in a typical indoor environment. a, Schematic of the customized directional radiative heating setup in a typical indoor environment. In scenario 1, an emitter (100 mm by 100 mm) is placed horizontally, while an absorber of the same size is positioned vertically 10 mm from its edge. In scenario 2, an emitter (30 mm by 30 mm) is placed horizontally, with a parallel absorber of the same size positioned 30 mm above it. **b,c,** Theoretical (lines) and experimental (star-shaped markers) results of power consumption P_{consum} and efficiency η for three systems at different absorber temperatures in scenario 1 (**b**) and scenario 2 (**c**). Although the typical indoor environment introduces additional convection and conduction heat losses, System III (red line) still achieves the highest efficiency and the lowest power consumption compared with System II (yellow line) and System I (blue line) at an ambient temperature of 21 °C. **d,e,** Measured steady-state

emitter temperature T_{emi} for three systems in scenario 1 (**d**) and scenario 2 (**e**) with absorber temperatures T_{abs} of approximately 23 ºC, 24.5 ºC, and 26 ºC, respectively.

Supplementary Information:

Supplementary Fig. 9:

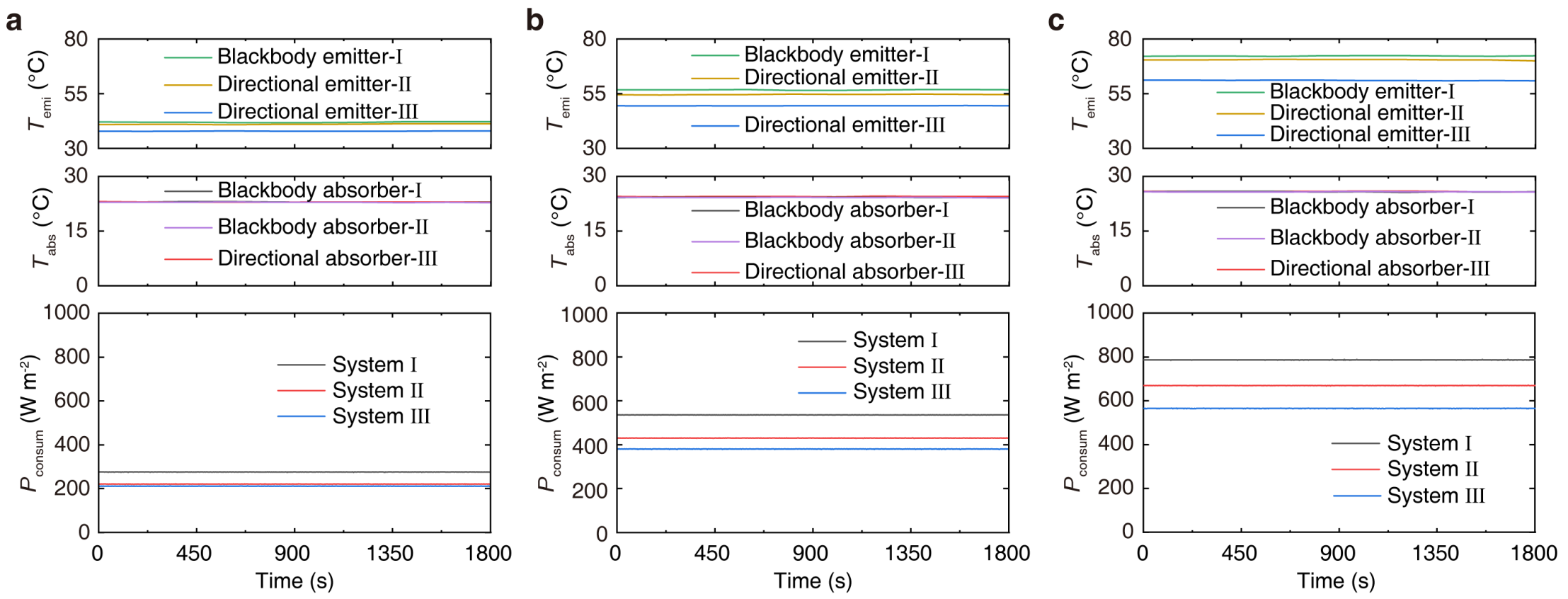

Supplementary Fig. 9. Equilibrium temperature and power consumption of scenario 1 in a typical indoor environment. a-c, The absorbers in the three systems are heated to approximately 23 ºC (**a**), 24.5 ºC (**b**), and 26 ºC (**c**), respectively. When the absorbers are maintained at the same temperature, System III exhibits the lowest emitter temperature T_{emi} and requires the least power consumption P_{consum}.

Supplementary Fig. 10:

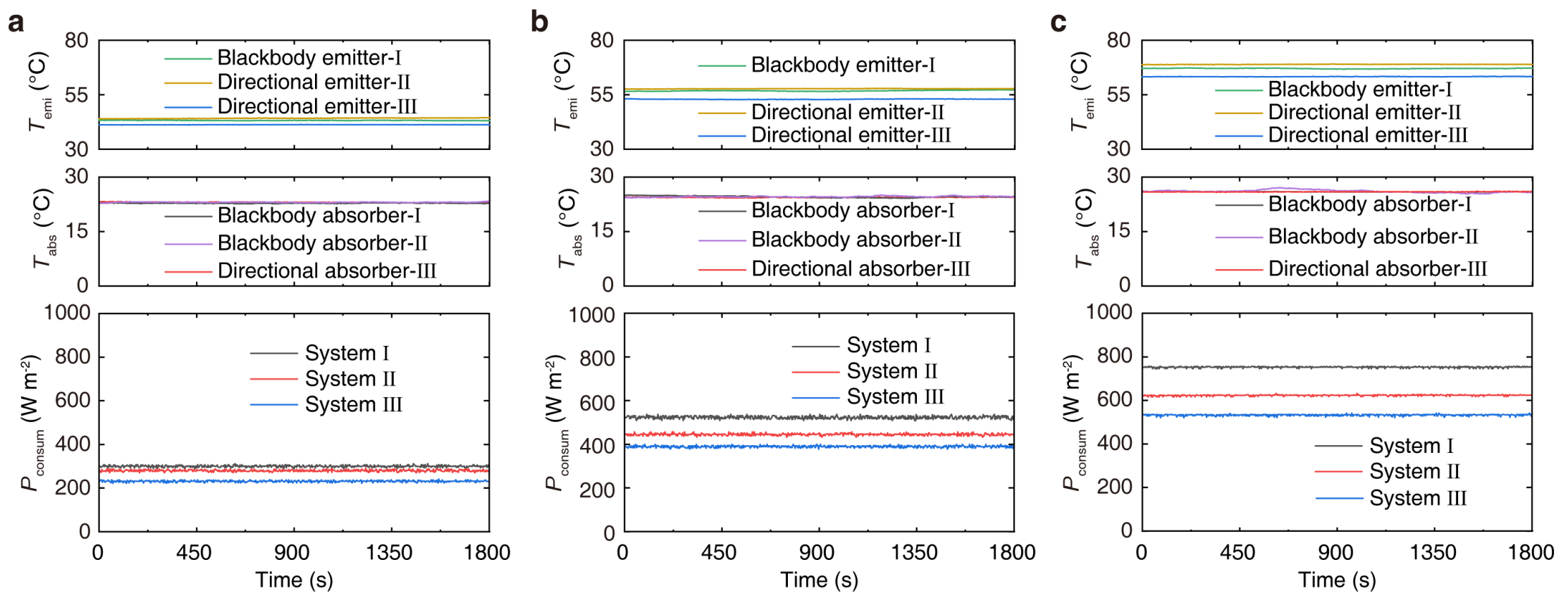

Supplementary Fig. 10. Equilibrium temperature and power consumption of scenario 2 in a typical indoor environment. a-c, The absorbers in the three systems are heated to approximately 23 ºC (**a**), 24.5 ºC (**b**), and 26 ºC (**c**), respectively. When the absorbers are maintained at the same temperature, System III exhibits the lowest emitter temperature T_{emi} and requires the least power consumption P_{consum}.

Comment #5: In Fig. 4a, the mannequin and clothing appear positioned relatively low with respect to the emitter. The authors may wish to comment on the effective view factor or angular overlap between the directional emission lobe and the heated target, and clarify whether geometric mismatch could affect the measured performance.

Response: We appreciate the reviewer's comment. Effective view factor and geometric alignment between the emitter and the target are important factors that can influence the measured performance. However, the apparent mismatch in Fig. 4a of the main text is due to the specific viewing angle of the photograph rather than the actual experimental configuration. To avoid any potential misunderstanding, we have replaced the photograph with one taken from a different viewing angle to more clearly illustrate the relative positions of the emitter and the mannequin (Fig. R13). In the experiments, both the blackbody and directional emitters were carefully aligned with the mannequin to ensure proper angular overlap and to minimize geometric mismatch. The heat flux is primarily concentrated on the front chest region of the mannequin, as shown in Fig. 4e of the main text.

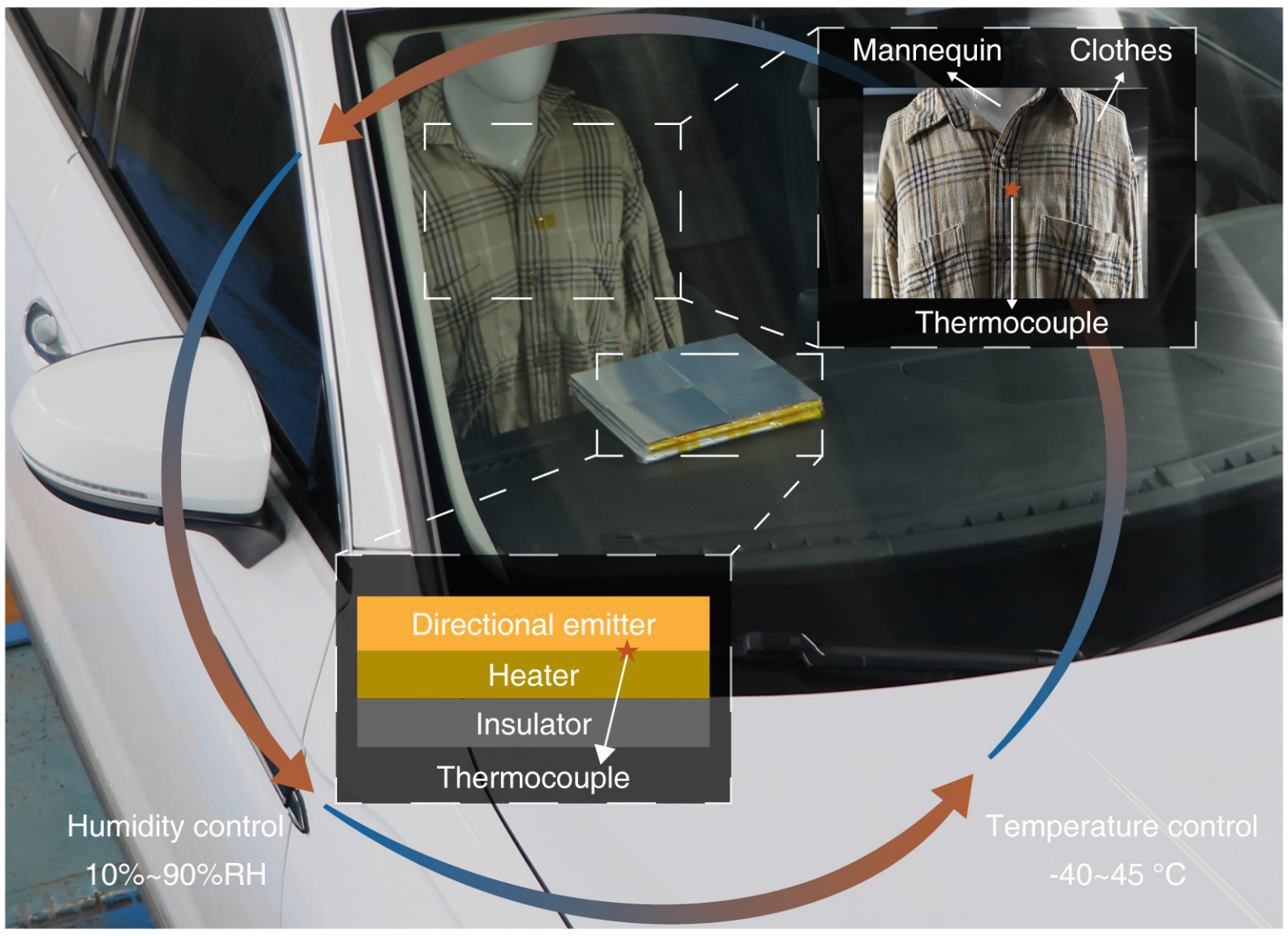

Fig. R13. Photograph of the directional radiative heating setup in an environmental simulation chamber (ESC). The ESC provides control of ambient temperature (-40 °C to 45 °C) and relative humidity (10% to 90%) outside the vehicle.

We have made the below revisions in our revised manuscript.

Revision:

Main text:

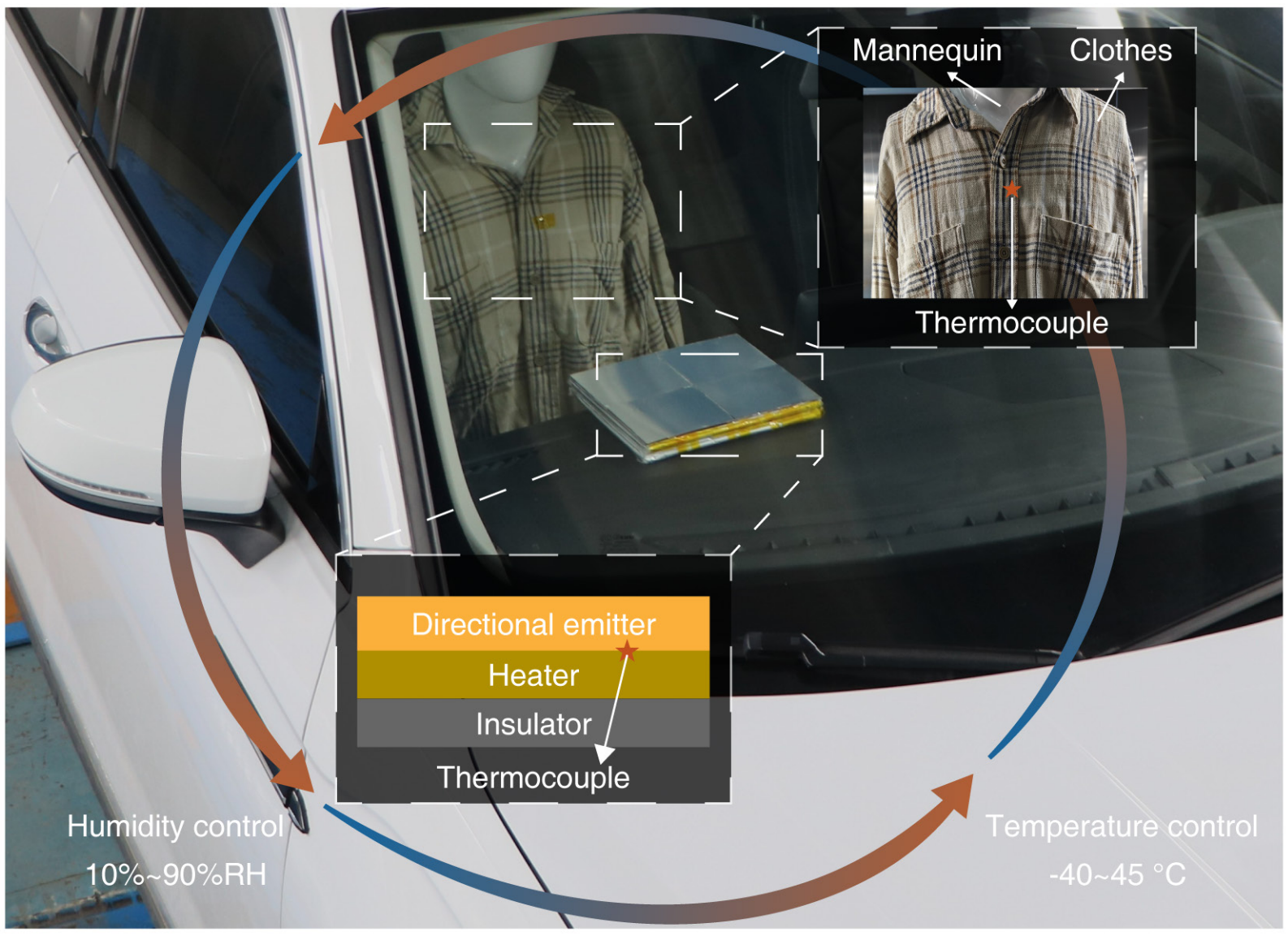

Fig. 4 | Directional radiative heating performance in a vehicle. Photograph of the directional radiative heating setup in an environmental simulation chamber (ESC). The ESC provides control of ambient temperature (-40 °C to 45 °C) and relative humidity (10% to 90%) outside the vehicle.

Comment #6: In Fig. 3a, the caption states a separation of 30 mm between the parallel plates. From the photograph, the spacing appears visually larger. The authors may wish to confirm the actual separation distance and clarify the measurement definition.

Response: We appreciate the reviewer's comment. The observed discrepancy arises from variations in the viewing angle of the photographs. To avoid any potential misunderstanding, we have taken new photographs of the three systems from the same viewing angle and included a ruler to explicitly indicate the actual separation distance between the emitter and absorber (Fig. R14).

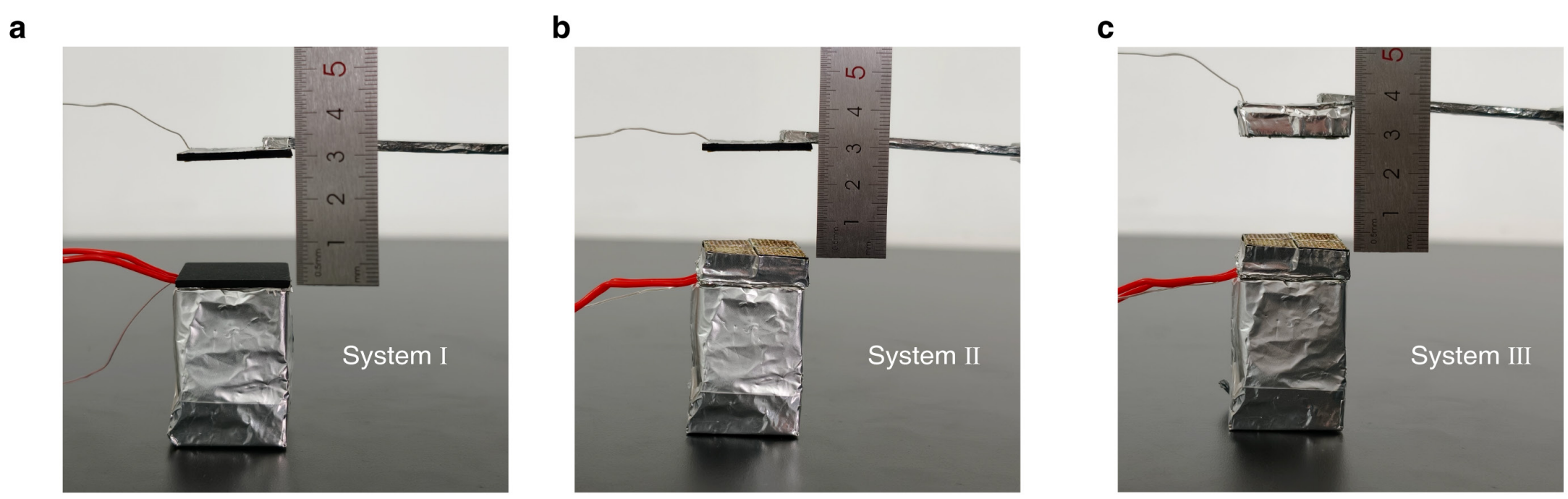

Fig. R14. Photographs of the experimental setup for scenario 2 in Systems I (a), II (b), and III (c). In each system, an emitter (30 mm by 30 mm) is placed horizontally, with a parallel absorber of the same size positioned 30 mm above it. A ruler is included

to indicate the actual separation distance.

We have made the below revisions in our revised manuscript.

Revision:

Main text:

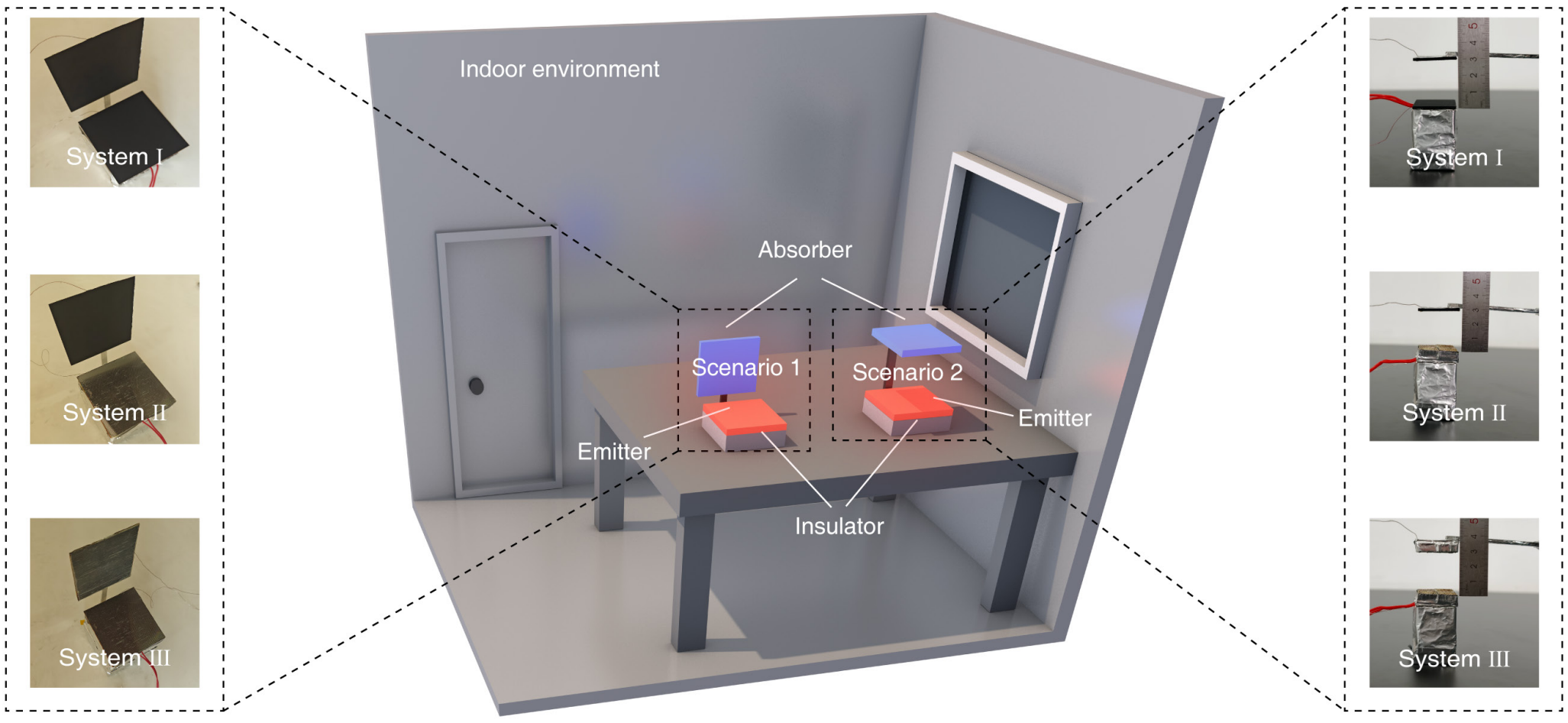

Fig. 3 | Demonstration of efficient radiative heat transfer in a typical indoor environment. Schematic of the customized directional radiative heating setup in a typical indoor environment. In scenario 1, an emitter (100 mm by 100 mm) is placed horizontally, while an absorber of the same size is positioned vertically 10 mm from its edge. In scenario 2, an emitter (30 mm by 30 mm) is placed horizontally, with a parallel absorber of the same size positioned 30 mm above it.

Comment #7: The authors may wish to clarify that the analysis is dominated by mid-infrared thermal radiation and comment briefly on whether shorter-wavelength absorption plays any measurable role in their experiments.

Response: We appreciate the reviewer's comment. In both our theoretical analysis and experiments, the shorter-wavelength thermal radiation (or absorption) can be neglected. Radiative heat transfer is dominated by mid-infrared thermal radiation due to broadband thermal emission of emitters and the operating temperature range of 20-100 °C.

According to Planck's radiation law, the spectral distribution of thermal radiation is determined by surface emissivity and temperature. Based on the emissivity of our designed emitter, we calculated the emissive power spectra of the directional emitters in scenario 1 and scenario 2. As shown in Fig. R15, at temperatures of 20 °C and 100 °C, the emissive power is predominantly concentrated in the mid-infrared region. The contribution from shorter wavelengths (1-3 μm) accounts for only approximately 0.005-0.08% of the total emissive power within the 1-20 μm range. Therefore, shorter-wavelength thermal radiation (or absorption) does not play a measurable role in the experiments considered in this work.

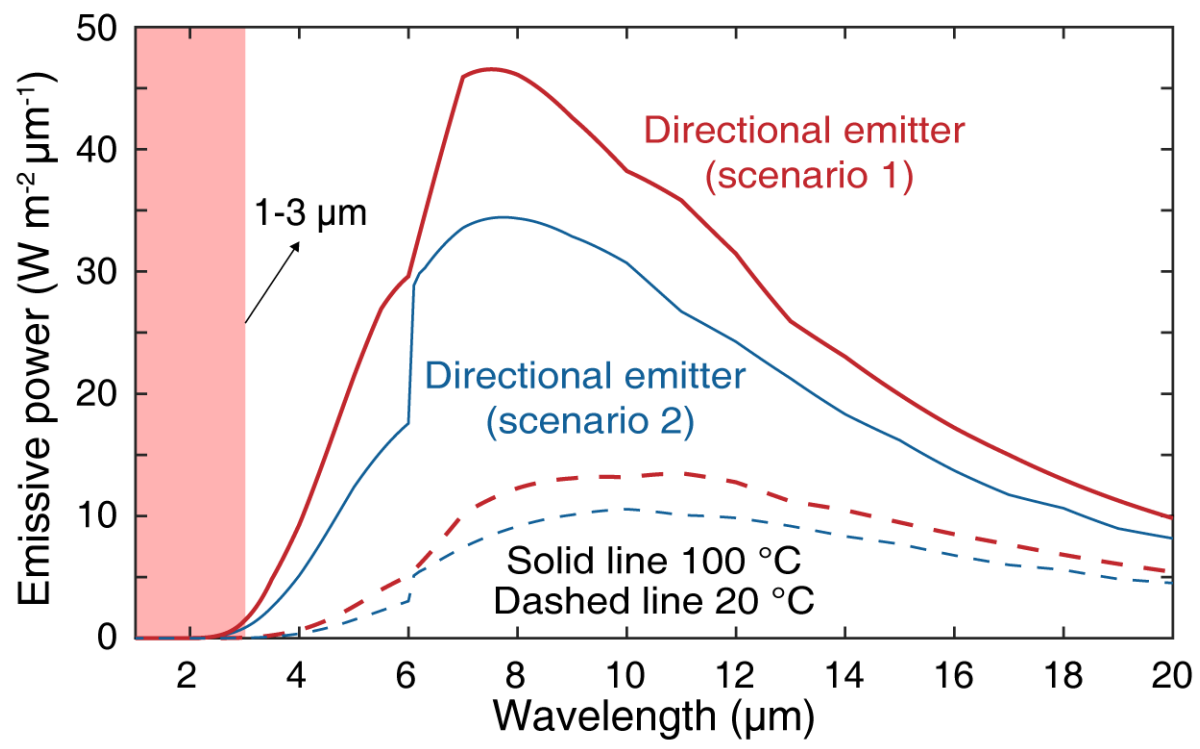

Fig. R15. Emissive power spectra of the directional emitters over the wavelength range of 1-20 μm. The emissive power for the directional emitters in scenario 1 (red) and scenario 2 (blue) is predominantly concentrated in the mid-infrared region at operating temperatures of 20 °C (dashed line) and 100 °C (solid line).

Comment #8: In Supplementary Note 3, the thermal conductivity values appear to be given in units of $W \cdot m^{-2} \cdot K^{-1}$, whereas thermal conductivity should have units of $W \cdot m^{-1} \cdot K^{-1}$.

Response: We appreciate the reviewer's comment. The units in Supplementary Note 4 have been corrected to $W\ m^{-1}\ K^{-1}$ in the revised manuscript.

We have made the below revisions in our revised manuscript.

***Revision*:**

Supplementary Information:

Supplementary Note 4:

Line 188 - 189 on page 7:

For the emitters in scenario 1, with r= 0.79% and k_{peg}= 0.5 $W\ m^{-1}\ K^{-1}$ conductivity

Line 201 - 202 on page 7:

3 mm thick acrylic, with thermal conductivity $k_{acrylic}$= 0.19 $W\ m^{-1}\ K^{-1}$

We extend our deep gratitude once again to the reviewer for recognizing the potential impact and remarkable significance of our work, as well as for providing constructive suggestions. All comments have been carefully addressed, with point-by-point revisions and responses provided as above. We hope the reviewer will find our responses satisfactory.

Response to Reviewer #2:

Reviewer #2: Thermal radiation is a fundamental heat transfer mechanism in many energy-related systems, including building heating, industrial processes, transportation, and human thermal comfort. Despite its importance, radiative heat transfer in practical systems is commonly treated as omnidirectional, which inevitably leads to energy dissipation toward non-target regions and constrains overall system efficiency. While recent advances in photonic and thermal design have enabled increasing control over the directionality of thermal emission, the system-level implications of directional radiative heat transfer, particularly in realistic operating environments, remain insufficiently explored. This manuscript investigates directional radiative heat transfer through a combination of a generalized theoretical framework and experimental demonstrations conducted under vacuum, indoor, and application-oriented conditions. By introducing the transfer factor, the work provides a unified description of directional radiative coupling that extends beyond conventional view-factor-based approaches. The accompanying experiments illustrate how directional thermal emission can influence energy consumption in several representative heating scenarios across different environments. Overall, the study addresses a topic of clear relevance to thermal management and energy-related applications and contributes a systematic analysis of directional radiative heat transfer that bridges theory and experiment. Therefore, it is a timely and exciting research that warrant consideration by Nature Comm.

Response: We express our gratitude to the reviewer for the thoughtful summary and for recognizing the timeliness and excitement of our work. We also appreciate the reviewer's insightful comments and suggestions which we address point by point as below.

Comment #1: Major issues:
In the theoretical analysis presented in Figs. 1–3, the manuscript emphasizes the advantages of System III, in which both the emitter and the absorber exhibit directional characteristics and are directionally matched to achieve the highest radiative heat transfer efficiency. However, the application-oriented demonstrations, particularly the vehicle heating experiment in Fig. 4, appear to correspond more closely to the configuration of System II, since the human body and clothing, acting as absorbers in this scenario, generally behave as broadband, nearly blackbody absorbers without directional selectivity. A similar issue may also arise in the practical scenarios illustrated in Fig. 5, where the absorbers do not appear to exhibit directional absorption characteristics. The authors should clarify this apparent disconnection between the theoretical framework and the application demonstrations, and clearly define the scope within which the advantages of System III are expected to be realized.

Response: We appreciate the reviewer's comment. We agree that the theoretical framework presented in Fig. 1 of the main text, together with the experimental

demonstrations in Figs. 2-3 of the main text, clearly highlights the advantages of System III, in which matched directional thermal emission between the emitter and absorber are achieved, thereby enabling the highest radiative heat transfer efficiency. Accordingly, the full advantages of System III are expected in applications where both emitters and absorbers can be engineered with directional features. In contrast, the application-oriented demonstrations in Figs. 4-5 of the main text represent realistic scenarios that more closely correspond to the configuration of System II, where the emitter exhibits directional features while the absorber behaves approximately as a blackbody.

We note that the purpose of the application demonstrations is not to fully reproduce the idealized System III configuration, but rather to evaluate the practical impact of introducing directional emission on system-level performance under realistic absorber conditions. Considering current standards in the automotive and apparel industries, directional thermal radiation control technologies and related devices have not yet been implemented in these fields. Therefore, we analyzed representative practical scenarios to assess the potential energy savings achievable under realistic conditions. With ongoing advances in directional thermal radiation control, we believe that integrating such capabilities into practical applications will further improve efficiency and energy savings, thereby extending the advantages demonstrated for System III toward real-world implementations.

Comment #2: Page 4: In Fig. 1, the theoretical analysis adopts a configuration consisting of two planar surfaces with fixed dimensions and a defined separation distance. While the geometric parameters are specified, the dependence of the transfer factor f on geometric variables such as separation distance, relative orientation, and lateral misalignment is not explicitly discussed. Since geometric coupling fundamentally governs radiative exchange, this dependence should be clarified. A supplementary discussion or parametric analysis showing how variations in distance or relative angle influence f and the efficiency η is necessary. Even a limited example would help readers understand the geometric robustness and applicability of the proposed framework.

Response: We appreciate the reviewer's comment. Geometric coupling plays a significant role in radiative heat transfer, particularly in System I, where the transfer factor decreases with increasing separation distance, relative orientation, and lateral misalignment, resulting in reduced efficiency. In contrast, Systems II and III mitigate this sensitivity by introducing directional thermal emission, which enhances the transfer factor across a broad range of geometric configurations, highlighting the geometric robustness and broad applicability of our framework. Below we provide a detailed analysis:

To clarify the impact of geometric factors on radiative exchange, we have analyzed the

dependence of the transfer factor f and radiative heat transfer efficiency η on several key parameters, including separation distance, relative orientation, and lateral misalignment (Fig. R16).

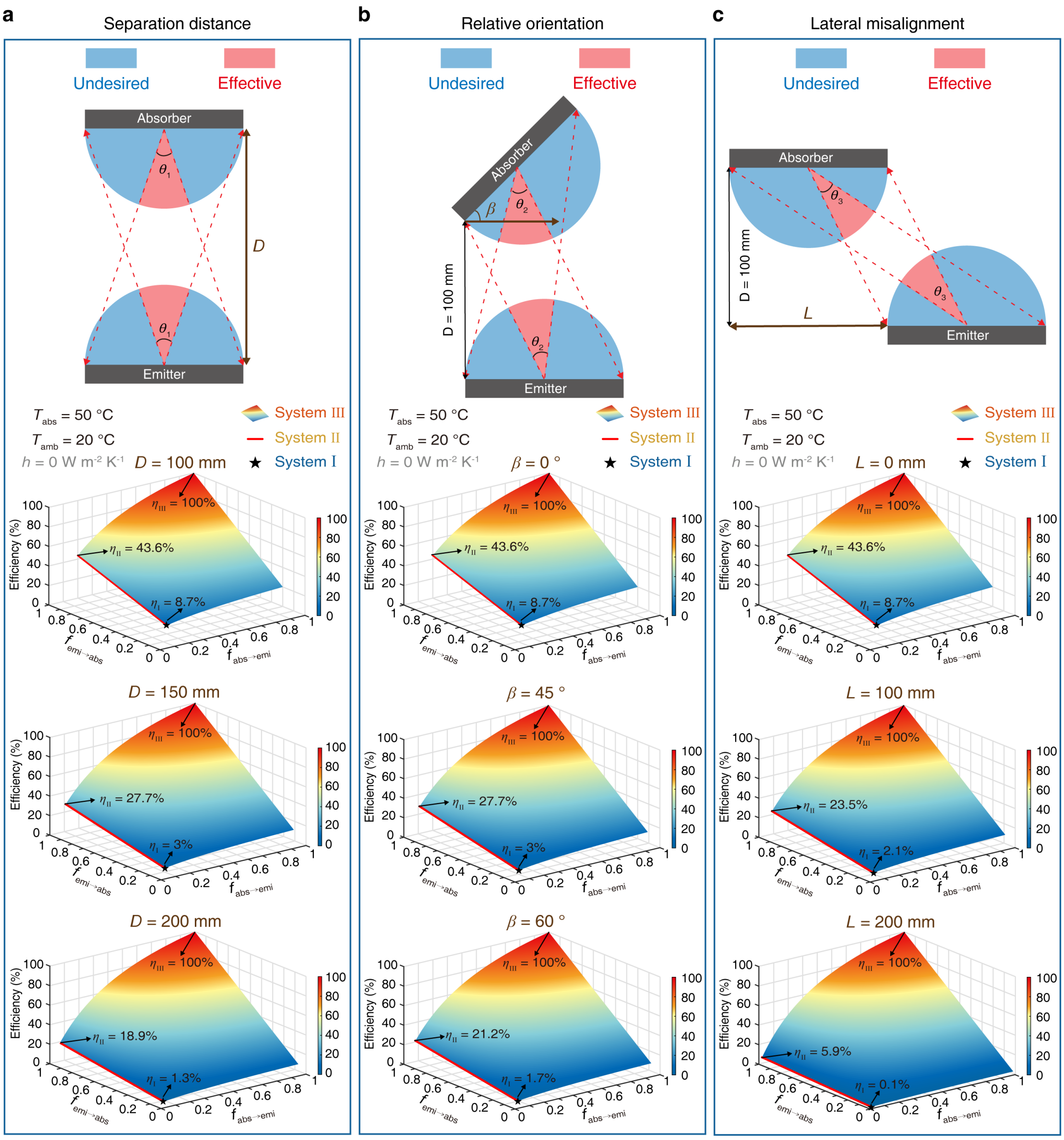

Fig. R16. Theoretical radiative heat transfer efficiency η under different geometric configurations. a, Schematic of two surfaces (100 mm by 100 mm) placed face-to-face with a separation distance of D. The theoretical radiative heat transfer efficiencies of the three systems at different separation distances are shown below. **b,** Schematic of two surfaces (100 mm by 100 mm) oriented with a relative angle β at a fixed separation distance of 100 mm. The theoretical radiative heat transfer efficiencies of the three systems at different relative angles are shown below. **c,** Schematic of two surfaces (100 mm by 100 mm) with lateral misalignment L at a fixed separation distance of 100 mm. The theoretical radiative heat transfer efficiencies of the three systems at different lateral misalignments are shown below.

Separation distance

First, we consider an emitter placed face-to-face with an absorber, each with dimensions of 100 mm by 100 mm. As the separation distance D increases from 100 to 150 and 200 mm, the corresponding transfer factors $f_{\mathrm{emi}\to\mathrm{abs}}$ and $f_{\mathrm{abs}\to\mathrm{emi}}$ of System I decrease from 0.20 to 0.11 and 0.07, respectively, leading to efficiencies of 8.7%, 3.0%, and 1.3% (Fig. R16a). This clearly indicates that, in conventional blackbody systems, efficiency is strongly limited by separation distances and decreases rapidly as the distance increases. In contrast, at a separation distance of 200 mm, System II optimizes the directional features of the emitter according to the geometric configuration, increasing the transfer factor $f_{\mathrm{emi}\to\mathrm{abs}}$ from 0.07 to 1 and thereby improving efficiency from 1.3% to 18.9%. System III further achieves the theoretical upper limit of 100% efficiency by simultaneously optimizing both $f_{\mathrm{emi}\to\mathrm{abs}}$ and $f_{\mathrm{abs}\to\mathrm{emi}}$.

Relative orientation

Second, we consider an emitter with a relative angle β of 0°, 45° and 60° to the absorber, at a fixed separation of 100 mm and identical dimensions of 100 mm by 100 mm. As the relative angle increases, the transfer factors $f_{\mathrm{emi}\to\mathrm{abs}}$ and $f_{\mathrm{abs}\to\mathrm{emi}}$ of System I decrease from 0.20 to 0.11 and 0.08, respectively, leading to efficiencies of 8.7%, 3.0%, and 1.7% (Fig. R16b). At a relative angle of 60°, System II increases the transfer factor $f_{\mathrm{emi}\to\mathrm{abs}}$ from 0.08 to 1, thereby improving efficiency from 1.7% to 21.2%, while System III reaches the maximum theoretical efficiency of 100% by optimizing both $f_{\mathrm{emi}\to\mathrm{abs}}$ and $f_{\mathrm{abs}\to\mathrm{emi}}$.

Lateral misalignment

Third, we consider a face-to-face configuration with lateral misalignments L of 0, 100, and 200 mm, with both emitter and absorber having dimensions of 100 mm by 100 mm. As the lateral misalignment increases, the transfer factors $f_{\mathrm{emi}\to\mathrm{abs}}$ and $f_{\mathrm{abs}\to\mathrm{emi}}$ of System I decrease from 0.20 to 0.09 and 0.02, respectively, leading to efficiencies of 8.7%, 2.1%, and 0.1% (Fig. R16c). At a lateral misalignment of 200 mm, System II enhances the transfer factor $f_{\mathrm{emi}\to\mathrm{abs}}$ to 1, increasing the efficiency from 0.1% to 5.9%, while System III consistently maintains the theoretical maximum efficiency of 100%.

Overall, these results highlight that Systems II and III mitigate the sensitivity to geometric variations through directional thermal emission, demonstrating the geometric robustness and broad applicability of our framework.

We have clarified these points in the revised manuscript and included a brief discussion of the dependence on key geometric parameters.

We have made the below revisions in our revised manuscript.

***Revision*:**

Main text:

Line 161 - 164 on page 5:

Notably, in practical radiative heat transfer scenarios, despite constraints such as parasitic heat transfer losses (Supplementary Fig. 3) and geometric configurations (Supplementary Note 3 and Supplementary Fig. 4), System III consistently achieves the highest efficiency.

Supplementary Information:

Supplementary Note 3: Radiative heat transfer efficiency η under different geometric configurations

To clarify the impact of geometric factors on radiative exchange, we have analyzed the dependence of radiative heat transfer efficiency η on several key parameters, including separation distance, relative orientation, and lateral misalignment.

First, we consider an emitter placed face-to-face with an absorber, each with dimensions of 100 mm by 100 mm. As the separation distance D increases from 100 to 150 and 200 mm, the corresponding transfer factors $f_{\mathrm{emi}\to\mathrm{abs}}$ and $f_{\mathrm{abs}\to\mathrm{emi}}$ of System I decrease from 0.20 to 0.11 and 0.07, respectively, leading to efficiencies of 8.7%, 3.0%, and 1.3% (Supplementary Fig. 4a). This clearly indicates that, in conventional blackbody systems, efficiency is strongly limited by separation distances and decreases rapidly as the distance increases. In contrast, at a separation distance of 200 mm, System II optimizes the directional features of the emitter according to the geometric configuration, increasing the transfer factor $f_{\mathrm{emi}\to\mathrm{abs}}$ from 0.07 to 1 and thereby improving efficiency from 1.3% to 18.9%. System III further achieves the theoretical upper limit of 100% efficiency by simultaneously optimizing both $f_{\mathrm{emi}\to\mathrm{abs}}$ and $f_{\mathrm{abs}\to\mathrm{emi}}$.

Second, we consider an emitter with a relative angle β of 0°, 45° and 60° to the absorber, at a fixed separation of 100 mm and identical dimensions of 100 mm by 100 mm. As the relative angle increases, the transfer factors $f_{\mathrm{emi}\to\mathrm{abs}}$ and $f_{\mathrm{abs}\to\mathrm{emi}}$ of System I decrease from 0.20 to 0.11 and 0.08, respectively, leading to efficiencies of 8.7%, 3.0%, and 1.7% (Supplementary Fig. 4b). At a relative angle of 60°, System II increases the transfer factor $f_{\mathrm{emi}\to\mathrm{abs}}$ from 0.08 to 1, thereby improving efficiency from 1.7% to 21.2%, while System III reaches the maximum theoretical efficiency of 100% by optimizing both $f_{\mathrm{emi}\to\mathrm{abs}}$ and $f_{\mathrm{abs}\to\mathrm{emi}}$.

Third, we consider a face-to-face configuration with lateral misalignments L of 0, 100, and 200 mm, with both emitter and absorber having dimensions of 100 mm by 100 mm. As the lateral misalignment increases, the transfer factors $f_{\mathrm{emi}\to\mathrm{abs}}$ and $f_{\mathrm{abs}\to\mathrm{emi}}$ of System I decrease from 0.20 to 0.09 and 0.02, respectively, leading to efficiencies of 8.7%, 2.1%, and 0.1% (Supplementary Fig. 4c). At a lateral misalignment of 200 mm, System II enhances the transfer factor $f_{\mathrm{emi}\to\mathrm{abs}}$ to 1, increasing the efficiency from

0.1% to 5.9%, while System III consistently maintains the theoretical maximum efficiency of 100%. Overall, these results highlight that Systems II and III mitigate the sensitivity to geometric variations through directional thermal emission, demonstrating the geometric robustness and broad applicability of our framework.

Supplementary Figure 4:

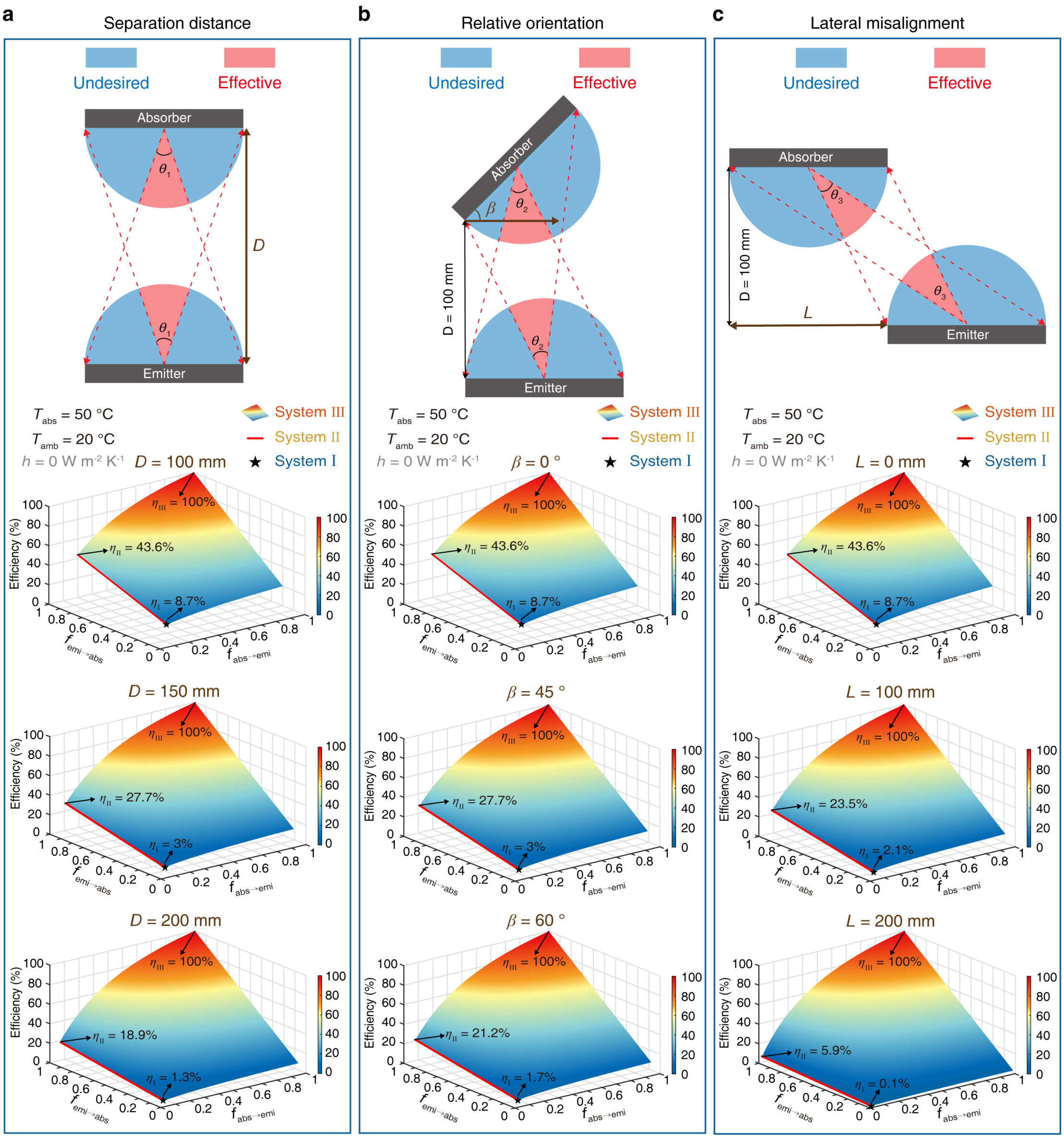

Supplementary Fig. 4. Theoretical radiative heat transfer efficiency η under different geometric configurations. a, Schematic of two surfaces (100 mm by 100 mm) placed face-to-face with a separation distance of D. The theoretical radiative heat transfer efficiencies of the three systems at different separation distances are shown below. **b,** Schematic of two surfaces (100 mm by 100 mm) oriented with a relative angle β at a fixed separation distance of 100 mm. The theoretical radiative heat transfer efficiencies of the three systems at different relative angles are shown below. **c,** Schematic of two surfaces (100 mm by 100 mm) with lateral misalignment L at a fixed separation distance of 100 mm. The theoretical radiative heat transfer efficiencies of the three systems at different lateral misalignments are shown below.

Comment #3: The theoretical comparison in Fig. 1 assumes a transfer factor of approximately 0.42 for blackbody-based systems, corresponding to a specific close-range configuration. In practical applications, however, the transfer factor can be significantly smaller due to larger separations or more complex geometries. The manuscript should clarify how the efficiency advantage of System III evolves as f decreases. Under a fixed heating requirement, it is not clear whether the magnitude of power savings remains substantial when f becomes much smaller than the illustrative value used in Fig. 1. This issue is also relevant to the experimental configurations in Figs. 2 and 3, where emitter and absorber are placed in close proximity. It should be clarified whether short separation distances are an inherent requirement of the proposed approach. A brief parametric trend showing efficiency enhancement as a function of f would clearly define the applicable range of the directional strategy, particularly for far-field or large-scale scenarios.

Response: We appreciate the reviewer's comment. Short separation distances are not an inherent requirement for directional radiative heat transfer. As discussed in our response to Comment #2, increasing the separation distance or introducing more complex geometric configurations could significantly reduce the efficiency of System I. In contrast, Systems II and III can enhance the transfer factor f by optimizing directional emission features, thereby improving radiative heat transfer efficiency.

Notably, when the directional emission features are fixed, complex geometric configurations become limiting factors for efficiency. To demonstrate this point, we consider the directional emitter of scenario 1 under a far-field configuration, where an emitter (100 mm by 100 mm) is placed horizontally, while an absorber of the same size is positioned vertically at distances of 50 mm and 100 mm from its edge (Fig. R17a). We calculate the corresponding efficiency and power consumption as shown in Fig. R17b. When the absorbers are heated to approximately 36 ºC in a vacuum environment, System III achieves efficiencies of 5.4% and 1.2% at separation distances of 50 mm and 100 mm, respectively, compared with 2.6% and 0.5% for System I. Correspondingly, System III reduces power consumption by approximately 56% and 55% relative to System I. Although the efficiency decreases and the power consumption increases for all three systems with increasing separation distance, Systems II and III consistently maintain higher efficiency and significant energy savings compared with System I. Further improvements can be achieved by optimizing the directional features according to the specific geometric configuration. Overall, directional thermal emission mitigates geometric constraints and enables efficient heat transfer across a wide range of geometric configurations.

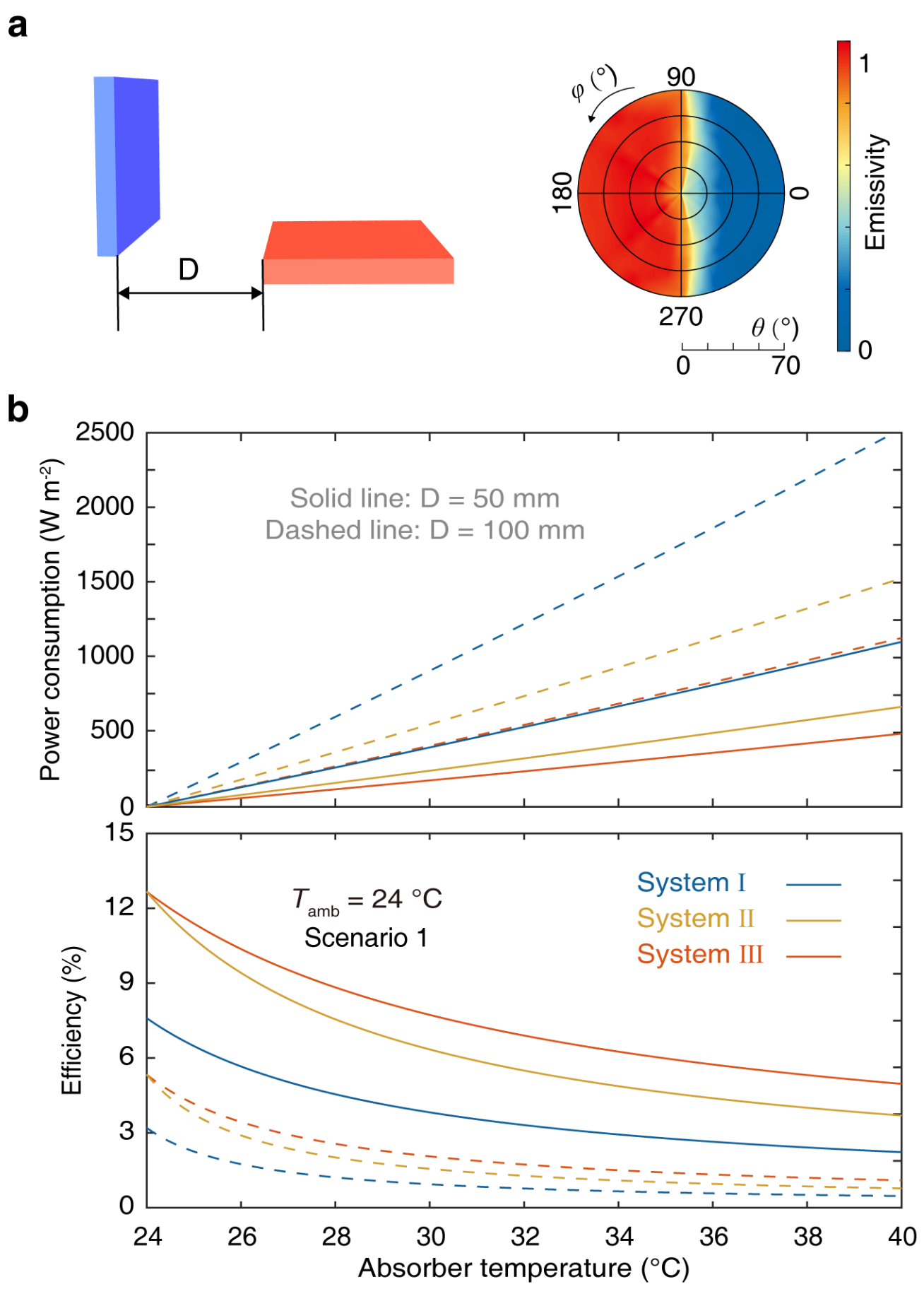

Fig. R17. Impact of separation distance on power consumption and efficiency for scenario 1 in a vacuum environment. a, Schematic of the geometric configuration (left) and the angularly asymmetric features of the directional emitter (right). **b,** Power consumption and efficiency of three systems at separation distances of 50 mm (solid lines) and 100 mm (dashed lines) with an ambient temperature of 24 °C.

Comment #4: Line 92 – 104 on page 3: The manuscript emphasizes that the transfer factor formulation generalizes conventional view-factor analysis and applies to systems with directional emission. However, the practical implementation of this formulation for complex or asymmetric geometries is not explained. The manuscript should clarify how the transfer factor would be evaluated in such cases. Providing guidance on computational methods, complexity, or expected accuracy would help readers assess whether the framework can be applied to realistic engineering systems.

Response: We appreciate the reviewer's comment. The transfer factor f is applicable to arbitrarily geometric configurations, thereby providing a flexible and practical framework for quantifying radiative heat transfer in realistic engineering systems, including those with complex and asymmetric geometries. In our analysis, radiative exchange between two surfaces is evaluated by integrating over differential surface elements, while accounting for their angular and spectral emissivity/absorptivity (Fig. R18). Below, we provide a brief discussion of the computational methods, complexity,

and expected accuracy:

The transfer factor $f_{\mathrm{emi}\to\mathrm{abs}}$ can be expressed as:

$$f_{\mathrm{emi}\to\mathrm{abs}}=\frac{\int_0^\infty d\lambda\int_{A_{\mathrm{emi}}}\int_{A_{\mathrm{abs}}}I_{\mathrm{BB}}(T_{\mathrm{emi}},\lambda)\varepsilon_{\mathrm{emi}}(\theta_{\mathrm{emi}},\varphi_{\mathrm{emi}},r_{\mathrm{emi}},\lambda)\alpha_{\mathrm{abs}}(\theta_{\mathrm{abs}},\varphi_{\mathrm{abs}},r_{\mathrm{abs}},\lambda)\frac{\cos\theta_{\mathrm{emi}}\cos\theta_{\mathrm{abs}}}{L^2}dA_{\mathrm{abs}}dA_{\mathrm{emi}}}{\int_{A_{\mathrm{emi}}}dA_{\mathrm{emi}}\int_0^\infty I_{\mathrm{BB}}(T_{\mathrm{emi}},\lambda)d\lambda\int_\Omega\varepsilon_{\mathrm{emi}}(\theta_{\mathrm{emi}},\varphi_{\mathrm{emi}},r_{\mathrm{emi}},\lambda)\cos\theta_{\mathrm{emi}}\,d\Omega}$$

In our framework, the emitter and absorber surfaces are first discretized into infinitesimal surface elements dA_{emi} and dA_{abs} according to the practical geometric configurations. A mapping relationship is then established between all interacting surface elements of the two surfaces. The angular and spectral emissivity/absorptivity distributions of the interacting surface elements are then input into the calculation to determine the radiative exchange between them. Subsequently, the contributions from all surface elements are integrated over angle, wavelength and surface area to obtain the total radiative exchange between the two surfaces. Finally, the transfer factor is determined by normalizing the emissive power transferred from the emitter to the absorber with respect to the total emitted power from the emitter.

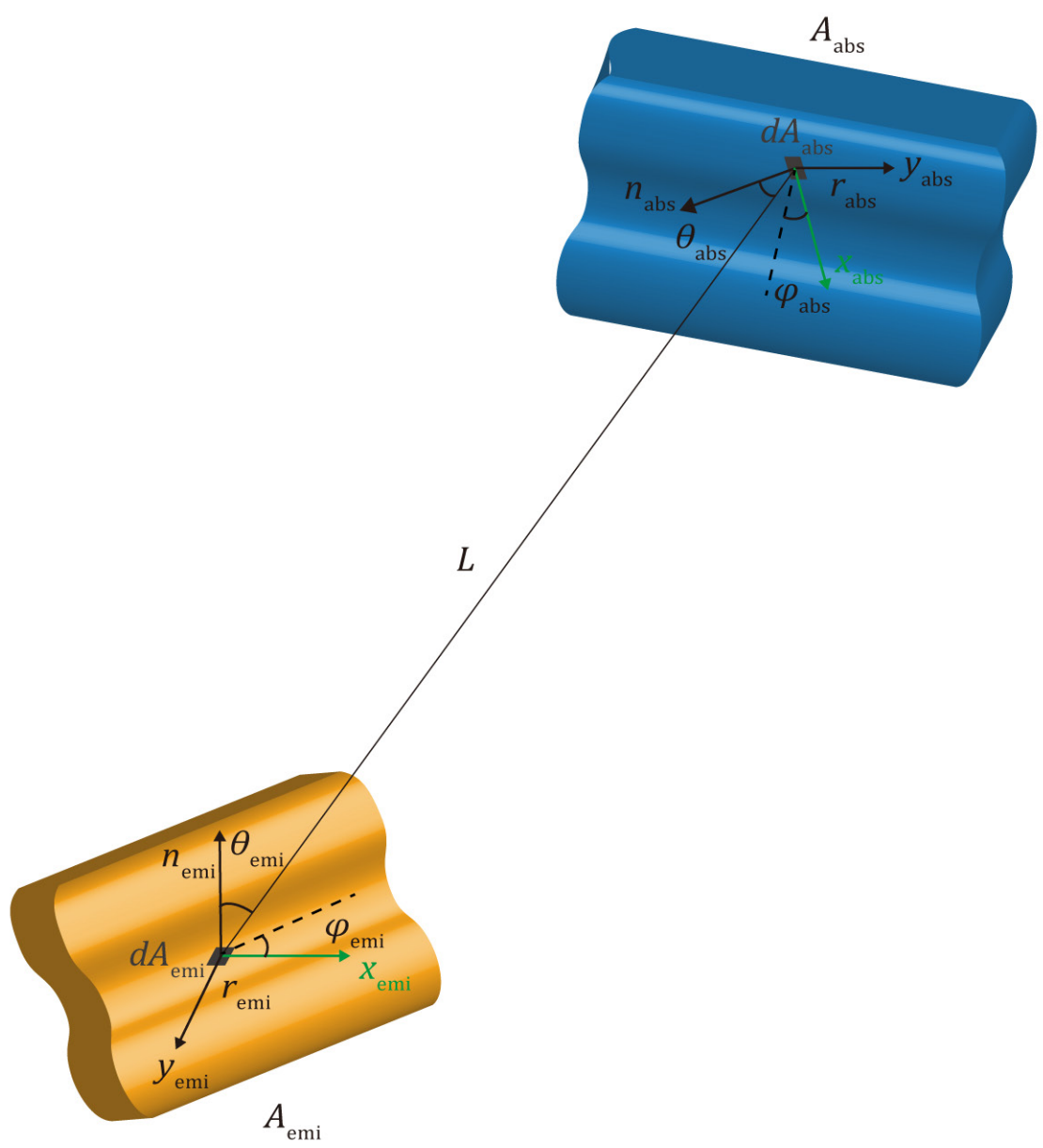

Fig. R18. Schematic of radiative exchange between two complex surfaces. Surface elements dA_{emi} and dA_{abs} can be discretized into infinitesimal surface elements.

The transfer factor can be evaluated using standard numerical integration approaches, such as the Trapezoidal rule, Simpson's rule and Gaussian quadrature schemes. As the geometric configuration and directional emission features become increasingly complex, the computational cost correspondingly increases due to the larger number of surface element interactions that must be evaluated. In practice, the computational accuracy mainly depends on the discretization resolution and the selected numerical integration scheme. Converged solutions can be obtained by progressively refining the

surface discretization and angular/spectral sampling until the calculated transfer factor exhibits negligible variation. By sufficiently refining the surface discretization and angular/spectral sampling to accurately capture the geometric configuration and directional emission features, high computational accuracy can be achieved for realistic engineering systems.

We have made the below revisions in our revised manuscript.

Revision:

Supplementary Information:

Supplementary Note 1:

Line 44 - 48 on page 2:

The transfer factor is applicable to arbitrary geometric configurations by discretizing complex surfaces into differential surface elements and numerically integrating the angular and spectral radiative exchange between all interacting elements, enabling accurate evaluation of radiative heat transfer in realistic engineering systems.

Comment #5: Page 6: Figure 2 presents vacuum experiments demonstrating directional radiative heat transfer. While the schematic is clear, the experimental description lacks sufficient detail for reproducibility. Photographs of the vacuum setup and a concise description of key parameters should be provided in the Supporting Information. These should include sample mounting, alignment tolerances, temperature sensor placement, vacuum level stability, and radiation shielding configuration. Such information is necessary for evaluating parasitic effects and reproducing the reported results.

Response: We appreciate the reviewer's comment. The key parameters of vacuum setup have been described in the Methods and Supplementary Note 4. To further enhance the clarity of the experimental setup, we have included a photograph of the vacuum setup (Fig. R19) and more detailed description of key parameters in Methods and Supplementary Note 4, including sample mounting, alignment tolerances, temperature sensor placement, vacuum level stability, and radiation shielding configuration.

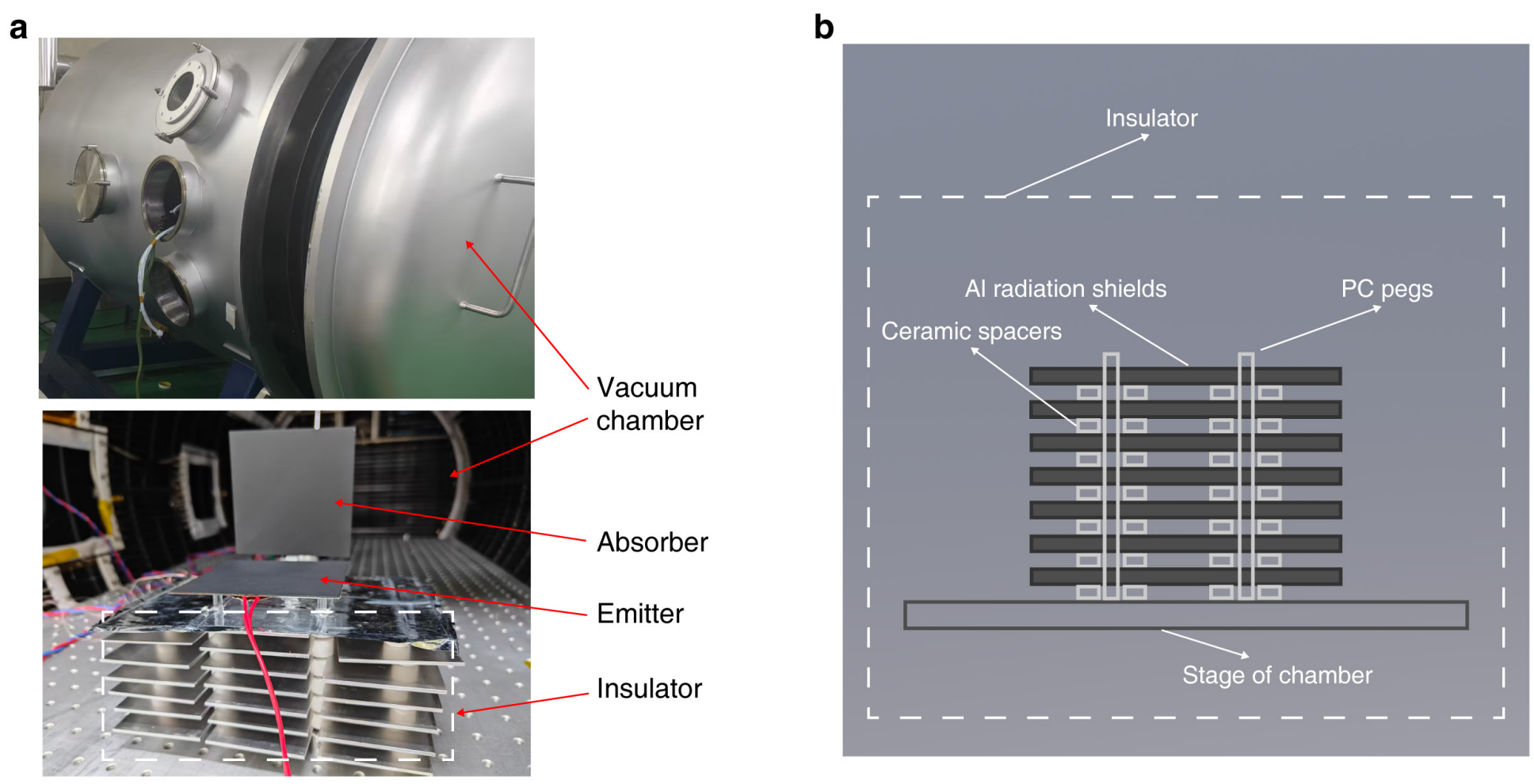

Fig. R19. Photographs of the vacuum set-up and schematic of the thermal insulation device. a, Photographs of the vacuum chamber and experimental set-up. The vacuum level was kept at approximately 6×10^{-6} Torr. **b,** The thermal insulation device consists of four PC pegs and seven aluminum radiation shields, designed to minimize heat losses from radiation and conduction.

In the first scenario, an emitter (100 mm by 100 mm) is placed horizontally, while an absorber of the same size is positioned vertically, 10 mm from the edge of the emitter. In the second scenario, an emitter (30 mm by 30 mm) is placed horizontally, with a parallel absorber of the same size positioned 30 mm above it. The positions of all components were carefully aligned according to the geometric configurations, with precise measurements performed to minimize alignment errors. Polyimide resistance heaters were attached to the backs of the emitters for providing heating power. The emitters are placed at the top of the thermal insulation device, in contact with the four PC pegs (5 mm in diameter), to minimize heat conduction losses. Thermocouples are fixed at the center of both the emitter and absorber backs, covered with aluminum foil to prevent radiative heat loss. A 3 mm thick acrylic plate is fixed to the back of the absorber to provide support.

The vacuum chamber has a diameter of 1.2 meters, with its inner surface coated in high-emissivity paint. The operating vacuum level can be maintained below 6×10^{-6} Torr. Thermal insulation devices are placed on a metal stage inside the chamber, each consisting of four polycarbonate (PC) pegs and seven radiation shields. We used aluminum plates with an infrared emissivity of approximately 0.07 for the radiation shields. Ceramic spacers with a height of 10 mm are inserted between the radiation shields to provide structural support and maintain their separation.

We have made the below revisions in our revised manuscript.

Revision:

Main text:

Methods

Directional radiative heat transfer tests

Line 518 - 520 on page 19:

The vacuum chamber has a diameter of 1.2 meters, with its inner surface coated in high-emissivity paint. The vacuum level was kept at approximately 6×10^{-6} Torr and the ambient temperature was maintained at 24 °C.

Line 527 - 528 on page 19:

Thermocouples were fixed at the center of both the emitter and absorber backs, covered with aluminum foil to prevent radiative heat loss.

Supplementary Information:

Supplementary Note 4:

Line 160 - 165 on page 6:

The positions of all components were carefully aligned according to the geometric configurations, with precise measurements performed to minimize alignment errors. Aluminum foil is applied to the back of the emitter to reduce radiative loss. In addition, seven concentric aluminum radiation shields are placed between the emitter and the vacuum chamber floor, supported by ceramic spacers (10 mm in height).

Supplementary Figure 20:

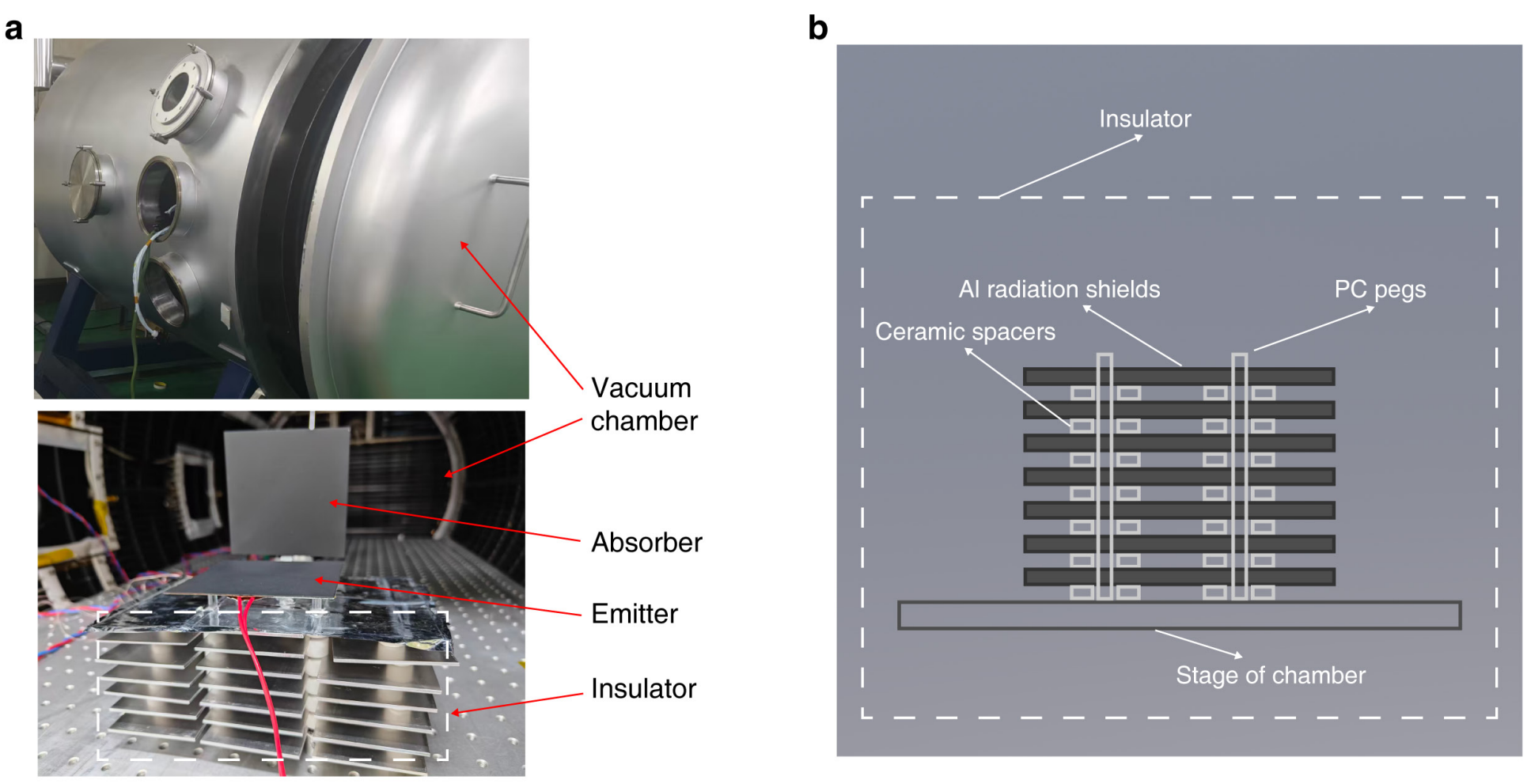

Supplementary Fig. 20. Photographs of the vacuum set-up and schematic of the thermal insulation device. a, Photographs of the vacuum chamber and experimental set-up. The vacuum level was kept at approximately 6×10^{-6} Torr. **b,** The thermal insulation device consists of four PC pegs and seven aluminum radiation shields, designed to minimize heat losses from radiation and conduction.

Comment #6: Page 8: In the non-vacuum indoor experiments shown in Fig. 3, convection and conduction contribute to heat transfer in addition to radiation. Although

parasitic heat transfer coefficients are included in the model, the relative contributions of radiation, convection, and conduction are not quantified. A quantitative decomposition based on the existing model parameters should be provided. This analysis is necessary to demonstrate whether directional radiation remains the dominant mechanism under realistic indoor conditions.

Response: We appreciate the reviewer's comment. Below we provide analysis to show that directional radiation remains the dominant mechanism under realistic indoor conditions.

To quantitatively assess the relative contributions of radiation, convection, and conduction, we have performed a quantitative decomposition based on the thermal balance of both the emitter and absorber.

Specifically, the total power consumption of the emitter $P_{\text{consum}}(T_{\text{emi}})$ is expressed as:

$$P_{\text{consum}}(T_{\text{emi}}) = P_{\text{rad}}(T_{\text{emi}}) + P_{\text{conv}}(T_{\text{emi}}) + P_{\text{cond}}(T_{\text{emi}}) \quad \text{(R1)}$$

where P_{rad}, P_{conv}, and P_{cond} denote the contributions from radiation, convection, and conduction, respectively. Similarly, the thermal balance equation of the absorber is given by:

$$P_{\text{net}}(T_{\text{abs}}) = P_{\text{rad}}(T_{\text{abs}}) + P_{\text{conv}}(T_{\text{abs}}) + P_{\text{cond}}(T_{\text{abs}}) \quad \text{(R2)}$$

Thermal equilibrium is achieved when the net power $P_{\text{net}} = 0$. The net radiative power input to the absorber is given by:

$$P_{\text{rad}}(T_{\text{abs}}) = Q_{\text{abs-emi}}(T_{\text{abs}}) + Q_{\text{abs-bg}}(T_{\text{abs}}) \quad \text{(R3)}$$

where $Q_{\text{abs-emi}}(T_{\text{abs}})$ is the net radiative heat flux from the absorber to the emitter, and $Q_{\text{abs-bg}}(T_{\text{abs}})$ is the net radiative heat flux from the absorber to the background.

Relative contributions to power consumption

By simultaneously solving equation R1 and R2, we have quantified the relative contributions of radiation, convection, and conduction for power consumption. As shown in Fig. R20, compared with System I, Systems II and III utilize directional thermal emission to effectively suppress radiative losses, thereby achieving efficient radiative heat transfer. These calculations were performed under the indoor condition that the absorber temperature is approximately 26 °C at an ambient temperature of 21 °C, assuming a convection coefficient of $h_{\text{conv}} = 7\ \text{W m}^{-2}\text{K}^{-1}$.

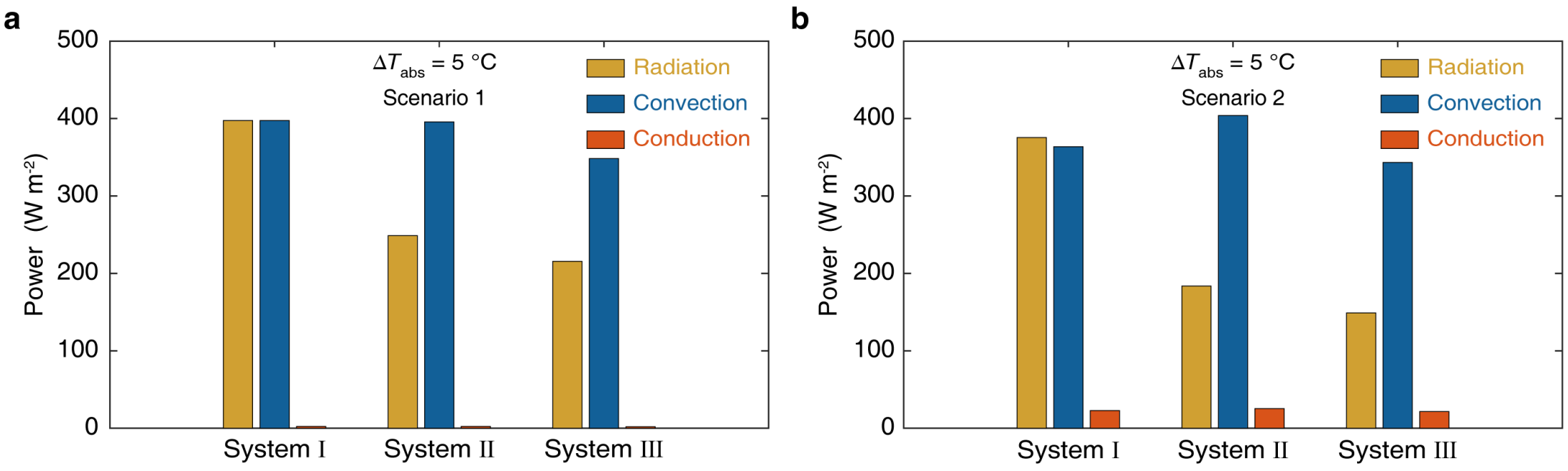

Fig. R20. Relative contributions of radiation, convection, and conduction to power consumption. a,b, Relative contributions to power consumption of the emitter in scenario 1 (**a**) and scenario 2 (**b**). Compared with System I, Systems II and III utilize directional thermal emission to effectively suppress radiative losses, thereby achieving efficient radiative heat transfer. The calculations were performed under indoor conditions with an absorber temperature of 26 ºC and an ambient temperature of 21 °C.

Thermal balance analysis of the absorber

For the absorber, thermal radiation serves as the primary net heat input in all three systems (Fig. R21). The net radiative power received from emitter $Q_{\mathrm{abs-emi}}(T_{\mathrm{abs}})$ is balanced by radiative exchange with the background $Q_{\mathrm{abs-bg}}(T_{\mathrm{abs}})$, as well as convective loss $P_{\mathrm{conv}}(T_{\mathrm{abs}})$ and conductive loss $P_{\mathrm{cond}}(T_{\mathrm{abs}})$. These results indicate that convection and conduction serve as the dominant heat loss pathways. Notably, System III effectively suppress radiative loss to the background, thereby enhancing efficiency.

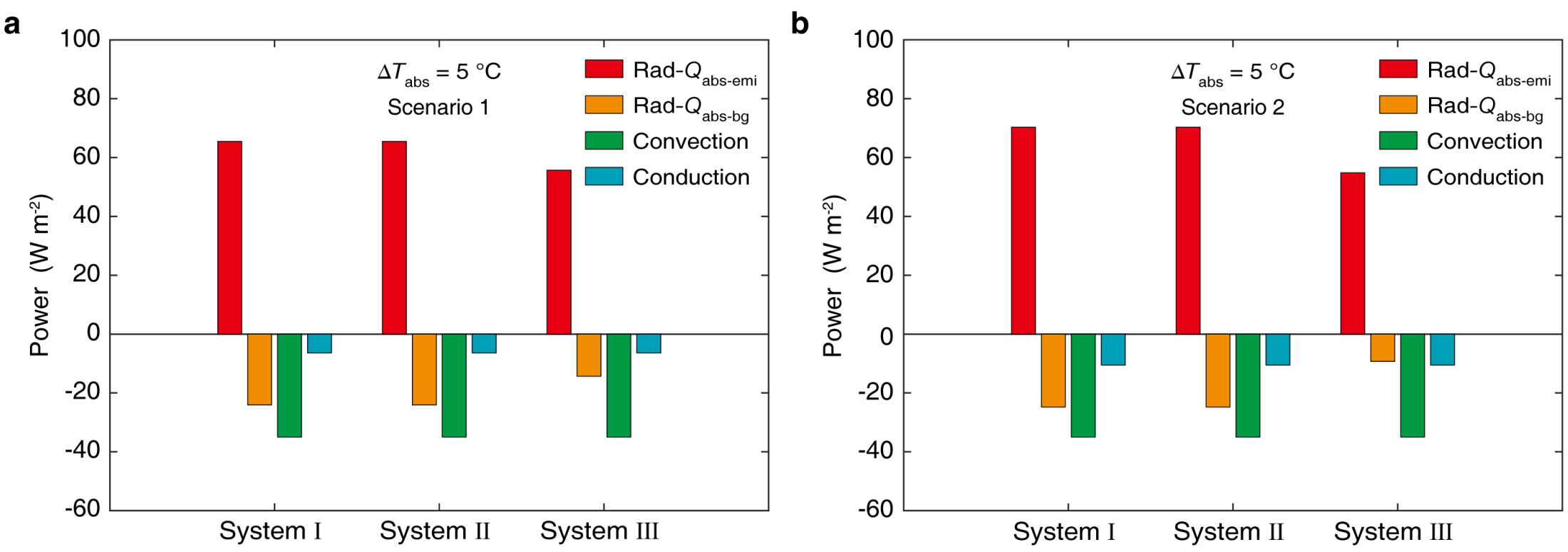

Fig. R21. Thermal balance analysis of the absorber in scenario 1 (a) and scenario 2 (b). Thermal radiation serves as the primary net heat input in all three systems, while convection and conduction act as the dominant heat loss pathways. The calculations were performed under indoor conditions with an absorber temperature of 26 ºC and an ambient temperature of 21 °C.

Comment #7: Page 10: The vehicle heating demonstration in Fig. 4 compares a directional emitter with a blackbody emitter. While this comparison isolates the effect of directional radiation, its engineering relevance requires clarification. In practical vehicles, cabin heating is primarily achieved through convective air heating, typically

using heat-pump-based HVAC systems. Moreover, the radiative heating configuration illustrated beneath Fig. 4b represents a heating mode that is uncommon in real vehicle applications, which limits the interpretability of the chosen reference. The manuscript should clearly define the intended role of directional radiative heating in vehicles, for example whether it is proposed as a primary heating mechanism or as a supplemental method for localized thermal comfort. The limitations of comparing with a blackbody emitter, rather than with widely used HVAC systems, should also be discussed.

Response: We appreciate the reviewer's comment. The vehicle heating demonstration is intended to illustrate the application potential of directional radiative heating. It is not proposed as a primary heating mechanism to replace existing HVAC systems, but rather as a complementary approach for providing localized thermal comfort which can reduce heating energy consumption. In addition, as electric vehicles (EVs) become increasingly widespread, radiative heating is being actively explored as an emerging approach for improving thermal management in EVs.

Unlike internal combustion engine (ICE) vehicles, which can utilize waste heat for cabin heating, EVs require substantial additional power for heating, thereby placing greater demands on efficient thermal management. Recent efforts by automotive manufacturers, such as Hyundai Motor Company, have explored radiative heating systems for vehicle cabin heating to improve thermal comfort (https://www.hyundaimotorgroup.com/en/story/CONT0000000000161147).

Our proposed directional radiative heating strategy offers a promising approach by selectively directing heat flux to the human body, thereby reducing energy consumption compared with heating the entire cabin. The integration of directional radiative heating with conventional HVAC systems also holds significant potential for further reducing energy loss and extending EV driving range. Therefore, the configuration shown in Fig. 4 of the main text is intended as a simplified and controlled scenario to isolate and evaluate the effect of directional radiation in vehicles, rather than to directly compare its performance with existing HVAC systems. We have clarified these points in the revised manuscript to improve the engineering relevance of the demonstration.

We have made the below revisions in our revised manuscript.

Revision:

Main text:

Line 271 - 278 on page 12:

As electric vehicles (EVs) become increasingly widespread, efficient cabin heating has become an important challenge because EVs lack the waste heat available in internal combustion engine (ICE) vehicles. Consequently, radiative heating is attracting growing interest as a potential strategy for improving heating efficiency and localized thermal comfort in EVs[51]. In this context, we further evaluate the potential of directional

radiative heat transfer for efficient vehicle heating using an environmental simulation chamber (ESC), which allows precise control of ambient temperature (-40 °C to 45 °C) and relative humidity (10% to 90%) (Fig. 4a).

Line 292 - 297 on page 12:

Our results highlight the potential of directional radiative heating in vehicle applications, enabling selectively warming of targeted regions without the need to heat the entire vehicle cabin. This approach can serve as a complement to conventional HVAC systems by providing localized thermal comfort and is emerging as a promising strategy to improve heating efficiency in electric vehicles.

Reference:

51. Hyundai Motor Group. Radiant Heating System: Advanced Thermal Management Technology for the Future of Mobility. https://www.hyundaimotorgroup.com/en/story/CONT0000000000161147

Comment #8: In the human heating scenario presented in Fig. 5, the local radiative heat flux incident on the target region reaches values on the order of 1000 W m⁻2. While the manuscript correctly emphasizes the localized nature of this heating, such flux levels may raise concerns regarding thermal comfort or safety if interpreted without context. How these flux values relate to accepted thermal comfort or exposure thresholds, and how to specify the spatial extent and typical exposure duration associated with the reported conditions?

Response: We appreciate the reviewer's comment. The safety associated with the incident radiative heat flux was carefully considered in the design. In the human heating scenario presented in Fig. 5 of the main text, under an ambient temperature of 0 °C and convection coefficients ranging from 2 to 8 W m^{-2} K^{-1}, the local radiative heat flux incident on the target region was estimated to be approximately 100-1000 W m^{-2} (Supplementary Fig. 15). This flux range was maintained below the maximum continuous radiant intensity of 1580 W m^{-2} considered tolerable for personnel wearing appropriate clothing, as specified in API Standard 521 (Table 12, https://www.researchgate.net/profile/Wang-Dian-3/publication/341279222_Pressure-relieving_and_Depressuring_Systems/data/5eb7f90fa6fdcc1f1dcb3ae5/API-Std-521-2014-6th-Pressure-relieving-and-Depressuring-Systems.pdf).

Notably, the human body heating analysis is intended as a proof-of-concept demonstration of directional radiative heating. In practical implementations, the local radiative heat flux can be readily tuned according to ambient conditions (e.g., ambient temperature and convection coefficient) by adjusting the emitter temperature, thereby ensuring both thermal comfort and safety. Furthermore, the spatial extent and exposure duration in real applications can be controlled through geometric design and system

operation, enabling localized, intermittent, or user-specific heating rather than continuous full-body exposure.

Comment #9: While the Discussion highlights the conceptual advantages of directional radiative heat transfer, the manuscript would benefit from a brief perspective on manufacturing and deployment considerations. Directional emitters often rely on photonic or metamaterial structures whose scalability, cost, and long-term stability may influence real-world adoption. A brief discussion should be added on large-area fabrication, integration with existing heating systems, and durability under thermal cycling. This perspective is necessary to place the work in a realistic engineering context.

Response: We appreciate the reviewer's comment. We agree that large-area fabrication, integration with existing heating systems, and durability under thermal cycling are crucial for the practical implementation of directional radiative heat transfer. Accordingly, we provide a brief discussion of these aspects below.

With ongoing advances in large-area nanofabrication and manufacturing technologies, directional radiative heat transfer is expected to become increasingly feasible for real-world applications. Significant progress has already been made in the fabrication and deployment of directional photonic structures, which provides a solid foundation for their potential real-world application. In terms of large-area fabrication, scalable approaches such as roll-to-roll printing [1-2] and industrial thin-film deposition techniques (https://www.agc-plasma.com/technologies/plasma-enhanced-chemical-vapor-deposition-pecvd, https://www.geomatec.com/special-feature/roll-to-roll/, https://www.leybold.com/en-us/applications-and-industries/coating/roll-to-roll-film-coating) have been widely demonstrated for producing micro/nanostructured and multilayer optical coatings over large substrates. These established manufacturing methods provide viable pathways to address scalability challenges associated with directional emitters. Based on these capabilities, directional emitters could be readily integrated with existing heating systems.

Additionally, long-term stability can be ensured through appropriate material selection. Many candidate materials, including dielectric multilayers, refractory metals, and metal oxides, exhibit excellent thermal and chemical stability and are widely used in high-temperature and thermally cycling environments [3-4], demonstrating strong resistance to operational degradation. With appropriate encapsulation, these emitters are expected to maintain long-term stability under practical operating conditions.

[1] Lin, K.-T. *et al*. Highly efficient flexible structured metasurface by roll-to-roll printing for diurnal radiative cooling. *eLight* **3**, 22 (2023).

[2] Jiang, Y. *et al*. Hierarchical design and scalable production of radiative cooling film featuring multispectral camouflage. *Nat. Commun.* **17**, 2253 (2026).

[3] Park, J. H., Han, S. E., Nagpal, P. & Norris, D. J. Observation of Thermal Beaming from Tungsten and Molybdenum Bull's Eyes. *ACS Photonics* **3**, 494–500 (2016).

[4] Zhu, H. *et al.* High-temperature infrared camouflage with efficient thermal management. *Light Sci. Appl.* **9**, 60 (2020).

We have made the below revisions in our revised manuscript.

Revision:

Main text:

Line 352 - 356 on page 14:

In particular, driven by advances in thermal radiation control technologies related to thermal cycling durability[56,57], large-area fabrication and integration[58,59], directional radiative heat transfer is expected to become increasingly feasible for real-world deployment, offering a pathway toward highly efficient energy transfer and contributing to global carbon neutrality and sustainability.

Reference:

56. Park, J. H., Han, S. E., Nagpal, P. & Norris, D. J. Observation of Thermal Beaming from Tungsten and Molybdenum Bull's Eyes. *ACS Photonics* **3**, 494–500 (2016).

57. Zhu, H. *et al.* High-temperature infrared camouflage with efficient thermal management. *Light Sci. Appl.* **9**, 60 (2020).

58. Lin, K.-T. *et al.* Highly efficient flexible structured metasurface by roll-to-roll printing for diurnal radiative cooling. *eLight* **3**, 22 (2023).

59. Jiang, Y. *et al.* Hierarchical design and scalable production of radiative cooling film featuring multispectral camouflage. *Nat. Commun.* **17**, 2253 (2026).

In summary, the work provides a systematic analysis of directional radiative heat transfer, linking theoretical modeling with experimental validation in realistic environments. The results help clarify the conditions under which directional emission can improve energy efficiency, which is of interest to the thermal management and energy community.

Response: We extend our deep gratitude once again to the reviewer for recognizing the systematic analysis and significance of our work, as well as for providing constructive suggestions. All comments have been carefully addressed, with point-by-point revisions and responses provided as above. We hope the reviewer will find our responses satisfactory.

Response to Reviewer #3:

Reviewer #3: I co-reviewed this manuscript with one of the reviewers who provided the listed reports. This is part of the Nature Communications initiative to facilitate training in peer review and to provide appropriate recognition for Early Career Researchers who co-review manuscripts.

Response: We sincerely appreciate the reviewer for participating in the co-review process and contributing to the evaluation of our manuscript, as well as for the reviewer's time and effort in supporting the peer review process.

Response to Reviewer #4:

Reviewer #4: This manuscript presents a well-designed and experimentally validated study on directional radiative heat transfer. The introduction of the transfer factor provides a meaningful extension beyond the conventional view factor framework, and the combination of theoretical analysis and practical demonstrations convincingly shows substantial energy savings in targeted heating applications. The reduction in emitter temperature together with decreased power consumption further highlights the practical significance of the work.

Response: We sincerely appreciate the reviewer for recognizing the practical significance of our work. We also appreciate the reviewer's insightful suggestion which we address as below.

However, I encourage the authors to clarify the thermodynamic interpretation of the reported efficiency enhancement. In many demonstrated cases, the improvement primarily results from suppressing radiative losses to undesired surroundings and concentrating heat onto a specific target. In this sense, the mechanism may be better interpreted as spatial heat relocation rather than an increase in net heat extraction capability. A brief discussion distinguishing directional heat focusing from true net heat removal to an external reservoir would improve conceptual clarity and position the work more precisely within the broader context of radiative thermal management.

Response: We appreciate the reviewer's comment. We agree that the mechanism of directional radiative heat transfer is better interpreted as spatial heat relocation, where heat is concentrated in the target area by directing the heat flux, thereby minimizing unwanted heat loss. We have included a brief discussion in the manuscript that distinguishes directional radiative heat transfer from true net heat removal to an external reservoir.

We have made the below revisions in our revised manuscript.

Revision:

Main text:

Line 145 - 152 on page 5:

When both the emitter and absorber exhibit perfectly matched directional features, $f_{\text{emi}\rightarrow\text{abs}}$ and $f_{\text{abs}\rightarrow\text{emi}}$ can be optimized to unity, enabling thermal radiation to be directed precisely toward the target region while minimizing radiative losses to undesired directions. Consequently, System III reaches the theoretical upper efficiency limit of $\eta_{\text{III}} = 100\%$. In this case, the efficiency of System III becomes independent of the absorber temperature (Fig. 1c), whereas the efficiencies of Systems I and II decrease significantly at large temperature differences between the absorber (or emitter) and the background.

Line 324 - 326 on page 14:

In contrast, the directional emitter primarily directs radiative flux toward the paint, achieving 22-35% energy savings compared with the blackbody emitter (Fig. 5c).

Line 342 - 344 on page 14:

our approach precisely directs heat flux to the target area while minimizing energy dissipation in undesired directions.

We once again deliver our deepest gratitude to the reviewer for recognizing the practical significance of our work and providing this insightful suggestion. We hope the reviewer will find our response satisfactory.

Response to Reviewer #5:

Reviewer #5: In this work, the authors demonstrate that directional thermal emission can significantly enhance radiative heat transfer efficiency by reducing unwanted radiative losses into undesired directions. They introduce a transfer-factor framework to quantify efficiency in directional emitter–absorber systems and show that perfectly matched angular emission and absorption can theoretically yield 100% efficiency. Experimental demonstrations confirm substantial power savings compared with blackbody emitters, and several application scenarios illustrate potential practical benefits. Overall, the work is interesting and could merit publication in Nature Communications. However, several important issues must be addressed and clarified before it can be considered for publication.

Response: We express our gratitude to the reviewer for recognizing the potential practical benefits of our work. We also appreciate the reviewer's insightful comments and constructive suggestions which we address point by point as below.

Comment #1: Major issues:

The physical mechanism underlying the thermal radiation directivity is not described in sufficient depth. Directionality is treated largely as an imposed or effective property through angular emissivity profiles, without a clear electromagnetic explanation of how the proposed structures generate directional emission. Key wave-physics mechanisms—such as interference effects, resonant mode selection, momentum filtering, or anisotropic dispersion—are not rigorously analyzed, which limits physical insight. This aspect should constitute the core of the manuscript.

Response: We appreciate the reviewer's comment. We fully agree that key wave-physics mechanisms, such as interference effects, resonant mode selection, momentum filtering, and anisotropic dispersion, play a fundamental role in enabling directional thermal emission. The primary objective of our work, however, is to demonstrate that directional control of thermal radiation can enable highly efficient radiative heat transfer, rather than provide a comprehensive analysis of the electromagnetic mechanisms underlying specific emitter designs. Moreover, the directional emitters employed in our experiments are based on previously reported designs (https://doi.org/10.1126/science.adn2524, https://doi.org/10.1038/s41467-024-48826-9), where the underlying physical mechanisms have been thoroughly analyzed.

The directional emitter in scenario 1, based on our previous work (https://doi.org/10.1126/science.adn2524), exhibits angularly asymmetric emission arising from a sawtooth grating with broken in-plane mirror symmetry. In this design, the Ag layer on the slanted surfaces suppresses emission over the angular range of 0° to 70°, while emission from -70° to 0° is primarily contributed by the high-emissivity substrate (PDMS) (Fig. 2b in the main text). In scenario 2, the directional emitter follows the design reported in previous work (https://doi.org/10.1038/s41467-024-

48826-9), where thermal emission from the bottom PDMS is collimated by Ag reflectors and redistributed into a designed angular range of -40° to 40° (Fig. 2c in the main text).

In response to the reviewer's comment, a brief discussion of the directional emitters has been added to Supplementary Figure 5.

We have made the below revisions in our revised manuscript.

Revision:

Supplementary Information:

Supplementary Figure 5:

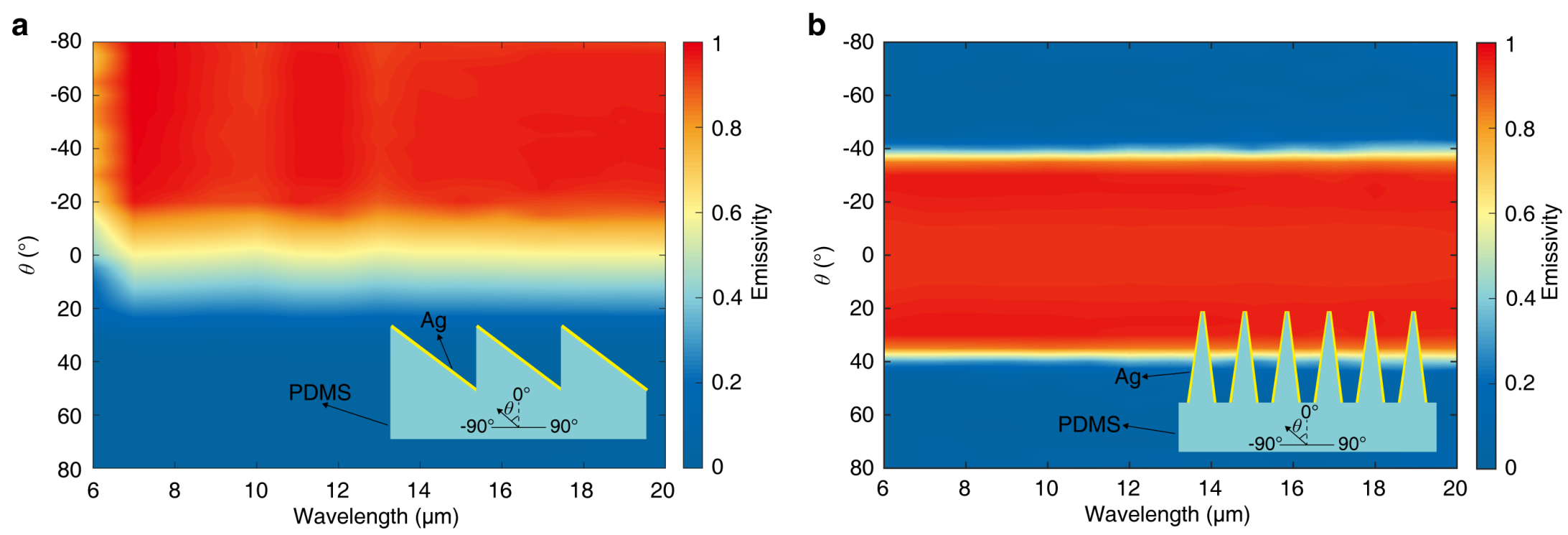

Supplementary Fig. 5. Simulated spectral angular emissivity of directional emitters in scenario 1 and 2. a, The directional emitter in scenario 1 exhibits angularly asymmetric emission arising from a sawtooth grating with broken in-plane mirror symmetry. In this design, the Ag layer on the slanted surfaces suppresses emission over the angular range of 0° to 80°, while emission from -80° to 0° is primarily contributed by the high-emissivity substrate (PDMS). **b,** The directional emitter exhibits high emissivity over the angular range of -40° to 40° based on a periodic conical structure. In this design, thermal emission from the bottom PDMS is collimated by Ag reflectors and redistributed into the designed angular range.

Comment #2: In addition, the materials and emitter structuration are mainly characterized through effective emissivity or absorptivity. The roles of frequency-dependent dielectric functions, material losses, dispersion, temperature dependence, and fabrication tolerances are not systematically discussed. As a result, the constraints imposed by real materials on the achievable directionality and efficiency remain unclear.

Response: We appreciate the reviewer's comment. We agree that material properties, such as frequency-dependent dielectric functions, losses, dispersion, temperature dependence, and fabrication tolerances, are important factors in determining the achievable emission features. As discussed in response to Comment #1, the primary objective of this work is to establish a general framework demonstrating that directional

control of thermal radiation can enable highly efficient radiative heat transfer, rather than to provide a detailed analysis of specific emitter implementations.

To facilitate understanding of the material optical properties, we provide the permittivity of the two primary materials used in the directional emitters (Fig. R22). Ag serves as the reflective layer with negligible infrared absorptivity, while PDMS exhibits strong infrared absorption.

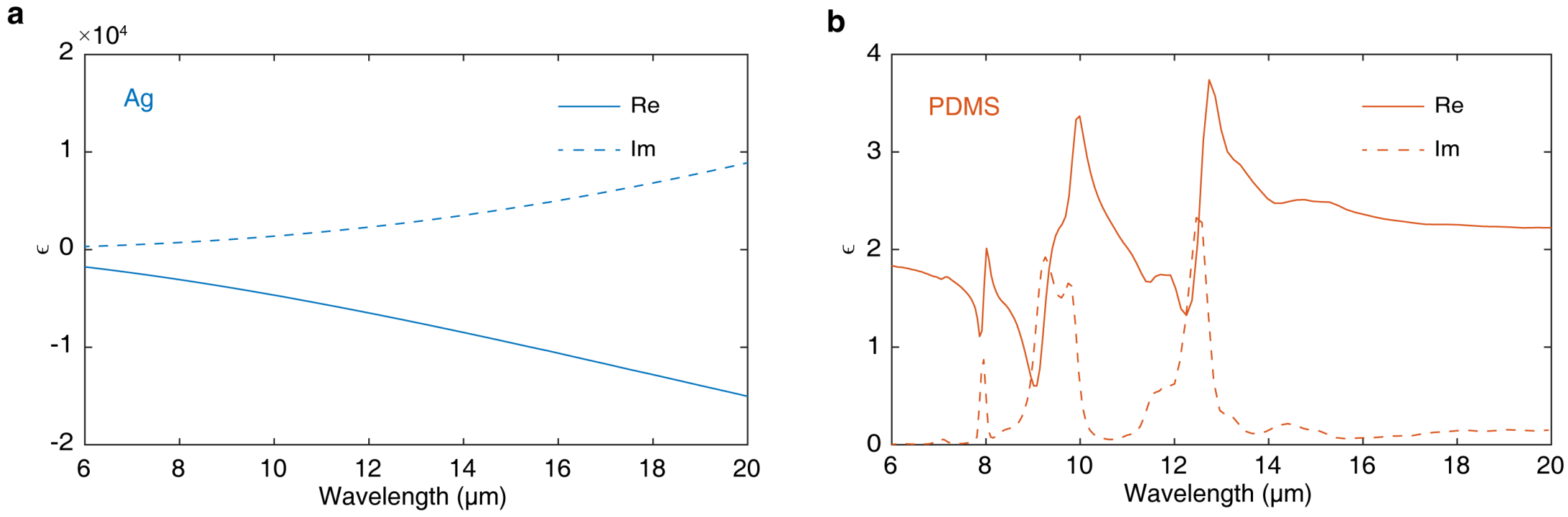

Fig. R22. Permittivity of Ag and PDMS. a,b, Real (solid) and imaginary (dashed) parts of the permittivity of Ag (**a**) and PDMS (**b**) in the infrared wavelength range. Ag exhibits negligible absorptivity in the infrared range, whereas PDMS shows strong infrared absorption.

Both the emitter and absorber operate at approximately 20-100 °C and exhibit high thermal stability within this temperature range. Moreover, since the structural periods are much larger than the operating wavelength range (6-20 μm), the directional response is robust against fabrication imperfections.

We have made the below revisions in our revised manuscript.

Revision:

Supplementary Information:

Supplementary Figure 6:

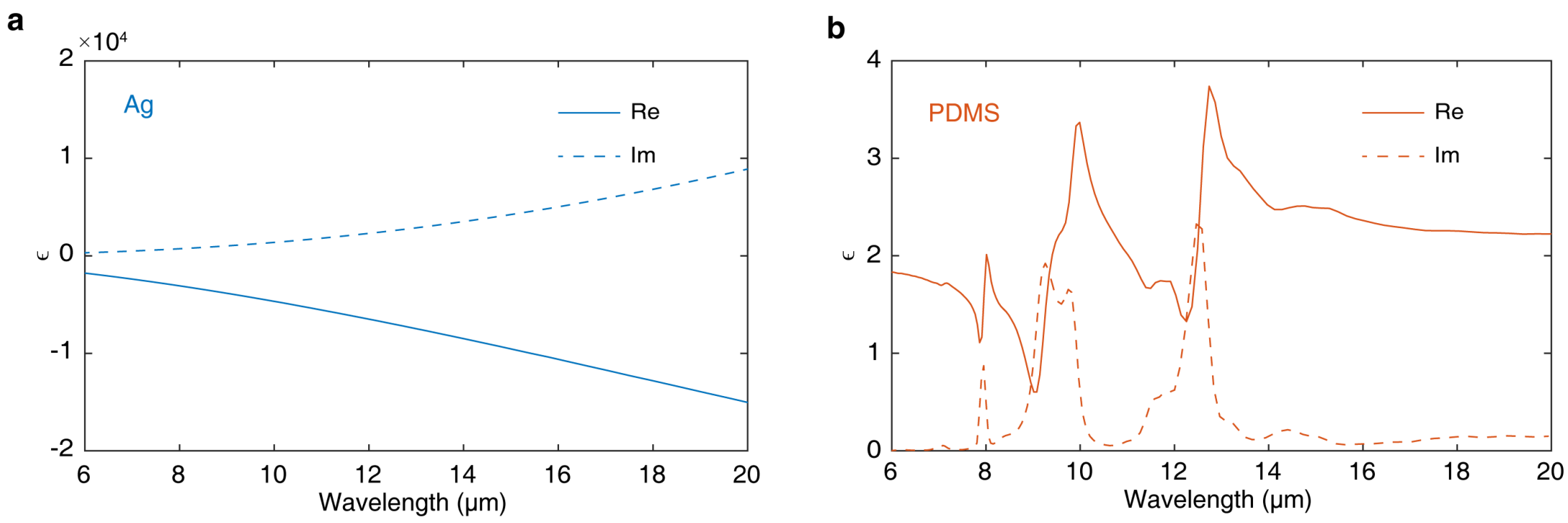

Supplementary Fig. 6. Permittivity of Ag and PDMS. a,b, Real (solid) and imaginary (dashed) parts of the permittivity for Ag (**a**) and PDMS (**b**) in the infrared wavelength

range. Ag exhibits negligible absorptivity in the infrared range, whereas PDMS shows strong infrared absorption.

Comment #3: Finally, foundational theoretical works on directional thermal emission and thermal antennas appears to be underrepresented or absent from the bibliography. Given the close conceptual connection to the present study, this omission weakens both the completeness of the references and the physical positioning of the work.

Response: We appreciate the reviewer's comment. In response, we have added relevant foundational references [1-5] in the revised manuscript to further improve the completeness of the bibliography and better position our work within the broader context of directional radiative heat transfer.

[1] Dahan, N. et al. Enhanced coherency of thermal emission: Beyond the limitation imposed by delocalized surface waves. *Phys. Rev. B* **76**, 045427 (2007).

[2] Biener, G., Dahan, N., Niv, A., Kleiner, V. & Hasman, E. Highly coherent thermal emission obtained by plasmonic bandgap structures. *Appl. Phys. Lett*. **92**, 081913 (2008).

[3] Laroche, M., Carminati, R. & Greffet, J.-J. Coherent Thermal Antenna Using a Photonic Crystal Slab. *Phys. Rev. Lett.* **96**, 123903 (2006).

[4] Schuller, J. A., Taubner, T. & Brongersma, M. L. Optical antenna thermal emitters. *Nat. Photonics* **3**, 658–661 (2009).

[5] Dahan, N., Gorodetski, Y., Frischwasser, K., Kleiner, V. & Hasman, E. Geometric Doppler Effect: Spin-Split Dispersion of Thermal Radiation. *Phys. Rev. Lett.* **105**, 136402 (2010).

We have made the below revisions in our revised manuscript.

Revision:

Main text:

Line 52 - 55 on page 2:

Although directional control of thermal radiation has been extensively investigated using various strategies, including phonon polariton gratings[30–32], photonic crystals[33–36], thermal antennas[37–39], metasurfaces[40–42], gradient epsilon-near-zero (ENZ) materials[43–47], and microstructures[48,49]

Reference:

31. Dahan, N. et al. Enhanced coherency of thermal emission: Beyond the limitation imposed by delocalized surface waves. *Phys. Rev. B* **76**, 045427 (2007).

35. Biener, G., Dahan, N., Niv, A., Kleiner, V. & Hasman, E. Highly coherent thermal emission obtained by plasmonic bandgap structures. *Appl. Phys. Lett.* **92**, 081913 (2008).
37. Laroche, M., Carminati, R. & Greffet, J.-J. Coherent Thermal Antenna Using a Photonic Crystal Slab. *Phys. Rev. Lett.* **96**, 123903 (2006).
38. Schuller, J. A., Taubner, T. & Brongersma, M. L. Optical antenna thermal emitters. *Nat. Photonics* **3**, 658–661 (2009).
39. Dahan, N., Gorodetski, Y., Frischwasser, K., Kleiner, V. & Hasman, E. Geometric Doppler Effect: Spin-Split Dispersion of Thermal Radiation. *Phys. Rev. Lett.* **105**, 136402 (2010).

Comment #4: Minor issues:
Additional limitations include the highly idealized nature of the 100% efficiency claim, the simplified treatment of convection and conduction, and the strong dependence of the reported performance on geometry and alignment. Consequently, the estimated energy savings are likely optimistic under realistic operating conditions.

Response: We appreciate the reviewer's comment. The 100% efficiency limit shown in Fig. 1 of the main text represents an idealized upper bound that highlights the fundamental advantage of directional radiative heat transfer. As the reviewer notes, practical implementations will inevitably deviate from this limit due to realistic constraints such as convection, conduction, and geometric configurations. To evaluate the performance under more realistic operating conditions and highlight the broad applicability of directional thermal emission, we have estimated the efficiency under varying parasitic heat transfer coefficients and geometric configurations.

Parasitic heat transfer losses

To quantify the impact of parasitic heat transfer losses on efficiency, we conducted simulations with varying parasitic heat transfer coefficients (Fig. R23). As the coefficients h increase to 3.5 and 7 W m^{-2} K^{-1}, the efficiency of System I decrease to 18% and 12%, respectively. In contrast, System III maintains the highest efficiencies through optimized transfer factors, reaching 46% and 25% under the same conditions.

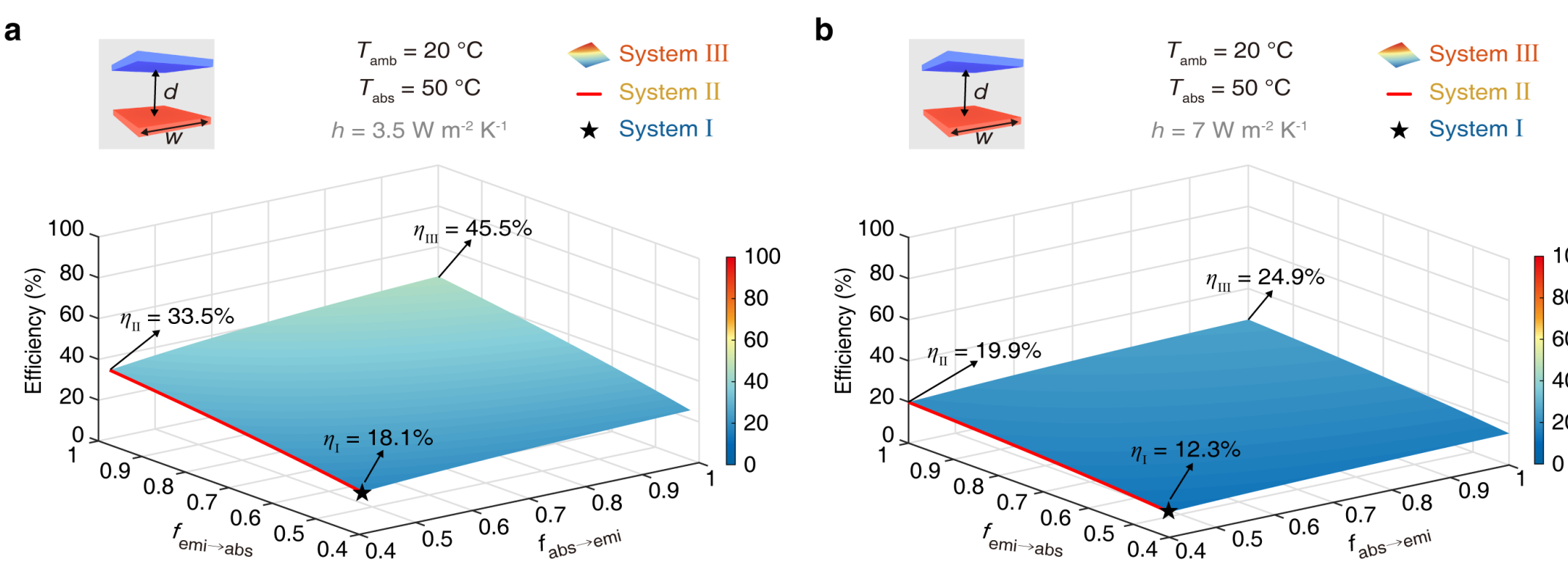

Fig. R23. Radiative heat transfer efficiency η under different parasitic heat transfer coefficients. a,b, Radiative heat transfer efficiency η for the three systems as a function of transfer factors $f_{emi \to abs}$ and $f_{abs \to emi}$ under parasitic heat transfer coefficients h of 3.5 (**a**) and 7 (**b**) W m^{-2} K^{-1}, respectively. Although parasitic heat transfer losses significantly reduce efficiency, System III still achieves the highest efficiency by optimizing the transfer factors. The ambient temperature and absorber temperature are set to T_{amb} = 20 °C and T_{abs} = 50 °C, respectively. Both the emitter and absorber have a width of w = 100 mm and are placed face-to-face with a separation distance of d = 50 mm.

We further analyzed the dependence of radiative heat transfer efficiency η on several key geometric parameters, including separation distance, relative orientation, and lateral misalignment (Fig. R24).

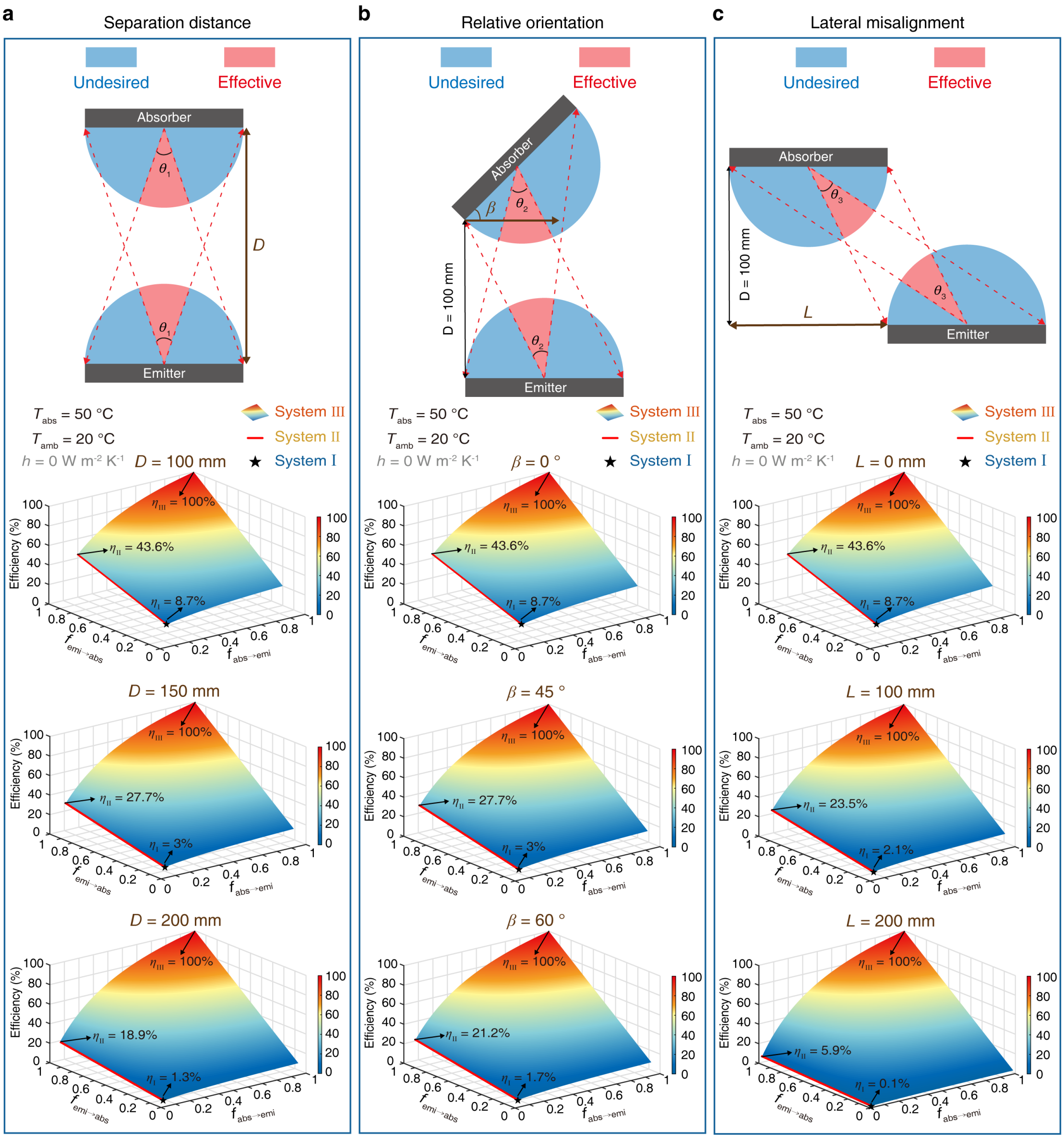

Fig. R24. Theoretical radiative heat transfer efficiency η under different geometric configurations. a, Schematic of two surfaces (100 mm by 100 mm) placed face-to-face with a separation distance of D. The theoretical radiative heat transfer

efficiencies of the three systems at different separation distances are shown below. **b,** Schematic of two surfaces (100 mm by 100 mm) oriented with a relative angle β at a fixed separation distance of 100 mm. The theoretical radiative heat transfer efficiencies of the three systems at different relative angles are shown below. **c,** Schematic of two surfaces (100 mm by 100 mm) with lateral misalignment L at a fixed separation distance of 100 mm. The theoretical radiative heat transfer efficiencies of the three systems at different lateral misalignments are shown below.

Separation distance

We consider an emitter placed face-to-face with an absorber, each with dimensions of 100 mm by 100 mm. As the separation distance D increases from 100 to 150 and 200 mm, the corresponding transfer factors $f_{\mathrm{emi}\to\mathrm{abs}}$ and $f_{\mathrm{abs}\to\mathrm{emi}}$ of System I decrease from 0.20 to 0.11 and 0.07, respectively, leading to efficiencies of 8.7%, 3.0%, and 1.3% (Fig. R24a). This clearly indicates that, in conventional blackbody systems, efficiency is strongly limited by separation distances and decreases rapidly as the distance increases. In contrast, at a separation distance of 200 mm, System II optimizes the directional features of the emitter according to the geometric configuration, increasing the transfer factor $f_{\mathrm{emi}\to\mathrm{abs}}$ from 0.07 to 1 and thereby improving efficiency from 1.3% to 18.9%. System III further achieves the theoretical upper limit of 100% efficiency by simultaneously optimizing both $f_{\mathrm{emi}\to\mathrm{abs}}$ and $f_{\mathrm{abs}\to\mathrm{emi}}$.

Relative orientation

We consider an emitter with a relative angle β of 0°, 45° and 60° to the absorber, at a fixed separation of 100 mm and identical dimensions of 100 mm by 100 mm. As the relative angle increases, the transfer factors $f_{\mathrm{emi}\to\mathrm{abs}}$ and $f_{\mathrm{abs}\to\mathrm{emi}}$ of System I decrease from 0.20 to 0.11 and 0.08, respectively, leading to efficiencies of 8.7%, 3.0%, and 1.7% (Fig. R24b). At a relative angle of 60°, System II increases the transfer factor $f_{\mathrm{emi}\to\mathrm{abs}}$ from 0.08 to 1, thereby improving efficiency from 1.7% to 21.2%, while System III reaches the maximum theoretical efficiency of 100% by optimizing both $f_{\mathrm{emi}\to\mathrm{abs}}$ and $f_{\mathrm{abs}\to\mathrm{emi}}$.

Lateral misalignment

We consider a face-to-face configuration with lateral misalignments L of 0, 100, and 200 mm, with both emitter and absorber having dimensions of 100 mm by 100 mm. As the lateral misalignment increases, the transfer factors $f_{\mathrm{emi}\to\mathrm{abs}}$ and $f_{\mathrm{abs}\to\mathrm{emi}}$ of System I decrease from 0.20 to 0.09 and 0.02, respectively, leading to efficiencies of 8.7%, 2.1%, and 0.1% (Fig. R24c). At a lateral misalignment of 200 mm, System II enhances the transfer factor $f_{\mathrm{emi}\to\mathrm{abs}}$ to 1, increasing the efficiency from 0.1% to 5.9%, while System III consistently maintains the theoretical maximum efficiency of 100%.

Overall, these results highlight that Systems II and III mitigate the sensitivity to geometric variations through directional thermal emission, demonstrating the geometric robustness and broad applicability of our framework.

Moreover, although the achievable efficiency under realistic conditions is reduced by non-ideal directional control, as well as conductive and convective losses, the experimental results (Figs. 3-4 in the main text) still demonstrate substantial energy savings of 28.1%-28.7% in indoor environment (Fig. 3 in the main text) and 17.4%-22.9% in vehicle applications (Fig. 4 in the main text). These results indicate that directional radiative heat transfer can maintain significant performance advantages under practical operating conditions. With continued advances in directional thermal emission control and further mitigation of conductive and convective losses, the energy-saving potential could be further enhanced.

We have made the below revisions in our revised manuscript.

Revision:

Main text:

Line 161 - 164 on page 5:

Notably, in practical radiative heat transfer scenarios, despite constraints such as parasitic heat transfer losses (Supplementary Fig. 3) and geometric configurations (Supplementary Note 3 and Supplementary Fig. 4), System III consistently achieves the highest efficiency.

Supplementary Information:

Supplementary Note 3: Radiative heat transfer efficiency η under different geometric configurations

To clarify the impact of geometric factors on radiative exchange, we have analyzed the dependence of radiative heat transfer efficiency η on several key parameters, including separation distance, relative orientation, and lateral misalignment.

First, we consider an emitter placed face-to-face with an absorber, each with dimensions of 100 mm by 100 mm. As the separation distance D increases from 100 to 150 and 200 mm, the corresponding transfer factors $f_{\text{emi}\to\text{abs}}$ and $f_{\text{abs}\to\text{emi}}$ of System I decrease from 0.20 to 0.11 and 0.07, respectively, leading to efficiencies of 8.7%, 3.0%, and 1.3% (Supplementary Fig. 4a). This clearly indicates that, in conventional blackbody systems, efficiency is strongly limited by separation distances and decreases rapidly as the distance increases. In contrast, at a separation distance of 200 mm, System II optimizes the directional features of the emitter according to the geometric configuration, increasing the transfer factor $f_{\text{emi}\to\text{abs}}$ from 0.07 to 1 and thereby improving efficiency from 1.3% to 18.9%. System III further achieves the theoretical upper limit of 100% efficiency by simultaneously optimizing both $f_{\text{emi}\to\text{abs}}$ and $f_{\text{abs}\to\text{emi}}$.

Second, we consider an emitter with a relative angle β of 0°, 45° and 60° to the absorber, at a fixed separation of 100 mm and identical dimensions of 100 mm by 100 mm. As the relative angle increases, the transfer factors $f_{\text{emi}\to\text{abs}}$ and $f_{\text{abs}\to\text{emi}}$ of System I

decrease from 0.20 to 0.11 and 0.08, respectively, leading to efficiencies of 8.7%, 3.0%, and 1.7% (Supplementary Fig. 4b). At a relative angle of 60°, System II increases the transfer factor $f_{\mathrm{emi}\to\mathrm{abs}}$ from 0.08 to 1, thereby improving efficiency from 1.7% to 21.2%, while System III reaches the maximum theoretical efficiency of 100% by optimizing both $f_{\mathrm{emi}\to\mathrm{abs}}$ and $f_{\mathrm{abs}\to\mathrm{emi}}$.

Third, we consider a face-to-face configuration with lateral misalignments L of 0, 100, and 200 mm, with both emitter and absorber having dimensions of 100 mm by 100 mm. As the lateral misalignment increases, the transfer factors $f_{\mathrm{emi}\to\mathrm{abs}}$ and $f_{\mathrm{abs}\to\mathrm{emi}}$ of System I decrease from 0.20 to 0.09 and 0.02, respectively, leading to efficiencies of 8.7%, 2.1%, and 0.1% (Supplementary Fig. 4c). At a lateral misalignment of 200 mm, System II enhances the transfer factor $f_{\mathrm{emi}\to\mathrm{abs}}$ to 1, increasing the efficiency from 0.1% to 5.9%, while System III consistently maintains the theoretical maximum efficiency of 100%. Overall, these results highlight that Systems II and III mitigate the sensitivity to geometric variations through directional thermal emission, thereby demonstrating the geometric robustness and broad applicability of our framework.

Supplementary Fig. 3:

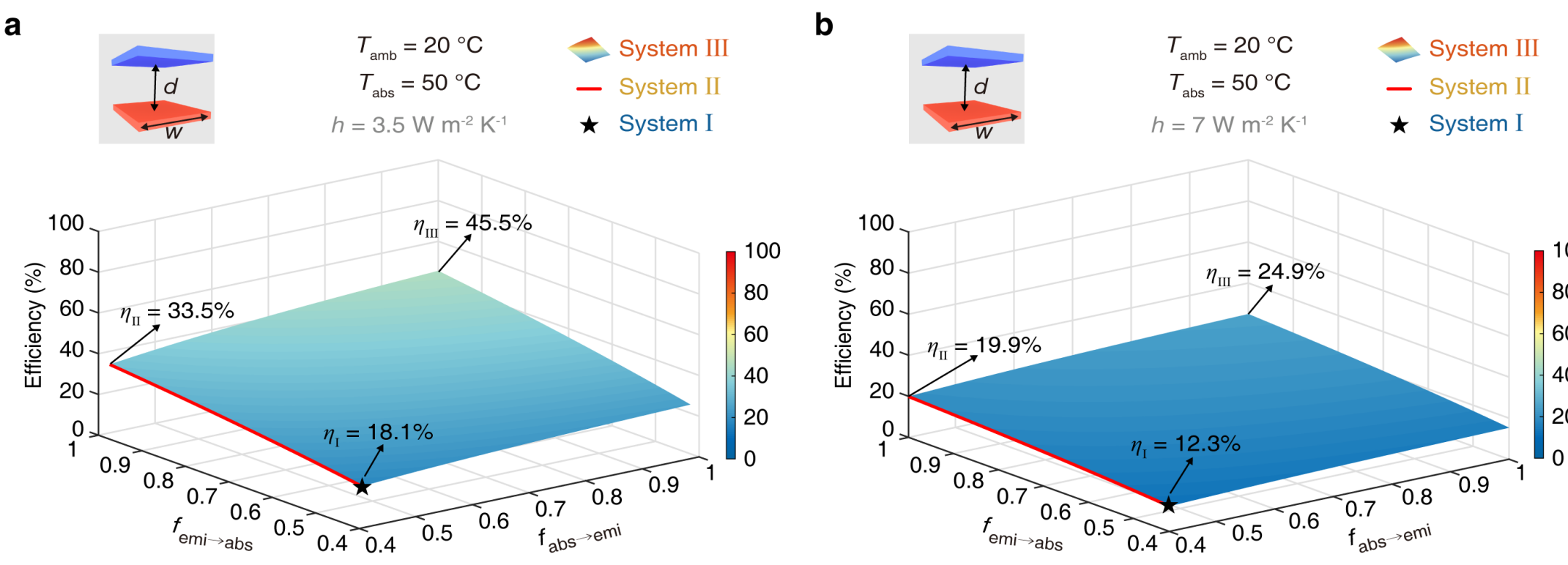

Supplementary Fig. 3. Radiative heat transfer efficiency η under different parasitic heat transfer coefficients. a,b, Radiative heat transfer efficiency η for the three systems as a function of transfer factors $f_{\mathrm{emi}\to\mathrm{abs}}$ and $f_{\mathrm{abs}\to\mathrm{emi}}$ under parasitic heat transfer coefficients h of 3.5 (**a**) and 7 (**b**) W m^{-2} K^{-1}, respectively. Although parasitic heat transfer losses significantly reduce efficiency, System III still achieves the highest efficiency by optimizing the transfer factors. The ambient temperature and absorber temperature are set to T_{amb} = 20 °C and T_{abs} = 50 °C, respectively. Both the emitter and absorber have a width of w = 100 mm and are placed face-to-face with a separation distance of d = 50 mm.

Supplementary Figure 4:

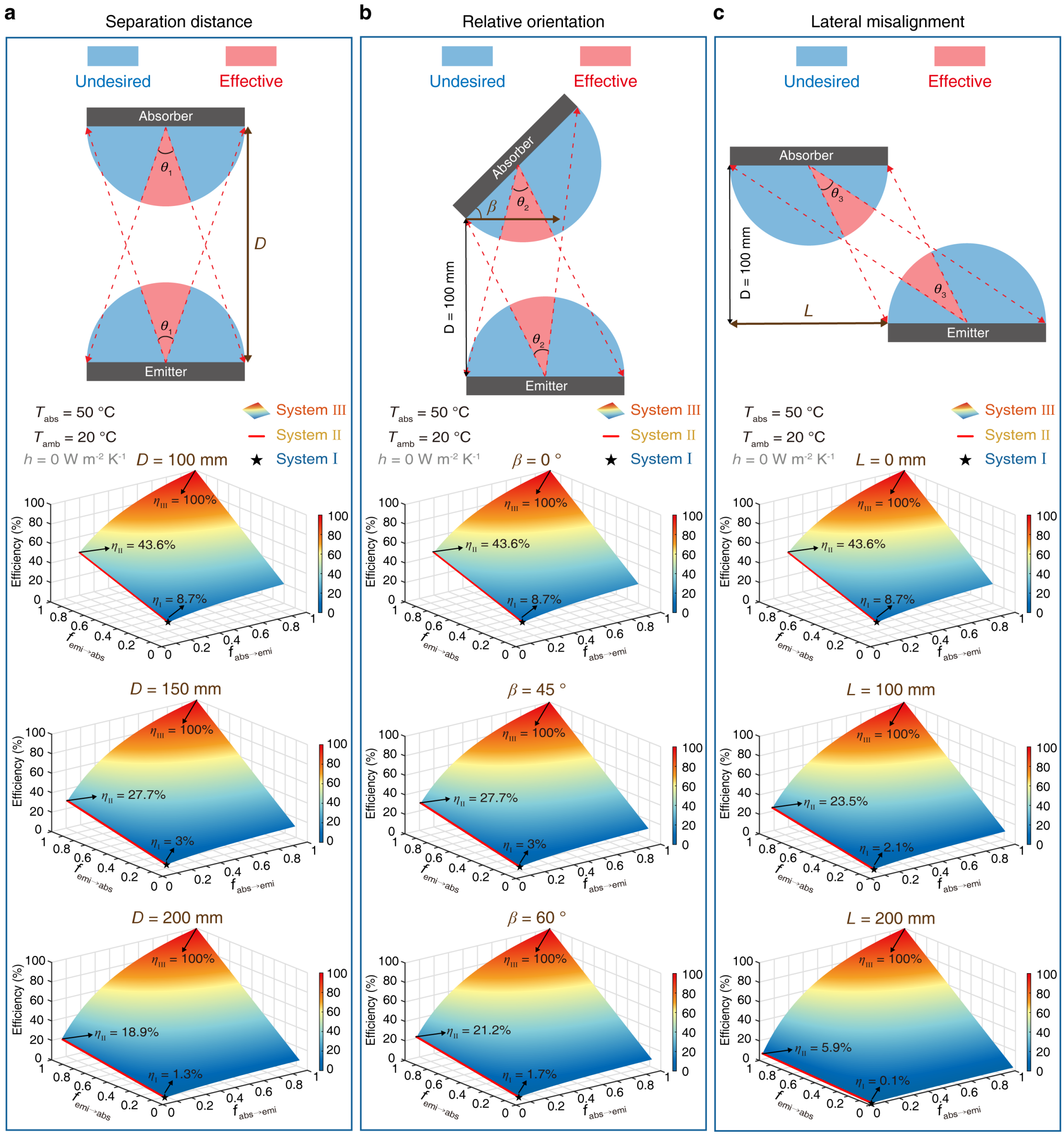

Supplementary Fig. 4. Theoretical radiative heat transfer efficiency η under different geometric configurations. a, Schematic of two surfaces (100 mm by 100 mm) placed face-to-face with a separation distance of *D*. The theoretical radiative heat transfer efficiencies of the three systems at different separation distances are shown below. **b,** Schematic of two surfaces (100 mm by 100 mm) oriented with a relative angle β at a fixed separation distance of 100 mm. The theoretical radiative heat transfer efficiencies of the three systems at different relative angles are shown below. **c,** Schematic of two surfaces (100 mm by 100 mm) with lateral misalignment *L* at a fixed separation distance of 100 mm. The theoretical radiative heat transfer efficiencies of the three systems at different lateral misalignments are shown below.

We extend our deep gratitude once again to the reviewer for the constructive suggestions and for supporting the potential publication of our work in *Nature Communications*. We have carefully addressed these comments in a point-to-point manner as above. We hope the reviewer will find our responses satisfactory.

Response to the Reviewers' Comments on the manuscript entitled 'Directional thermal emission enables efficient energy savings' submitted to *Nature Communications*

We extend our sincere appreciation to the editor and all reviewers for their careful review of our manuscript and positive feedback on our manuscript. We are grateful for their constructive suggestions to further enhance the quality of our work and for their supporting the publication of our work. In the response letter below, we address each comment of the reviewers, and append a revised manuscript with all revisions highlighted in yellow. With these revisions implemented, we believe that our manuscript in alignment with the standards for publication in *Nature Communications*.

Response to Reviewer #1:

Reviewer #1:

The authors have thoroughly addressed all of my comments and concerns. The revised manuscript and Supplementary Information have substantially improved the quality, completeness, and rigor of the work. In my view, the paper is now ready for publication in Nature Communications.

Response: We sincerely appreciate the reviewer for the insightful comments and constructive suggestions throughout the review process, as well as for supporting the publication of our manuscript in *Nature Communications*.

Response to Reviewer #3:

Reviewer #3: I co-reviewed this manuscript with one of the reviewers who provided the listed reports. This is part of the Nature Communications initiative to facilitate training in peer review and to provide appropriate recognition for Early Career Researchers who co-review manuscripts.

Response: We sincerely appreciate the reviewer for participating in the co-review process and contributing to the evaluation of our manuscript, as well as for the reviewer's time and effort in supporting the peer review process.

Response to Reviewer #4:

Reviewer #4: The revised version is ready for publication.

Response: We sincerely appreciate the reviewer for the insightful comments and constructive suggestions throughout the review process, as well as for supporting the publication of our manuscript in *Nature Communications*.

Response to Reviewer #5:

Reviewer #5: Overall the authors have significantly improved their manuscript. However several aspects of the physics should be clarified further to strengthen the manuscript.

Response: We sincerely appreciate the reviewer for the constructive suggestions and for recognizing the substantial improvements made to our work.

Comment #1: -The manuscript sometimes gives the impression that the efficiency enhancement is fundamentally new physics, whereas much of the gain originates from geometric/angular confinement of radiation. It would help to emphasize more explicitly that the key novelty is the extension of radiative transfer theory to directional emitters/absorbers and its practical implementation.

Response: We appreciate the reviewer's suggestion. In response, we have revised the manuscript to clarify that our work extends classical radiative transfer theory to directional radiative transfer systems, improving the clarity of the relevant discussion and avoiding potential misunderstandings.

We have made the below revisions in our revised manuscript.

Revision:

Main text:

Line 61- 65 on page 3:

In this work, we extend radiative transfer theory to directional radiative transfer systems by establishing the relationship between directional emission features and radiative heat transfer efficiency, demonstrating that an ideal system with perfectly matched directional thermal emission between an emitter and absorber can reach a theoretical efficiency up to 100%.

Comment #2: -The distinction between "directional thermal emission" and "true thermal antennas" is not sufficiently discussed. The manuscript cites ENZ thermal antennas and broadband directional emitters, but the physical connection between these previous works and the present macroscopic radiative-transfer framework should be explained more clearly.

Response: We appreciate the reviewer's suggestion. "Directional thermal emission" is a key factor determining the overall radiative heat transfer efficiency. In this work, it is treated as a macroscopic radiative property governing energy exchange efficiency, rather than being associated with any specific physical implementation or device mechanism. By contrast, "true thermal antennas", including ENZ thermal antennas and broadband directional emitters, represent device-level strategies for engineering such

directional emission features. These previous works therefore serve as practical routes for realizing the directional emission conditions considered in the proposed radiative transfer framework, rather than constituting the framework itself. We have further clarified in the revised manuscript that the ability to tailor directional emission features provides the physical basis for efficient radiative heat transfer.

We have made the below revisions in our revised manuscript.

Revision:

Main text:

Line 195 - 201 on page 8:

Previous advances in directional thermal emission control have enabled the realization of diverse directional emission features, providing the basis for selecting and implementing the specific emission features considered in this work. Accordingly, we consider two representative radiative heat transfer scenarios and utilize two previously reported directional emitters[50,51] with specific directional emission features to demonstrate the efficiency enhancement (Fig. 2b,c and Supplementary Figs. 5-6).

Comment #3: -Equation (2) is central to the paper, but the derivation and physical meaning of the transfer factor deserve more intuition. A short discussion explaining its physical meaning would greatly improve readability.

Response: We appreciate the reviewer's suggestion. The transfer factor is introduced as a generalization of the conventional view factor for systems involving directional emission and absorption. It represents the fraction of radiative power emitted by one surface that is effectively intercepted and absorbed by another, while explicitly accounting for angular, spectral, and spatial distributions of emission and absorption. In this sense, it describes the effective radiative exchange between two surfaces beyond purely geometric considerations.

We have made the below revisions in our revised manuscript.

Revision:

Main text:

Line 102 - 107 on page 4:

Unlike the conventional view factor, which depends solely on geometry under the assumption of blackbody systems, the transfer factor additionally accounts for directional emission and absorption features. Therefore, it quantifies the effective fraction of radiative power emitted by the emitter that is ultimately intercepted and absorbed by the target absorber, incorporating both geometric alignment and directional emission features.

Supplementary Information:

Supplementary Note 1:

Line 44 - 51 on page 2:

The transfer factor is calculated by integrating the radiative exchange between all interacting differential surface elements of the emitter and absorber over angle, wavelength, and surface area while accounting for directional emission and absorption features. Therefore, it quantifies the effective fraction of radiative power emitted by the emitter that is ultimately intercepted and absorbed by the target absorber, thereby providing a generalized framework for analyzing radiative heat transfer in systems with arbitrary geometric configurations and directional emission features.

Comment #4: -The practical applications section is convincing from an engineering perspective, but some claims could be toned down slightly. For example, the link to "global carbon neutrality and sustainability" appears several times and may sound broader than what is directly demonstrated experimentally.

Response: We appreciate the reviewer's suggestion. We have revised the relevant text to provide a more balanced and accurate description, thereby improving the overall rigor of the manuscript.

We have made the below revisions in our revised manuscript.

Revision:

Main text:

Line 34 - 36 on page 2:

Our work highlights the broad applicability and energy-saving potential of directional radiative heat transfer, opening new avenues for efficient energy utilization.

Line 75 - 79 on page 3:

Our work not only explores the potential of utilizing directional thermal emission to enhance radiative heat transfer efficiency, but also provides design guidelines for desired directional emission features enabled by photonic and metamaterial structures, contributing to advanced thermal management and efficient energy utilization.

Line 361 - 365 on page 14:

In particular, driven by advances in thermal radiation control technologies related to thermal cycling durability[58,59], large-area fabrication and integration[60,61], directional radiative heat transfer is expected to become increasingly feasible for real-world deployment, offering a pathway toward advanced thermal management and efficient energy utilization.

Comment #5: Scientifically, the manuscript would also benefit from citing and discussing several missing papers. The following missing references are important and should be cited because they cover several key advances that are directly related to the present work but are not adequately represented in the current bibliography, the first explicit formulation of a "true thermal antenna" based on hyperbolic metamaterials (Barbillon et al.) and thermally tunable broadband directional emitters (Hwang et al.),

- J. S. Hwang, J. Xu, and A. P. Raman, "Thermally Tunable Angular Selectivity of Broadband Directional Thermal Emission," Nano Lett. 25, 8064 (2025).

- G. Barbillon, J.-P. Hugonin, S.-A. Biehs, and P. Ben-Abdallah, "True thermal antenna with hyperbolic metamaterials," Opt. Express 25, 23356 (2017).

Response: We appreciate the reviewer's suggestion. In response, we have added the recommended references [1-2] in the revised manuscript to further improve the completeness of the bibliography.

[1] Barbillon, G., Sakat, E., Hugonin, J.-P., Biehs, S.-A. & Ben-Abdallah, P. True thermal antenna with hyperbolic metamaterials. *Opt. Express* **25**, 23356 (2017).

[2] Hwang, J. S., Xu, J. & Raman, A. P. Thermally Tunable Angular Selectivity of Broadband Directional Thermal Emission. *Nano Lett.* **25**, 8064–8071 (2025).

We have made the below revisions in our revised manuscript.

Revision:

Main text:

Line 52 - 58 on page 2:

Although directional control of thermal radiation has been extensively investigated using various strategies, including phonon polariton gratings[30–32], photonic crystals[33–36], thermal antennas[37–40], metasurfaces[41–43], gradient epsilon-near-zero (ENZ) materials[44–49], and microstructures[50,51], previous studies have primarily focused on structural design of emitters to control specific directional emission features, while the impact of these features on radiative heat transfer efficiency remains elusive.

Reference:

40. Barbillon, G., Sakat, E., Hugonin, J.-P., Biehs, S.-A. & Ben-Abdallah, P. True thermal antenna with hyperbolic metamaterials. *Opt. Express* **25**, 23356 (2017).
49. Hwang, J. S., Xu, J. & Raman, A. P. Thermally Tunable Angular Selectivity of Broadband Directional Thermal Emission. *Nano Lett.* **25**, 8064–8071 (2025).

We extend our deep gratitude once again to the reviewer for providing constructive suggestions. All comments have been carefully addressed, with point-by-point revisions and responses provided as above. We hope the reviewer will find our responses satisfactory.
